# A bifunctional endolytic alginate lyase with two different lyase catalytic domains from *Vibrio* sp. H204

**DOI:** 10.3389/fmicb.2024.1509599

**Published:** 2024-12-13

**Authors:** Chune Peng, Qingbin Wang, Wei Xu, Xinkun Wang, Qianqian Zheng, Xiaohui Liang, Xiaodan Dong, Fuchuan Li, Lizeng Peng

**Affiliations:** ^1^Key Laboratory of Agro-Products Processing Technology of Shandong Province, Key Laboratory of Novel Food Resources Processing Ministry of Agriculture, Institute of Food & Nutrition Science and Technology, Shandong Academy of Agricultural Sciences, Jinan, China; ^2^National Glycoengineering Research Center, Shandong Provincial Key Laboratory of Carbohydrate Chemistry and Glycobiology, State Key Laboratory of Microbial Technology, Shandong University, Qingdao, China; ^3^School of Life Sciences, Qilu Normal University, Jinan, China; ^4^Henan Key Laboratory of Immunology and Targeted Drugs, School of Laboratory Medicine, Xinxiang Medical University, Xinxiang, China; ^5^Energy Research Institute, Qilu University of Technology (Shandong Academy of Sciences), Jinan, China

**Keywords:** alginate lyase, bifunctional, catalytic domain, oligosaccharides, marine bacterium

## Abstract

Alginate lyases can fully degrade alginate into various size-defined unsaturated oligosaccharide products by *β*-elimination. Here, we identified the bifunctional endolytic alginate lyase Aly35 from the marine bacterium *Vibrio* sp. Strain H204. The enzyme Aly35 is classified into the polysaccharide lyase 7 superfamily and contains two alginate lyase catalytic domains. The relationship and function of the two lyase domains are not well known. Thus, the full-length recombinant enzyme and its truncated proteins Aly35-CD1 (catalytic domain 1), Aly35-CD2 (catalytic domain 2 domain) were constructed. The three enzymes showed similar biochemical characteristics and exhibited temperature and pH stability. Further research showed that Aly35 and Aly35-CD2 can efficiently degrade alginate, polymannuronate (PM) and polyguluronate (PG) into a series of unsaturated oligosaccharides, while Aly35-CD1 exhibits greater PM-degrading activity than that of Aly35-CD2 but can not degraded PG efficiently. The results suggest that the domain (Trp^295^-His^582^) is critical for PG-degrading activity, the domain has (Leu^53^-Lys^286^) higher PM-degrading activity, both catalytic domains together confer increased alginate (including M-blocks and G blocks)-degrading activity. The enzyme Aly35 and its truncations Aly35-CD1 and Aly35-CD2 will be useful tools for structural analyses and for preparing bioactive oligosaccharides, especially Aly35-CD1 can be used to prepare G unit–rich oligosaccharides from alginate.

## Introduction

Alginate, which can be extracted from brown seaweed, is the major component of the algal cell wall matrix, accounts for up to 40% of the dry weight of brown seaweed (Mrudulakumari et al., 2021), represents a large amount of marine biomass and is an important source of marine carbon cycling (Zhu et al., 2021). Alginate is a linear polyanionic polysaccharide composed of (1–4)-linked *β*-D-mannuronate (M) and its C5-epimer *α*-L-guluronate (G) (Gao et al., 2021). These two uronate residues are linked homogeneously or heterogeneously and comprise three types of blocks: PM blocks, PG blocks and hetero-oligomeric polyMG or PolyGM blocks (PMG) (Wong et al., 2000; Pawar and Edgar, 2012). Acetylated alginate is usually synthesized by *Azotobacter* and *Pseudomonas* strains to protect the bacteria from drugs or environmental damage (Rehm and Valla, 1997; Remminghorst and Rehm, 2006; Rehm, 2010). Algal alginate is widely used as a viscosity-enhancing hydrocolloid, stabilizer, gelling agent, food, cosmetic and pharmaceutical additive (Ścieszka and Klewicka, 2019; Li et al., 2021; Bi et al., 2022; Khan et al., 2022; Zhang et al., 2023). Alginate oligomers with different degrees of polymerization are called alginate oligosaccharides (AOS) (Xing et al., 2020). AOS exhibit several advantages, as they do not pollute the environment, absorb easily, and exhibit a small molecular weight and good water solubility (Lu et al., 2022). Moreover, AOSs exhibit various biological activities, such as antitumor (Bi et al., 2023), antibacteria and antioxidant (Ning et al., 2022), antivirus (Serrano-Aroca et al., 2021), anti-inflammation, immunemodulatory (Ghanta et al., 2020), and immune inducer (Zhang et al., 2019) effects. Therefore, AOSs have attracted increasing interest for various applications, and were widely used in health care, medical and pharmaceutical (Gao et al., 2021), and food (Martău et al., 2019).

Alginate lyases cleave the glycosidic bonds in alginate through a *β*-elimination reaction to generate oligosaccharides that contain unsaturated sugar units at the non-reducing end with characteristic absorption at 232 nm (Lombard et al., 2010; Ertesvåg, 2015). The newly formed unsaturated residues are derived from G or M units but lose their original configurations; thus, they are designated Δ units (Peng et al., 2018a, 2018b). Alginate lyases have wide application prospects in AOS reparation (Barzkar et al., 2022), sequence research (Ostgaard et al., 1993; Zhang et al., 2006), medical treatment (Islan et al., 2013), seaweed waste disposal (Barzkar et al., 2022), and biofuel preparation (MacDonald et al., 2016; Jagadevan et al., 2018). Thus far, numerous alginate lyases have been identified from marine organisms (algae, mollusks, bacteria, and fungi), terrestrial bacteria, and some viruses, with microbial sources being the most abundant (Peng et al., 2018a,b). According to the Carbohydrate-Active enZYmes (CAZy) database,^1^ alginate lyases are distributed into polysaccharide lyase (PL) 5, 6,7, 8, 14, 15, 17, 18, 31, 32, 34, 36, 39, and 41 families based on their amino acid sequences (Zhu and Yin, 2015; Drula et al., 2022). Most alginate lyases are classified into the PL-5, 6, 7, 14, 15, 17, and 18 families, and the PL7 family has the highest number of alginate lyases (Xu et al., 2020). Furthermore, based on their substrate preference, alginate lyases are classified into three groups, namely, PM-specific lyases (EC4.2.2.3), PG-specific lyases (EC4.2.2.11), and bifunctional lyases can degrade both PM and PG (EC 4.2.2.-) (Xu et al., 2018). According to the mode of action, alginate lyases can be grouped into endotype lyases, which generate a series of oligosaccharides with different degrees of polymerization by cleaving the inside-chain glycosidic bonds (Peng et al., 2018a,b), or exotype lyases, which produce monomers or dimers by gradual degradation from the end of alginate (Suzuki et al., 2006; Kim et al., 2012; Cao et al., 2023). The endotype and exotype of alginate lyase-encoding genes often coexist in the same genome and work synergistically to quickly and fully digest alginate, thus providing energy and carbon sources for microorganisms (Lu et al., 2019). In addition, alginate lyases can be used to produce ethanol through the efficient degradation of alginate (Wargacki et al., 2012; Cao et al., 2022). However, the synergy between different catalytic domains of a single lyase has rarely been reported.

Alginate lyases usually have diverse domain compositions, such as N-terminal carbohydrate-binding modules (CBMs) that can enhance the thermostability of enzymes (Cheng et al., 2017), an N-terminal catalytic domain, a C-terminal catalytic domain, and an extra C-terminal domain essential for enzyme dimerization (Xu et al., 2017). However, most of the multimodular alginate lyases reported thus far contain only a single functional catalytic domain. There are only a few enzymes with two lyase catalytic domains, such as Algb from *Vibrio* sp. W13, which contains two hypothetical alginate lyase domains (Zhu et al., 2015), however, the two domains of this enzyme has not been verified. AlyC6’ from *Vibrio* sp. NC2 possesses two alginate lyase catalytic domains, and these two domains perform synergistic functions (Wang et al., 2022). AlyC8 from *Vibrio* sp. C42 also has two catalytic domains with synergistic effects (Sun et al., 2022).

In this study, a PL7 alginate lyase, Aly35, with two alginate lyase catalytic domains, was identified from a marine alginate-degrading bacterium, *Vibrio* sp. Strain H204 (Chinese invention patent number: ZL202310428638.4), which was isolated from marine mud in Shandong Province, China. The whole protein sequence and two gene-truncated versions (Aly35-CD1 and Aly35-CD2) were subsequently cloned from its genome. Furthermore, the biochemical characteristics, substrate-degrading patterns, and oligosaccharide-yielding properties of the three enzymes were compared.

## Experimental procedures

### Materials and strains

Sodium alginate (alginic acid sodium salt from brown algae, medium viscosity) was purchased from Sigma–Aldrich Co., Ltd., USA. PG and PM (purity of approximately 95%), were purchased from Qingdao BZ Oligo Biotech Co. Ltd. (Qingdao, China). The bacterial strains and plasmid vectors used in this study are listed in Table 1. Prime STAR™ HS DNA polymerase, restriction endonuclease, T4 DNA ligase, and other genetic engineering enzymes were purchased from TaKaRa Inc. (Dalian, China). A Superdex 30 Increase 10/300 GL column was purchased from Cytiva. All of the other chemicals and reagents were of the highest quality available.

**Table 1:** Bacterial strains, plasmids, and primers used for sequencing in the present study.

Strain or plasmid	Description	Source
*Vibro* sp. H204	A Alginate-degrading marine bacterium (patent deposit number: CGMCC25216)	This study
*E. coli* BL21(DE3)	F-, ompT, hsdSB (rB-, mB-), dcm, gal, λ (DE3), pLysS, Cm^r^	Novagen
Plasmid		
pET30a	Expression vector; Kan^r^	Novagen
pET30a-Aly35	pET30a carrying an amplified *BamH* I-*Xho* I fragmnt encoding the recombinant protein of Aly35 fused with a His_6_ tag at the N terminus	This study
Sequencing primers		
Aly35-F	5’-CGGGATCCTGTGGAGGAAATACCGCC-3’	
Aly35-R	5′- CCCTCGAGCTATTGATGAAGAGTGCTC -3’	
Aly35-CD1-F	5’-CGGGATCCTGTGGAGGAAATACCGCC-3’	
Aly35-CD1-R	5’-CCCTCGAGCTATTTGGTTAGTCCTAACTG-3’	
Aly35-CD2-F	5’-CGGGATCCTGGAATATTGACGATTGGAAATTAAC-3’	
Aly35-CD2-R	5’-CCCTCGAGCTATTGATGAAGAGTGCTC-3’	

Restriction enzyme sites are underlined; Kan^r^, kanamycin-resistant.

### Analysis of gene and Protein sequence of alginate Lyase

The GC content (GC%) of the open reading frame (ORF) and the sequence alignment were calculated using Bio-Edit version 7.2.5. The Aly35 amino acids sequence was submitted on NCBI,^2^ the domain and similar sequences were analyzed using the BLASTp algorithm. The molecular mass of the protein was estimated using the peptide mass tool on the ExPASy server of the Swiss Institute of Bioinformatics.^3^ Protein modules and domains were predicted using the Simple Modular Architecture Research Tool (SMART). The signal peptide and its type were identified using the SignalP 5.0 server and the LipoP 1.0 server. Multiple sequence alignments were performed using Bio-Edit version 7.2.5. AlphaFold2^4^ was used to predict the structure of Aly35 by using A0A066V096.1. A Lyase AlphaFold DB model of A0A066V096_9VIBR (gene: A0A066V096_9VIBR, organism: *Vibrio fortis*) as template, then structures were visualized and analyzed with PyMOL Version 2.1.1 (Du et al., 2024). The phylogenetic tree of alginate lyases Aly35 constructed by phylogeny.fr online web service, through alignment of protein sequences by blast analysis and scoring of alignments between protein sequences (Supplementary Table S1) was performed by BLOSUM62 matrix and the evolution was analyzed by PhyML v3.0^5^ algorithm which is a simple, fast and accurate to estimate large phylogenies by maximum like-hood robust method.

### Heterologous expression of Aly35 and the truncated protein

The genome of the *Vibrio* sp. strain H204 isolated from Seamud encodes at least four putative alginate lyases. To express the recombinant Aly35 protein and its truncated proteins Aly35-CD1 (Cys^21^-Lys^286^) and Aly35-CD2 (Trp^296^-His^582^) in *E. coli* strains, the full-length gene without the signal peptide sequence was amplified using high fidelity Prime STAR™ HS DNA polymerases and the primer pair Aly35-F and Aly35-R, as listed in Table 1. The truncated gene was amplified using the primer pair Aly35-CD1-F (same as Aly35-F) and Aly35-CD1-R, and the another truncated gene was amplified using the primer pair Aly35-CD2-F and Aly35-CD2-R (same as Aly35-R). Primer pairs with the restriction enzyme site *BamH*I-*Xho*I (underlined in Table 1) were designed according to the insertion site sequences of the expression plasmids pET-30a (+) (Novagen) following a T7 promoter, with a His_6_ tag at the N-terminus of the recombinant protein of Aly35, Aly35-CD1 or Aly35-CD2. The recombinant plasmid (pE30a-Aly35, pE30a-Aly35-CD1 or pE30a-Aly35-CD2) was amplified in *E. coli* DH5α cells and then individually transformed into *E. coli* BL21(DE3) cells for protein expression. The integrity of the nucleotide sequences of all constructed plasmids were confirmed by DNA sequencing.

Recombinant full-length Aly35 (WP_132936395.1) and its truncated protein were expressed and purified as described previously (Peng et al., 2018a,b). Briefly, *E. coli* cells harboring a recombinant plasmid were initially cultured in 5 mL of LB broth supplemented with 50 μg/mL kanamycin. When the cell density reached an OD_600_ of 0.6–0.8, target protein expression was induced by the addition of 0.1 mM isopropyl 1-thio-*β*-D-galactopyranoside at a final concentration of 0.05 mM. After culturing for another 24 h at 16°C, the cells were harvested by centrifugation at 6,000 × g for 15 min. The pellet was resuspended twice using ice-cold buffer A [50 mM Tris–HCl, 150 mM NaCl (pH 8.0)] and disrupted by sonication (60 repetitions, 5 s) in an ice-cold environment. After centrifugation at 15,000 × g for 30 min, the supernatant was collected for further purification of the target proteins.

### Purification of the recombinant proteins Aly35, Aly35-CD1, and Aly35-CD2

The supernatant containing each recombinant protein was loaded onto a column packed with nickel-Sepharose™ 6 Fast Flow resin (GE Healthcare, USA). After impurities were removed by washing with buffer A containing 20 mM imidazole, the target protein was eluted with a gradient concentration of imidazole ranging from 50 to 250 mM. The presence and purity of the proteins in each fraction were analyzed by SDS–PAGE according to the method previously reported method (Brunelle and Green, 2014). The proteins were visualized by staining using Coomassie Brilliant Blue R-250. The imidazole was removed by ultrafiltration and the protein concentration was determined by using a bicinchoninic acid (BCA) kit.

### Biochemical characteristics of the recombinant proteins Aly35

To determine the optimal pH for the activity of the recombinant enzymes, sodium alginate (1 mg/mL) was digested with 1 μg of Aly35 in three different buffer systems: 50 mM NaAc-HAc (pH 5.0–6.0), NaH_2_PO_4_-Na_2_HPO_4_ (pH 6.0–8.0), and Tris–HCl (pH 7.0–10.0) in a total volume of 180 μL at 30°C for 2 h. After the optimal pH was determined, the activities of the recombinant enzymes at various temperatures (0–60°C) were investigated in the optimal buffer [50 mM Tris–HCl buffer (pH 8.0)] for 2 h. The effects of the metal ions/chelating reagents (5 mM) on the activity of the recombinant enzymes were further investigated at the optimal pH [50 mM Tris–HCl buffer (pH 8.0)] and temperature (30°C). The enzyme was preincubated in 50 mM Tris–HCl buffer (pH 8.0) for 0–24 h at a temperature at 0 to 60°C to determine its thermostability, and the residual degradation activity was determined at 30°C for 2 h. The enzyme activity was estimated by measuring the absorbance at 232 nm. All reactions were performed in triplicate. The error bars represent the means of triplicates and are presented as the means±S.D.s.

### Activities of Aly35, Aly35-CD1, and Aly35-CD2 toward polysaccharide substrates

The enzyme activities were measured according to the method described by Gacesa (1992). Briefly, Aly35 was individually added to 1 mg/mL sodium alginate, PM or PG in 50 mM Tris–HCl buffer (pH 8.0) in a total volume of 1 mL. The reaction mixture was incubated at 30°C. At various time intervals (up to 10 min), aliquots of 100 μL were withdrawn in duplicate, boiled for 5 min, and then ice-cooled for 5 min. After centrifugation at 15,000 × g for 10 min, the supernatant was collected, diluted to 200 μL, and analyzed for the absorbance at 232 nm. One unit (U) was defined as the amount of enzyme required to increase the absorbance at 232 nm by 0.1 per min. The enzyme activities of Aly35-CD1 and Aly35-CD2 were determined as described above.

Moreover, the *K*_m_ values for Aly35, Aly35-CD1, and Aly35-CD2 toward sodium alginate, PM and PG were determined by nonlinear analysis based on the initial rates determined with 0.25–10 mg/mL of each substrate at 30°C.

### Gel filtration chromatography

Samples digested by the above recombinant enzymes were analyzed by gel filtration chromatography on a Superdex 30 Increase 10/300 GL column. The mobile phase was 0.20 M NH_4_HCO_3_ at a flow rate of 0.4 mL/min, and the eluted fractions were monitored at 232 nm using a UV detector. Online monitoring and data analysis (e.g., molar ratio determination) were performed using the software LC solution version 1.25.

### Digestion pattern of polysaccharides by Aly35, Aly35-CD1, and Aly35-CD2

To determine the polysaccharides digestion pattern by Aly35, the degradation products of sodium alginate (1 mg/mL), PM and PG, which were digested by Aly35 (2 μg), were traced at 30°C for 72 h with the addition of fresh enzyme every 24 h. Aliquots of the products (20 μg) were removed at different times and loaded onto a Superdex 30 Increase 10/300 GL column for analysis by monitoring at 232 nm.

To further determine the molecular weights and structural properties of the final oligosaccharide products of the three substrates, 10 mL (1 mg/mL) of sodium alginate was exhaustively digested by Aly35 at optimal conditions for 72 h with excess enzymes. The unsaturated disaccharide, unsaturated trisaccharide and unsaturated tetrasaccharide fractions were purified from the final alginate digests by enzyme and further identified by MS on an ion trap TOF hybrid mass spectrometer (LCMS-IT-TOF, Shimadzu, Japan). The electrospray ionization MS analysis was performed in negative ion mode. The mass acquisition range was set at 0–1000.

### Structural identity of the final products of Aly35

To determine the structures of each size-defined final oligosaccharide product, the signals of the unsaturated disaccharide (UDP2) and trisaccharide (UDP3) fractions were assigned from the one-dimensional proton NMR spectra referenced at 30°C. Each purified oligosaccharide fraction (2 mg) was dissolved in 0.5 mL of D_2_O in 5-mm NMR tubes. The spectra were recorded on a JNM-ECP600 (JEOL, Japan) apparatus set at 600 MHz.

### Analysis of the oligosaccharide-degrading properties of Aly35, Aly35-CD1, and Aly35-CD2

To determine the degradation properties of unsaturated oligosaccharides by Aly35 and truncated proteins, the partially digested products (such as UDP4 and UDP5) by Aly35 were fractionated as described previously. The oligosaccharide fraction (~20 μg) was digested with the above enzymes at 30°C. All the above oligosaccharides products were analyzed by HPLC using a Superdex 30 Increase 10/300 GL column with monitoring at 232 nm.

## Results

### Alginate lyase gene and protein sequence

**Figure 1:**
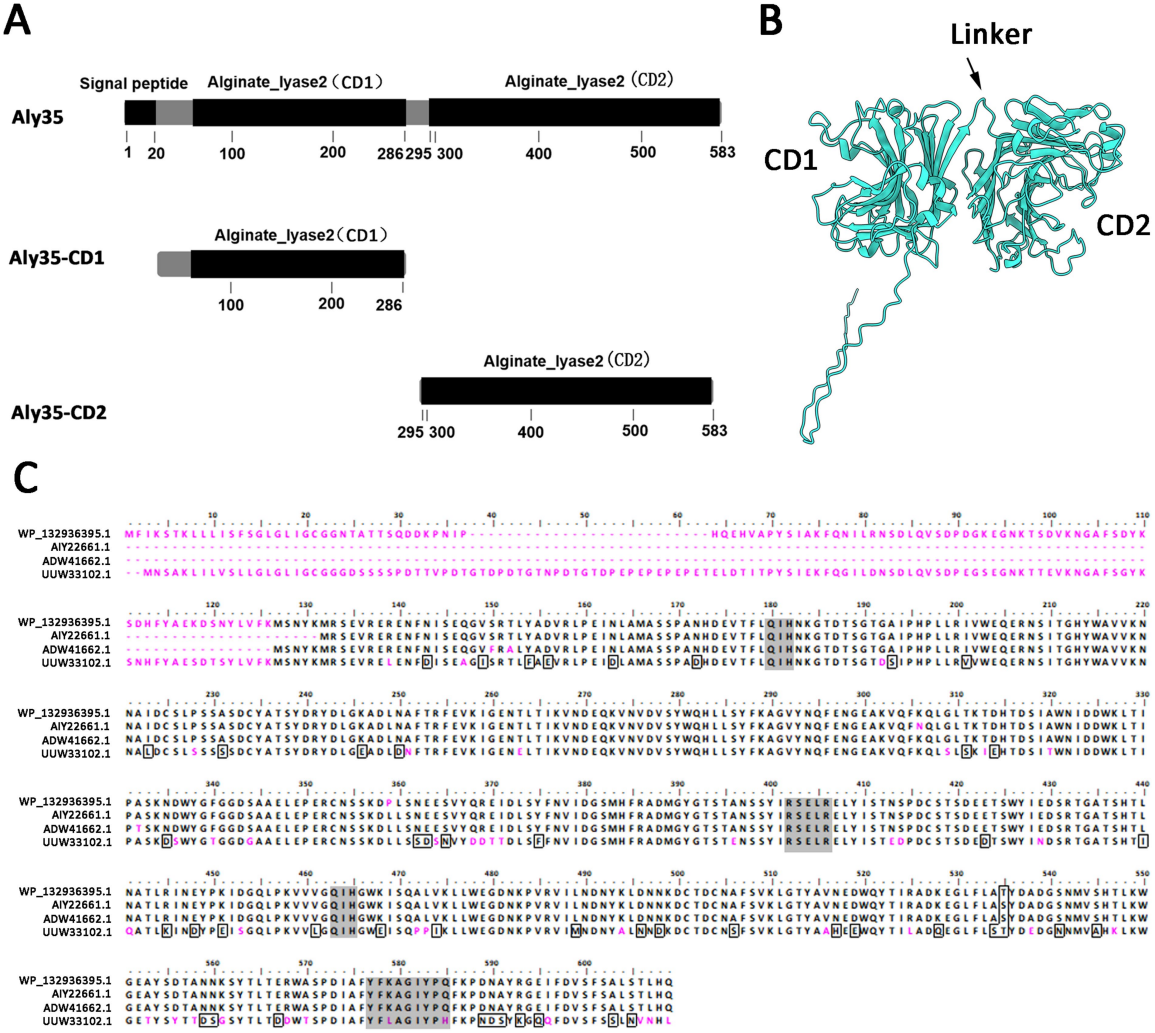
Module organization type of the alginate lyase Aly35 from *Vibrio alginolyticus* **(A)** module organization of the alginate lyase Aly35. It contains a putative N-terminal signal peptide (Met^1^-Gly^20^), two putative catalytic module of alginate_lyase2 superfamily: CD1 (Leu^53^-Lys^286^) and CD2 (Trp^295^-His^582^). The full-length protein was expressed to yield the recombinant protein Aly35, the alginate_lyase2 superfamily (Trp^295^-His^582^) was gene-deleted to obtain the truncated protein Aly35-CD1, the alginate_lyase2 superfamily (Leu^53^-Lys^286^) was gene-deleted to obtain the truncated protein Aly35-CD2 **(B)** protein structure prediction with AlphaFold2. CD, catalytic domain **(C)** amino acid Multiple sequences alignment of Aly35 with other three characterized PL7 alginate lyases, AIY22661.1/Algb from *Vibrio* sp. W13, ADW41662.1/lyase from *Vibrio midae*, UUW33102.1/AlgB from *Vibrio* sp.Ni1. Amino acid residues with homology 75% are outlined, and the conserved motifs in PL7 family were shaded in gray back.

The putative alginate lyase gene named *aly35* is 1,752 bp in length and has a GC content of 42%. The predicted full-length alginate lyase (Aly35) protein is composed of 583 amino acid residues and has a theoretical protein molecular weight of 65.68 kDa. The predicted isoelectric point (pI) is 5.12. SignalP 4.1 and LipoP 1.0 analyses indicated that Aly35 contains a type I signal peptide which composed with 20 amino acid residues (SP, Met^1^-Gly^20^) at its N-terminus. Simple Modular Architecture Research Tool analyses revealed that the Aly35 protein contains two alginate_lyase2 superfamily domains (CD1, Leu^53^-Lys^286^ and CD2, Trp^295^-His^582^) (Figure 1A). And the modeled structure also suggest Aly35 contain two domains, an N-terminal CD1 domain (Leu^53^-Lys^286^), a C-terminal CD2 domain (Trp^295^-His^582^) and a linker between CD1 and CD2 domains (Thr^287^-Ala^294^) (Figure 1B). Phylogenetic analysis showed that Aly35 (WP_132936395.1) has the closest evolutionary distance with polysaccharide lyase family 7 protein (HCZ9051391.1) from *Vibrio alginolyticus* (Supplementary Figure S1), suggesting Aly 35 belongs to the PL7 family.

BLASTp searches revealed that among the elucidated alginate lyases, the full-length sequence shares the highest sequence identity (99%, but 81% coverage) with a endo-type alginate lyase Algb (NCBI accession number AIY22661.1) from *Vibrio* sp. W13 (Zhu et al., 2015), which only have 495 amino acdis, and the catalytic domains were not studied. Aly35 shares sequence identity (80.96%) with an endo-type alginate lyase alg4755 (NCBI accession number UUW33102.1) from *Vibrio* sp. Ni1, but the putative two catalytic domains were not studied (Shu et al., 2023). The protein sequence alignment results also showed that the alginate_lyase2 superfamily module contains the conserved motifs QIH (Gln^180^-Ile^181^-His^182^, Gln^463^-Ile^464^-His^465^), RXEXR (R^402^-X^403^-E^404^-X^405^-R^406^) and YXKAGXYXQ (Y^577^-X^578^-K^579^-A^580^-G^581^-X^582^-Y^583^-X^584^-Q^585^, X represents any amino acid), which are conserved in elucidated PL7 alginate lyases (Figure 1C) to constitute the active center and are crucial for substrate recognition and catalysis (Yamasaki et al., 2004; Kawamoto et al., 2006).

### Heterologous overexpression of recombinant alginate lyases in *Escherichia coli*

In this study, we expressed the gene ORF3535, named *Aly35*, the base sequence is same as (GenBank accession number CP054701.1, from 1,512,571 to 1,514,322, *alginolyticus* strain GS_MYPK1 chromosome 2 gene). The full-length sequence and the truncated sequence of the alginate lyase ORF were amplified directly from the genomic DNA of *Vibrio* sp. strain H204 using PCR method, and the three PCR products were cut by restriction enzymes (Table 1). A His_6_ tag was successfully added to the N-terminus of the recombinant protein expression vector. Sodium dodecyl sulfate–polyacrylamide gel electrophoresis (SDS–PAGE) results indicated that *E. coli* BL21 (DE3) cells harboring the recombinant lyases plasmids could form soluble products with the correct molecular mass.

**Figure 2:**
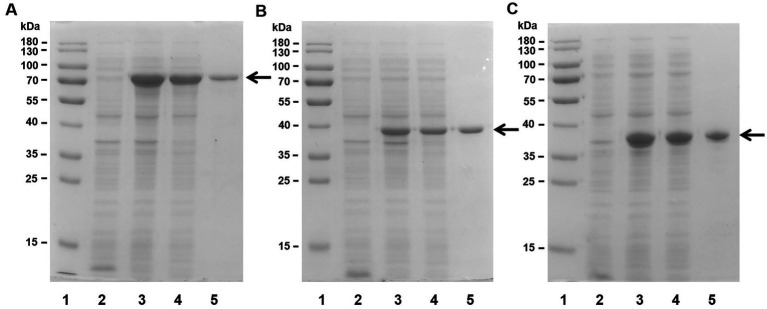
Purification of recombinant Aly35 **(A)** Aly35-CD1 **(B)**, and Aly35-CD2 **(C)** from *E. coli* by Ni^2+^ chelation chromatography. Enzyme purity following each fractionation step was assessed by SDS-PAGE using 12% (w/v) polyacrylamide gels, followed by staining with Coomassie Brilliant Blue. Lane 1, stained protein molecular weight marker PageRuler; lane 2, induced cell lysate of *E. coli* strains harboring the control plasmid pET-30a (+); lane 3, induced cell lysate of *E. coli* cells containing plasmid of pE30-Aly35 **(A)**, pE30-Aly35-CD1 **(B)** or pE30-Aly35-CD2 **(C)**; lane 4, supernatant fluid of the induced cell lysate; lane 5, Aly35 **(A)**, Aly35-CD1 **(B)** or Aly35-CD2 **(C)** protein purified from supernatant.

After collection of the host cells, the crude enzymes were extracted by sonication and centrifugation. Then, the recombinant enzymes were further enriched and purified by Ni-NTA affinity chromatography. SDS–PAGE revealed that the Aly35 (Figure 2A), Aly35-CD1 (Figure 2B), and Aly35-CD2 (Figure 2C) enzymes were eluted from the Ni-NTA column after a gradient of imidazole concentrations ranging from 50 to 250 mM was applied. The purified proteins had protein molecular masses consistent with the theoretical values and purities >90%.

### Biochemical characteristics of recombinant enzymes

**Figure 3:**
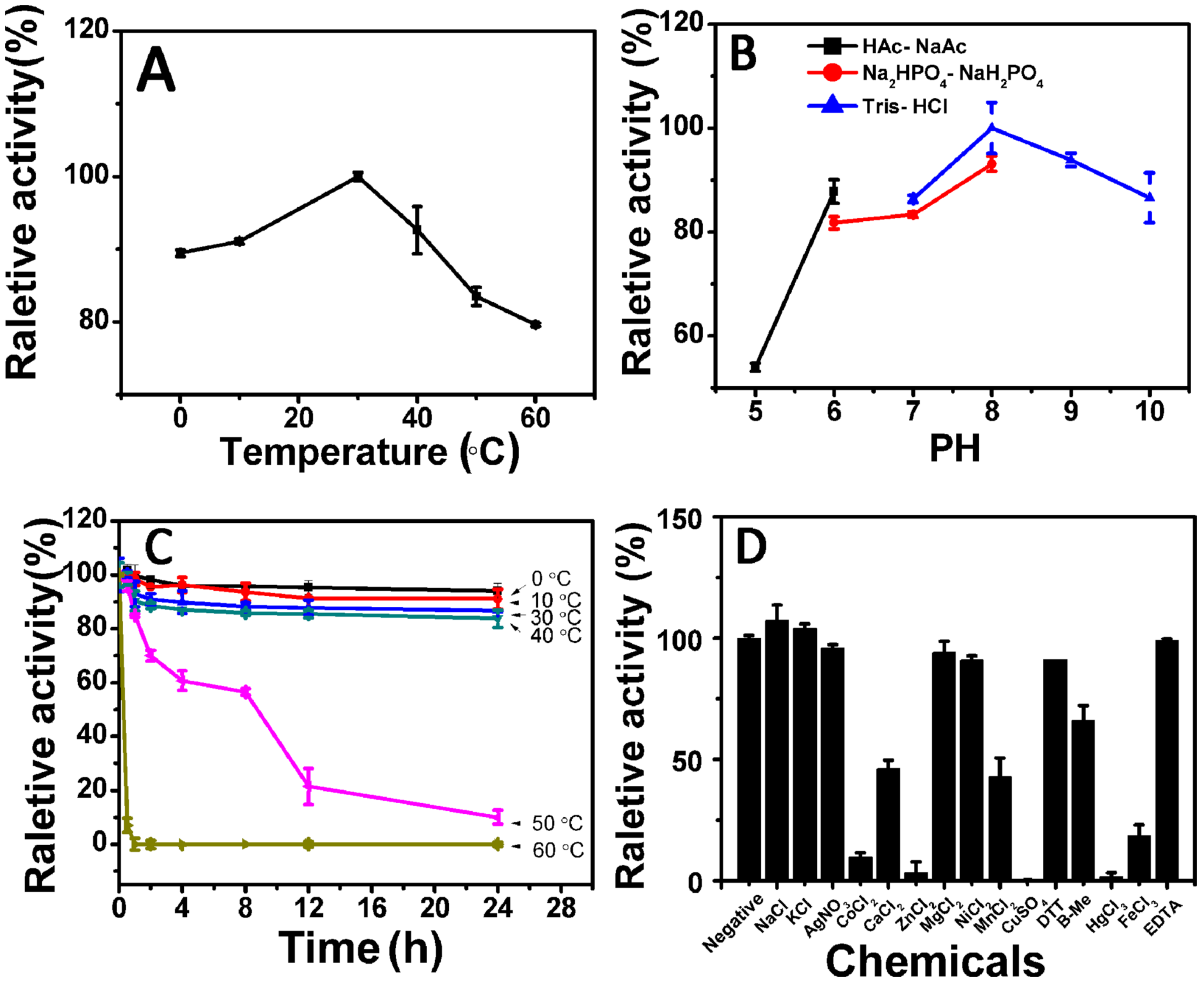
Biochemical characteristics of the recombinant enzyme Aly35. **(A)** Effects of temperature. The enzyme activities of Aly35 were measured using sodium alginate as substrate in the 50 mM Tris–HCl buffer (pH 8.0) at different temperatures for 2 h. Data are shown as the percentage of the activity of that obtained at 30°C (100%) for Aly35. **(B)** Effects of pH values. The enzyme activities of Aly35 against sodium alginate was measured in buffers with varying pH values from 5 to 10 at 30°C for 2 h. Data are shown as the percentage of the activity of that obtained in the 50 mM Tris–HCl buffer at pH 8.0 (100%). **(C)** Thermostability of Aly35. The enzyme in 50 mM Tris–HCl buffer (pH 8.0) was preincubated for 0 to 24 h under temperatures ranging from 0 to 60°C, and the residual activity against sodium alginate was estimated at 30°C. Data are shown as the activity relative to that of untreated Aly35. **(D)** Effects of metal ions. The enzyme activities of Aly35 against sodium alginate was measured in the Tris–HCl buffer (pH 8.0) containing a 5 mM concentration of various metal ions at 30°C for 2 h. Data are shown as the percentage of the activity of that obtained in the buffer without tested metal ions. The data showed that the enzymes is stable at 0 to 30°C. Error bars represent means of triplicates ± S.D.

**Figure 4:**
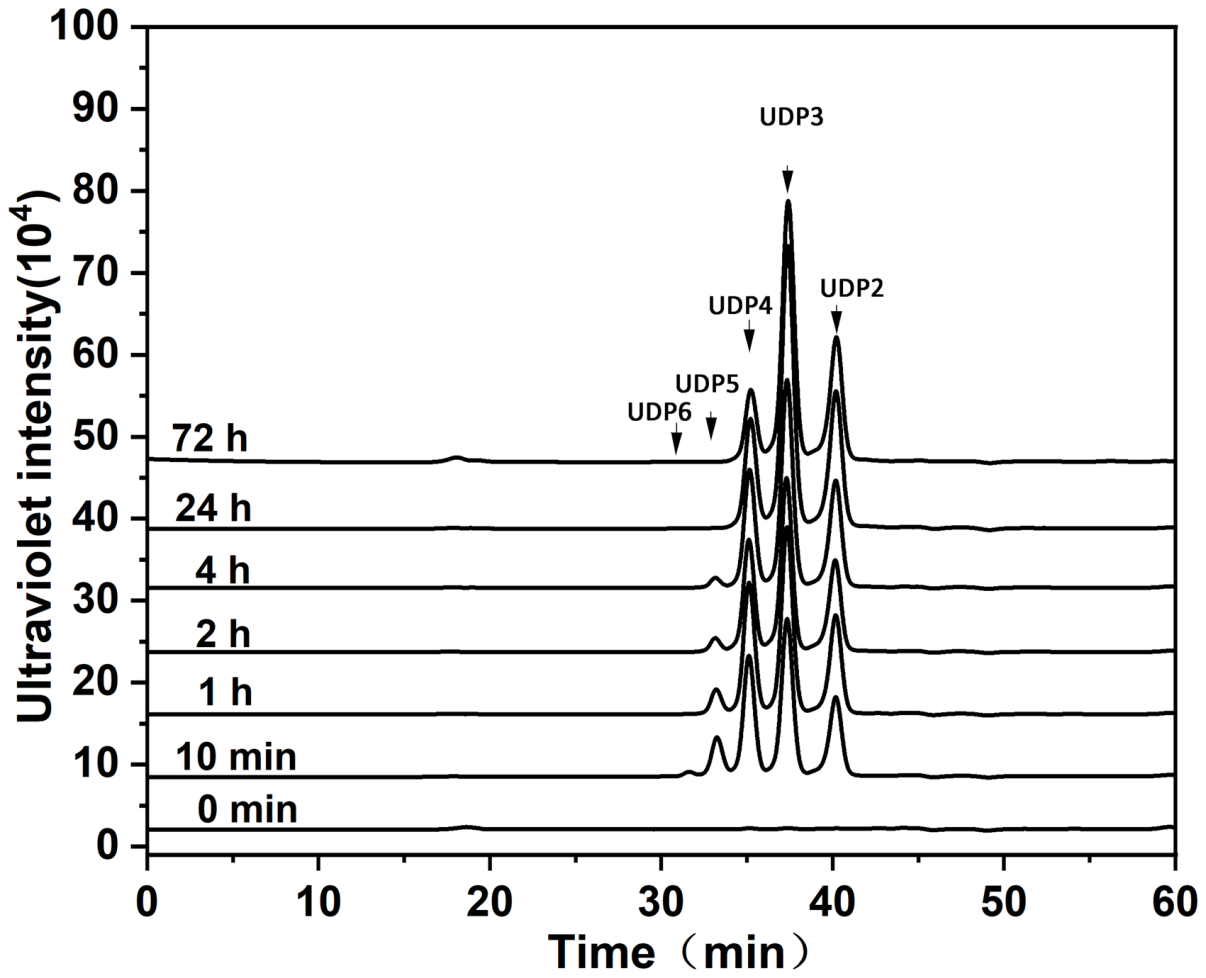
Degradation patterns of Aly35 toward sodium alginate. Sodium alginate (1 mg/mL) was treated with each enzyme (1 μg) at 30°C. A 20 μL aliquot was taken at different time points for gel filtration analysis as described in the Experimental Procedures. The elution positions of the unsaturated oligosaccharide product fractions with different degrees of polymerization are indicated by arrows: UDP2, unsaturated disaccharide; UDP3, unsaturated trisaccharide; UDP4, unsaturated tetrasaccharide; UDP5, unsaturated pentasaccharide; UDP6, unsaturated hexasaccharide.

**Figure 5:**
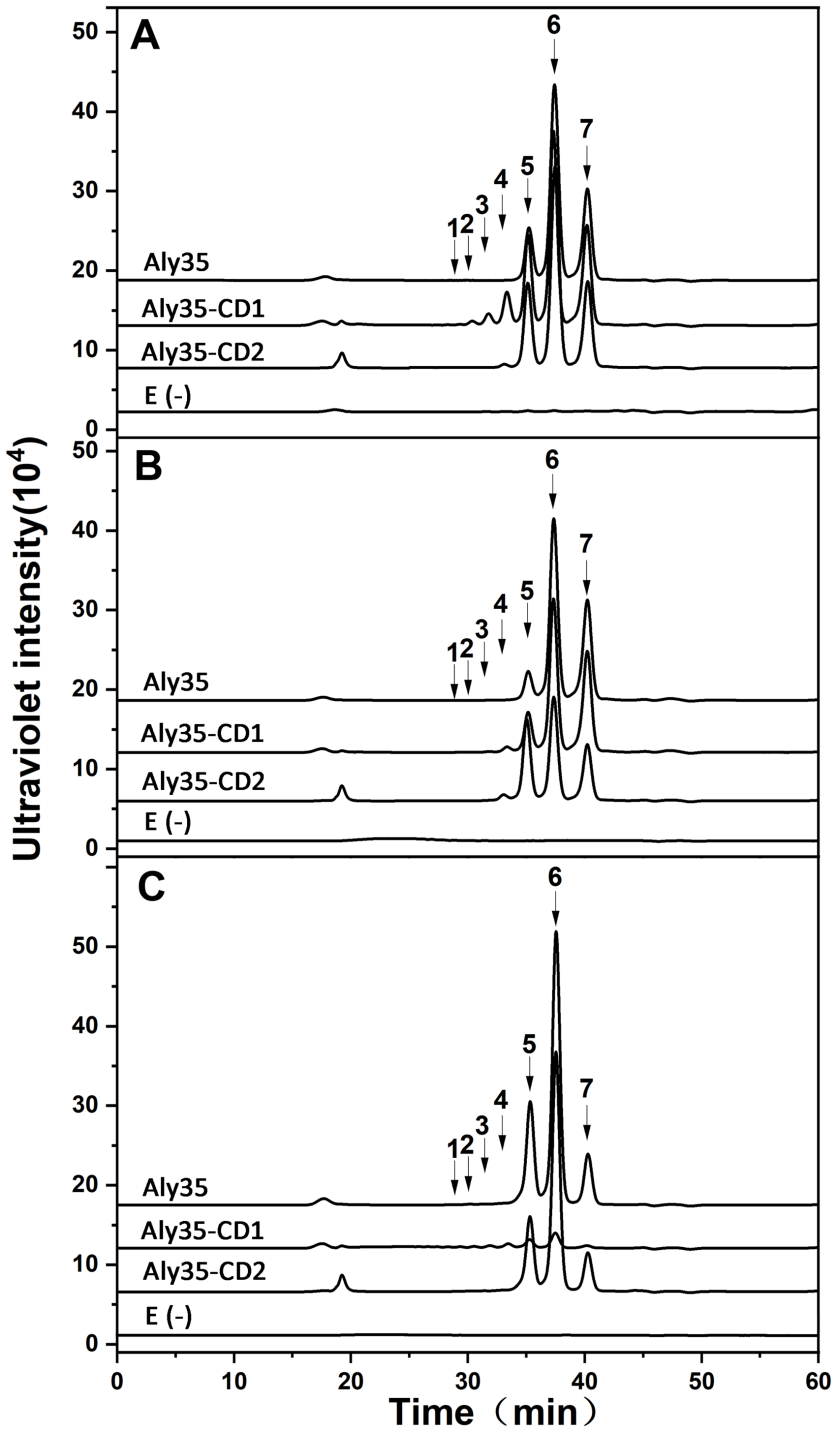
Analysis of the final digests of sodium alginate **(A)**, PM **(B)**, and PG(C) by Aly35, Aly35-CD1, and Aly35-CD2. Twenty micrograms of sodium alginate (ALG) **(A)**, PM **(B)**, or PG **(C)** was exhaustively digested with Aly35, and Aly35-CD1 and Aly35-CD2, separately, and then gel filtrated using a Superdex 30 Increase 10/300 GL column as described in the Experimental Procedures. E(−), controlling group without the enzyme. The elution positions of the unsaturated oligosaccharide product fractions with different degrees of polymerization are indicated by arrows: UDP2, unsaturated disaccharide; UDP3, unsaturated trisaccharide; UDP4, unsaturated tetrasaccharide; UDP5, unsaturated pentasaccharide; UDP6, unsaturated hexasaccharide; UDP7, unsaturated heptasaccharide; UDP8, unsaturated octasaccharide.

The protein Aly35 can degrade sodium alginate (Figures 3–5), PM and PG (Figure 5) to produce unsaturated oligosaccharides with a typical characteristic absorbance at 232 nm. The results indicated that the protein Aly35 is an alginate lyase that degrades sodium alginate and other associated substrates by a *β*-elimination mechanism.

The enzyme Aly35 performed optimally at a 30°C when sodium alginate was used as the substrate. The lyase activity remained at approximately 80% when the reaction temperature reached 60°C, indicating that the enzyme was insensitive to temperature (Figure 3A). The optimal pH was 8.0 in 50 mM Tris–HCl buffer, which was determined at 30°C (Figure 3B). The marine-derived alginate lyases often showed higher enzyme activity (more than 50% of the maximum activity) between 20 and 40°C and at pH 7.0–9.0, reflecting their ability to adapt to the marine environment (Badur et al., 2015; Zhu et al., 2015; Belik et al., 2020). Aly35 showed more than 80% of the maximum activity between 0 and 50°C and at pH 6.0–10.0 (Figures 3A,B). The above results suggested that the temperature and pH of Aly35 are wider than those of previously reported marine alginate lyase enzymes. The relative enzyme activity of Aly35 was approximately 85% when it was preincubated at 0–40°C for 24 h, suggesting that Half-time of the enzyme was more than 24 h (Figure 3C).

Different metal ion solutions with a final concentration of 5 mM were added to the buffer (50 mM Tris–HCl, pH 8.0) to determine their effects on the activity of Aly35. As shown in Figure 3D, the alginate-degrading activity of Aly35 was slightly promoted by the basic alkali Na^+^, and K^+^, slightly inhibited by Ag^+^, Ni^2+^, Ca^2+^, and Mn^2+^, and strongly inhibited by Co^2+^, Zn^2+^, Cu^2+^, Hg^2+^, Fe^3+^, moreover Mg^2+^ had no significant effects. Additionally, the reducing agent DTT, *β*-mercaptoethanol (*β*-Me), and chelating agent EDTA exhibited no significant simulative effect. Metal ions, especially divalent metal ions, were not necessary for the activity of Aly35 in this study. The effects of metal ions and reducing reagents on the enzyme activities of Aly35-CD1 and Aly35-CD2 were also investigated, and the results were similar to those obtained for Aly35 (Supplementary Figure S2).

### Analysis of recombinant enzyme activities

The specific activities of Aly35, Aly35-CD1, and Aly35-CD2 were measured under the optimal conditions of 50 mM Tris–HCl (pH 8.0) at 30°C as described in the Experimental Procedures. The results showed that the specific activities of Aly35 against sodium alginate, PM and PG were 3993.68 ± 215.26, 2213.96 ± 150.37 and 2394.07 ± 158.50 U/mg of protein, respectively. The specific activities of Aly35-CD1 against sodium alginate and PG were 2114.82 ± 139.78 and 2503.76 ± 155.91 U/mg of protein, respectively, but Aly35-CD1 did not significantly degrade PG. The specific activities of Aly35-CD2 against sodium alginate, PM and PG were 5784.98 ± 300.34, 1248.19 ± 59.45, and 5300.72 ± 260.18 U/mg of protein, respectively (Table 2).

**Table 2:** Specific activities and kinetic parameters of Aly35, Aly35-CD1, and Aly35-CD2 toward sodium alginate, PM and PG.

	Aly35			Aly35-CD1			Aly35-CD2		
	Sodium alginate	PM	PG	Sodium alginate	PM	PG	Sodium alginate	PM	PG
Specific activity (units/mg)	3993.68 ± 215.26	2213.96 ± 150.37	2394.07 ± 158.50	2114.82 ± 139.78	2503.76 ± 155.91	<100	5784.98 ± 300.34	1248.19 ± 59.45	5300.72 ± 260.18
*K*_m_ (mg/mL)	0.21 ± 0.02	2.84 ± 0.32	0.71 ± 0.04	0.22 ± 0.02	0.76 ± 0.05	1.224 ± 0.11	0.57 ± 0.03	3.80 ± 0.57	1.23 ± 0.15

Interestingly, the truncated protein Aly35-CD1 cannot effectively cut PG, effectively but it can easily degrade sodium alginate and PM. In addition, Aly35-CD2 had the highest PG-degrading activity. The above results also suggested that CD2 (Trp^295^-His^582^) was necessary for PG degradation activity and that the CD1 (Leu^53^-Lys^286^) has strong PM degradation ability compared to CD2. Furthermore, both Aly35 and its truncated proteins have the highest affinity for sodium alginate, as indicated by their low *K*_m_ values for sodium alginate (Table 2). And the kinetic plots are shown in Supplementary Figure S3.

Furthermore, to determine the difference between Aly35 and other alginate lyases which contain two catalytic domains, specific activity, Km, temperature optimal, pH optimal and thermostability comparisons between Aly35 and other lyases (AlyC6’ and AlyC8) were analyzed in Table 3. The results showed that Aly35 and its truncated proteins have lower Km value, but have longer half-life than those of AlyC6’ and AlyC8. The temperature optimal, pH optimal of three lyases are similar.

**Table 3:** Comparation between Aly35 and other alginate lyases which contain two catalytic domains toward sodium alginate.

Enzyme	Specific activity (U/mg)	V_max_ (U/mg)	K_m_ (mg/mL)	Temperature Optimal (°C)	pH optimal	Half-life (30°C, hour)
Aly35	3993.68 ± 215.26	ND	0.21 ± 0.02	30	8	> 24
Aly35-CD1	2114.82 ± 139.78	ND	0.22 ± 0.02	30	8	> 24
Aly35-CD2	5784.98 ± 300.34	ND	0.57 ± 0.03	30	8	> 24
AlyC6’	ND	7793.94 ± 235.61	2.12 ± 0.17	30	8	ND
AlyC6’-CD1	ND	4664.05 ± 253.98	2.09 ± 0.17	30	8	ND
AlyC6’-CD2	ND	594.39 ± 12.42	0.51 ± 0.02	30	8	ND
AlyC8	ND	ND	ND	30	8	7
AlyC8-CD1	ND	24415.9 ± 1974.3	1.64 ± 0.24	30	8	7
AlyC8-CD2	ND	2270.6 ± 160.2	3.43 ± 0.52	30	8	7

ND, not determined.

### Degradation pattern of polysaccharides by Aly35, Aly35-CD1, and Aly35-CD2

To determine the action patterns of Aly35, the degradation of sodium alginate (1 mg/mL) by Aly35 (1 μg) was monitored at 30°C. The reaction time intervals were 0, 10 min, and 1, 2, 4, 12, 24 and 72 h. The products were filtered and loaded onto a Superdex 30 Increase 10/300 GL column and monitored at 232 nm. Aly35 initially produced higher molecular mass oligosaccharides and then smaller oligomers with strong absorbance at 232 nm (Figure 4), again suggesting that the Aly35 is a lyase and that its catalytic domain (alginate_lyase2 superfamily) of Aly35 degrades sodium alginate polysaccharides via an endolytic pattern mechanism. Moreover, the final products of Aly35 were mainly UDP2, UDP3, and UDP4 (Figure 4).

Furthermore, sodium alginate (Figure 5A), PM (Figure 5B), and PG (Figure 5C) were exhaustively digested with Aly35, Aly35-CD1, and Aly35-CD2, respectively, at 30°C for 72 h to determine the final oligosaccharide products. The above oligosaccharide products were also analyzed by gel filtration analysis using authentic unsaturated oligosaccharides derived from sodium alginate, PM and PG. As shown in Figure 5, based on the retention times reported previously, the UDP4, UDP3 and UDP2 fractions were the final main products of sodium alginate when digested with Aly35 and Aly35-CD2, while the UDP8, UDP7, …, and UDP2 fractions were the final main products of sodium alginate when digested by Aly35-CD1 (Figure 5A). The UDP4, UDP3 and UDP2 fractions were the final main products of PM after digestion with Aly35, Aly35-CD1, and Aly35-CD2, respectively (Figure 5B). The UDP4, UDP3 and UDP2 fractions were the final main products of PG after digestion with Aly35 and Aly35-CD2. However, only a small amount of oligosaccharide was detected in the digests of PG digested with Aly35-CD1 (Figure 5C). Aly35-CD1 can better degrade PM than sodium alginate, and Aly35-CD2 has the highest PG-degrading activity (Table 2) thus, the complementary substrate specificities of Aly35-CD1 and Aly35-CD2 enable the full-length enzyme to efficiently degrade all types of glycosidic bonds in alginate.

Regardless of which substrate is degraded by Aly 35 and its truncated proteins, UDP3 has a larger peak area (i.e., the highest molar content) than that of other oligosaccharide products; however, UDP2 is the main product of Alg4755 from *Vibrio* sp. Ni1, although the sequence identity of the two enzymes reached 80.96% (Shu et al., 2023).

### Identification of the final main oligosaccharide products from sodium alginate

**Figure 6:**
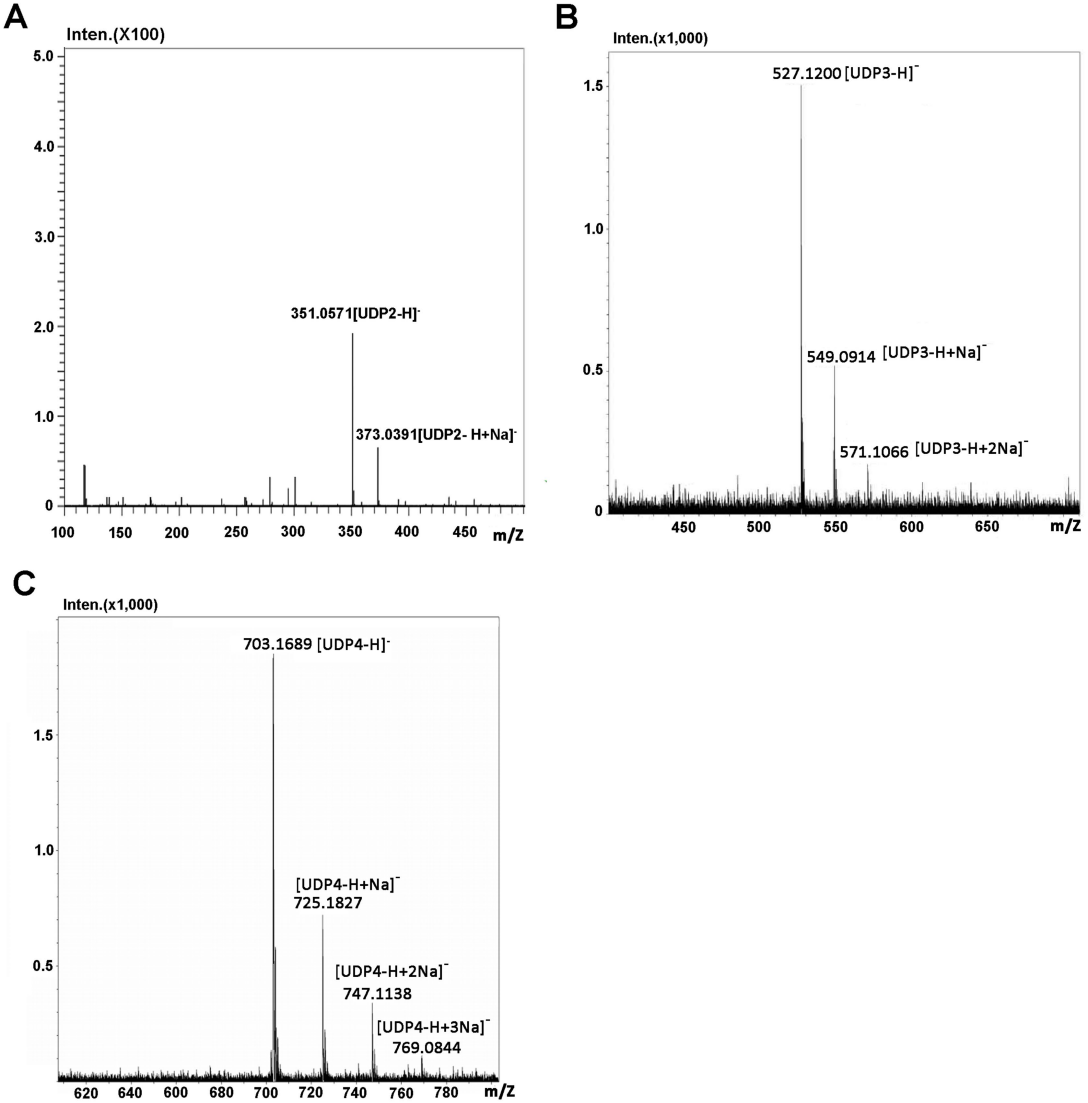
Time-of-flight mass spectra of the final oligosaccharide products of sodium alginate digested by Aly35. The main final products obtained from sodium alginate digested each of enzymes were identified by electrospray ionization MS on anion trap TOF hybrid mass spectrometer as described under “Experimental Procedures.” The products are indicated as: UDP3 produced by Aly35 **(A)**; UDP4 produced by Aly35 **(B)**; UDP5 produced by Aly35 **(C)**.

The oligosaccharide products of sodium alginate (10 mg) digestion by Aly35 were separated by size exclusion chromatography as described in the Experimental Procedures. The m/z values of 351.0571 [UDP2-H]^−^ was consistent with the theoretical molecular masses of unsaturated disaccharides (Figure 6A), 527.1200 [UDP3-H]^−^ was consistent with the theoretical molecular masses of unsaturated trisaccharides (Figure 6B), and 703.1689 [UDP4-H]^−^ was consistent with the theoretical molecular masses of unsaturated tetrasaccharides (Figure 6C) in the MS spectrum. The results demonstrated that the UDP2, UDP3 and UDP4 fragments constitute the main oligosaccharide products of Aly35 and suggested that Aly35 is a lyase that produces unsaturated oligosaccharides.

**Figure 7:**
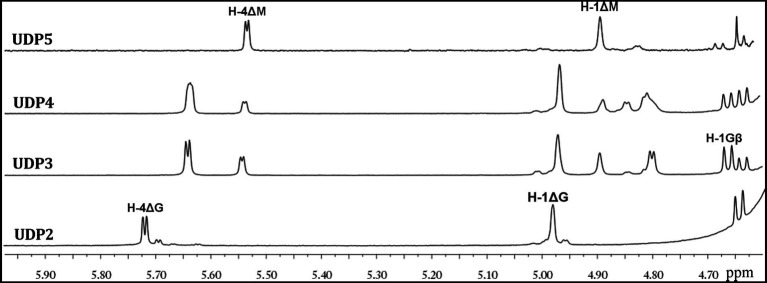
^1^H NMR (600 MHz, 28°C) spectra of the final main oligosaccharide product fractions of sodium alginate by Aly35. Each size-defined oligosaccharide product fractions produced by the two enzymes were individually purified using a Superdex 30 Increase 10/300 GL column, monitored at 232 nm. H-4 *Δ* doublet at 5.71 ppm or 5.67 ppm indicated that the neighbor to Δ is a G residue, meaning that ∆G constitutes the first two sugar residues at the nr ends. The chemical shift of the H-4 Δ signal at 5.56 ppm means that ΔM constitutes the first two sugar residues at the nr ends. As shown in the figure, the intensity of the H-4 ΔG signal is higher than that of H-4 ΔM for trisaccharide products, the intensity of the H-4 ΔM signal is higher than that of H-4 ΔG for UDP4 fraction, demonstrating that the final UDP4 product fractions contain ΔM ends at greater proportions than ΔG ends.

**Figure 8:**
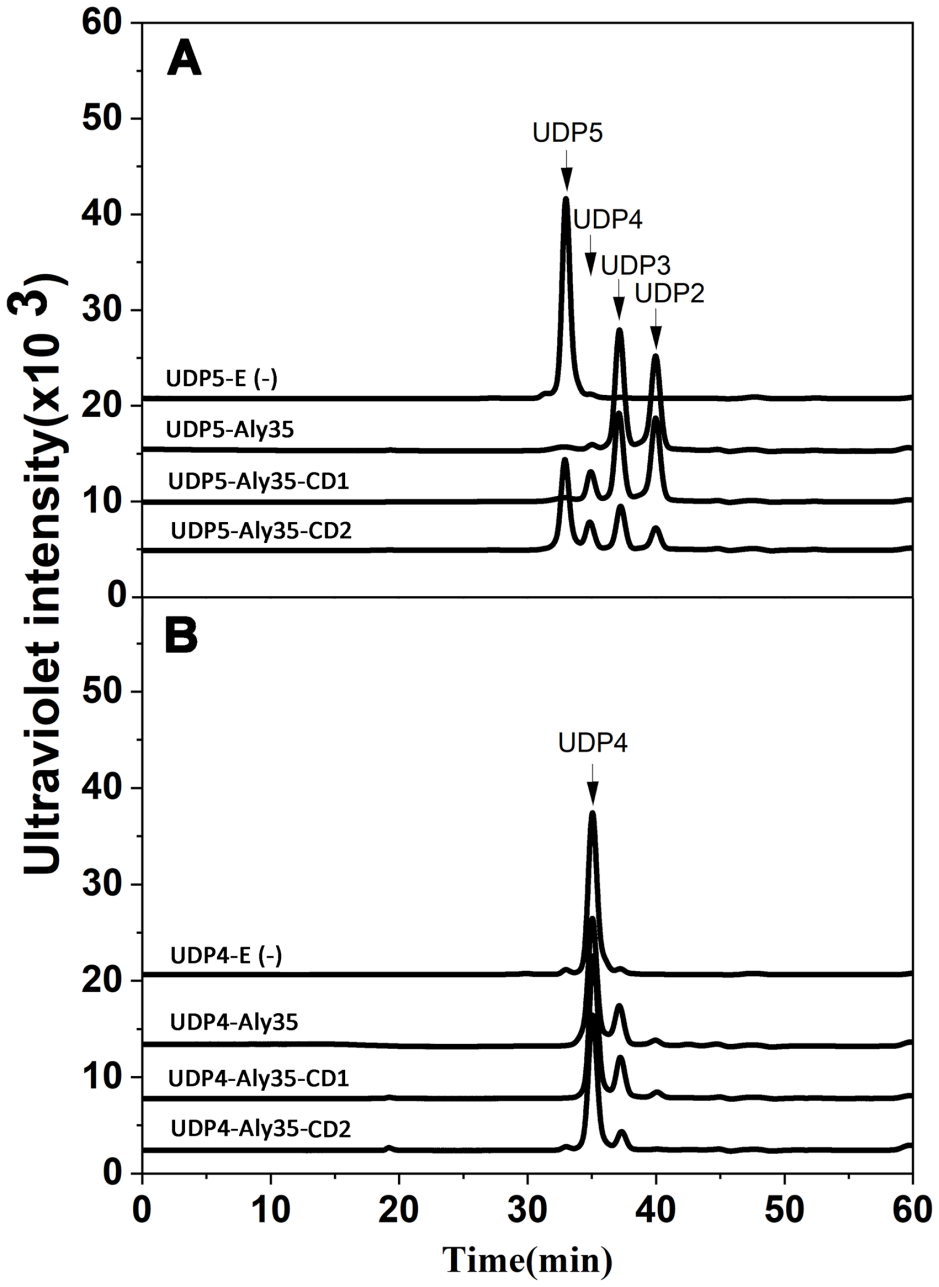
Degradation patterns of Aly35 and its truncated protein Aly35-CD1 and Aly35-CD2 toward unsaturated fractions of pentasaccharide **(A)** and tetrasaccharide **(B)** degradation of ~20 μg UDP5 fractions **(A)** and degradation of ~20 μg UDP4 fractions **(B)** for 12 h by 1 μg three enzymes at 30°C individually. E (−), without the enzyme. HPLC analyses were performed using a Superdex 30 Increase 10/300 GL column monitored at 232 nm. The elution positions of the unsaturated oligosaccharide product fractions with different degrees of polymerization are indicated by arrows: UDP2, unsaturated disaccharide; UDP3, unsaturated trisaccharide; UDP4, unsaturated tetrasaccharide; UDP5, unsaturated pentasaccharide.

^1^H-NMR was then used to analyze the properties of the UDP2, UDP3, UDP4 and UDP5 fractions. The ^1^H-NMR chemical shifts were assigned according to previously published data for alginate oligosaccharides. The structure of the reducing end sugar can be easily identified by the characteristic chemical shift of its anomeric proton signal. The chemical shifts of the protons of unsaturated non-reducing ends are dependent on the nature of the nearest sugar residue, as identified from the shift in the H-4 signal of Δ. The strong H-4 ΔG signal at 5.72 ppm suggested that the UDP2 fractions mainly contained ΔG disaccharides, demonstrating that Aly35 mainly yielded ΔG as a disaccharide product when degrading the designated alginate substrate. A very minor signal was observed of H-4ΔM (Figure 7). The UDP3 and UDP4 fractions showed an H-4 (Δ) doublet at 5.67 ppm, indicating that the neighbor to the Δ unit was a G residue, while the chemical shift of the H-4 signal appearing at 5.56 ppm ndicated that its neighbor was an M residue. Furthermore, the residues at the reducing ends of alginate oligosaccharides can be determined according to the characteristic signals of their anomeric protons; this is because the *β*-anomeric protons of the G and M residues at the reducing ends have a characteristic doublet at 4.71 ppm with ^3^*J*_HH_ = 8.4 Hz and a single peak at 4.70–4.80 ppm (Thomas et al., 2013; Chen et al., 2018), respectively. Thus, the sequences of UDP3 from the final products can be directly determined using ^1^H-NMR spectroscopy, as shown in Figure 8. UDP3 mainly contains ΔGG and ΔMG. According to the ^1^H-NMR spectra shown in the picture, the structures of UDP4 can be preliminarily determined to be Δ GXG and ΔMXG, and UDP5 can be preliminarily determined to be ΔMXXG. Therefore, although Aly35 yielded large products containing ΔG ends, Aly35 preferred to produce small oligosaccharides in which the non-reducing ends primarily contained ΔG units. As a result, we speculate that the activity on PG is greater than that on PM, which is consistent with the results shown in Table 2.

### Pattern of oligosaccharide substrates degradation by Aly35, Aly35-CD1, and Aly35-CD2

To determine and compare the degradation patterns of the three enzymes toward oligosaccharide substrates, sodium alginate was initially partially digested by Aly35 as previously described. Subsequently, the intermediate product fractions of UDP2, UDP3, UDP4 and UDP5 were collected and pooled through gel filtration using a Superdex 30 Increase 10/300 GL column and finally characterized via MS analysis. After further enzymatic reactions with Aly35, Aly35-CD1 or Aly35-CD2, the fractions of the UDP5 (Figure 8A) and UDP4 (Figure 8B) fractions were analyzed by gel filtration HPLC as described above. These results showed that the Aly35, Aly35-CD1, and Aly35-CD2 could not degrade the UDP2, or UDP3 (Supplementary Figure S4) fractions to produce the UDP2 product. The three enzymes generally cannot degrade UDP4 (Figure 8B), and fewer tetrasaccharide products may be products of pentasaccharide impurities. When reacted with the UDP5 fraction, UDP2, and UDP3 products were the main products. A small amount of the tetrasaccharide product may result from the hexasaccharide impurity, not be the product from UDP5. Thus, the smallest substrate for Aly35 is the UDP5 fraction, and the minimal product of Aly35 is the unsaturated disaccharide.

## Discussion

Microorganisms can express a variety of alginate lyases, such as *Pseudomonas*, *Azotobacter*, *Photobacterium*, *Sphingomonas*, *Bacillus*, *Flammeovirga*, *Vibrio* genus and so on. Among the above microorganisms, several *Vibrio* strains can efficiently degrade alginate polysaccharides, moreover only two enzymes who contain two lyase catalytic domains up to now, both enzymes were from *Vibrio* genus (Zhu et al., 2015; Sha et al., 2022; Sun et al., 2022; Wang et al., 2022; Shu et al., 2023), indicating that they are abundant resources of various efficient alginate degrading enzymes.

In this study, the endo-lyase Aly35 was identified from a marine bacterium, *Vibrio* sp. H204. Aly35 protein belong to the PL7 family, is composed of 583 amino acid residues, and has the highest similarity (but 81% coverage) with Algb from *Vibrio* sp. W13. However Algb is composed of 495 amino acids, and its two lyase catalytic domains function were not studied and reported. Alginate lyases AlyC6’ from *Vibrio* sp. NC2 (Wang et al., 2022), and AlyC8 from *Vibrio* sp. C42 (Sun et al., 2022) were reported contain two lyase catalytic domains with synergistic effects at present. Here, Aly35 has two lyase catalytic domains, and shares the sequence identity with AlyC8 (67.84%), followed by AlyC6’(67.65%), while AlyC6’ shares the sequence identity (99.31%) with AlyC8. Besides, full-length Aly35 shares very low sequence identity (24.82%) with Aly2 that studied by our group from *Flammeovirga* sp. strain MY04 (Peng et al., 2018a,b). This suggest that aly35 maybe has different functions from AlyC6’, AlyC8, and Aly2.

To verify the above hypothesis, two lyase catalytic domains of Aly35 were also studied independently. The biochemical characteristics of Aly35, Aly35-CD1, and Aly35-CD2 was studied firstly, they showed wider temperature and pH adaptation (Figure 3), better thermal stability compared with other alginate lyase which has two catalytic domains. Then the enzyme activities were tested (Table 2), and the activities on different substrates, sodium alginate, polyM and polyG, were higher than that of Algb (Zhu et al., 2015). The enzyme activities of Aly35 and its truncated protein indicated that Aly35 is a bifunctional alginate lyase, as is the case for its truncated proteins Aly35-CD2; in addition, Aly35 exhibited the highest degrading activity to sodium alginate at same mole. The specific activities of Aly35 against sodium alginate and PG were greater than those of AlyC6’, and the PM-degrading activities were similar (Wang et al., 2022); moreover, Aly35 and Aly35-CD2 had greater PG-degrading activities than those of AlyC6’ and AlyC8 (Sun et al., 2022). At present, only one commercial alginate lyase (Sigma) with a specific activity greater than 10 U/mg is sold, but it is expensive and is sold in small quantities. It has been reported that the alginate lyase AlgA2 from *Flavobacterium* sp. can degrade alginate and produce a series of AOSs, but its enzyme activity is low (Huang et al., 2013).

The recombinational protein molecular weight of Aly35, Aly35-CD1, and Aly35-CD2 are of 69.03 kDa, 35.44 kDa and 38.19 kDa, respectively. Apparently, the molecular weight of aly35 is about 1.94 times that of Aly35-CD1, and 1.81 times that of Aly35-CD2. For the same mass, there are more molar of Aly35-CD1 and Aly35-CD2 than that of Aly35. The enzyme activity toward to alginate sodium of 0.02 mole Aly35, Aly35-CD1, and Aly35-CD2 were 5511.27 U, 1497.82 U, 4416.74 U, respectively; toward to PM of 0.02 mole Aly35, Aly35-CD1, and Aly35-CD2 were 3055.26 U, 1773.45 U, 952.97 U, respectively; toward to PG of 0.02 mole Aly35, Aly35-CD1, and Aly35-CD2 were 3303.81 U, <100 U, 3303.81 U, respectively. Aly 35 has the highest activity with equals molar equivalents toward sodium alginate, PM compared to Aly35-CD1and Aly35-CD2.

Aly35-CD1 can easily degrade sodium alginate and PM, but cannot effectively cut PG effectively. In addition, Aly35-CD2 had the highest PG-degrading activity. The CD1 has strong PM degradation ability compared to CD2, and CD2 was necessary for PG degradation activity. And Supplementary Table S2 showed that with equals molar equivalents, the complete enzyme Aly35, sodium alginate and PM activity is higher than in the Aly35-CD2 enzyme, and sodium alginate/ PM/PG activity are more higher. PG activity of Aly35 is lower than in the Aly35-CD2 enzyme indicated Aly35-CD2 prefer degrading PG. PM activity of Aly35-CD2 is lower than in the Aly 35 and Aly35-CD1. The above results and Figure 5 suggested only Aly35-CD2 can degrade alginate, PM, and PG completely, and Aly35-CD1 and Aly35-CD2 together degrade alginate more effectively.

The UDP4, UDP3 and UDP2 fractions were the main products of sodium alginate, PM and PG when digested with Aly35 and Aly35-CD2 (Figure 5). However, series oligosaccharides were products of sodium alginate when digested by Aly35-CD1 (Figure 5A). Moreover Aly-CD1 cannot degrade PG effectively since there are only a small amount of oligosaccharide was detected in the digests of PG digested with Aly35-CD1 (Figure 5C), and we speculate that it may be caused by impurity of PG. Interestingly, Aly35-CD1 can degrade PM very efficiently compared with Aly35 and Aly35-CD2 (Figure 5).

Aly35-CD2 has more higher specific activities when sodium alginate, PM, PG were substrates than AlyC6’-CD2 and AlyC8-CD2. AlyC8-CD1 has higher specific activity than AlyC8 and AlyC8-CD2 when sodium alginate was degraded, but that is not the case with Aly35 and AlyC6’. As for hydrolysates with polyM as substrate, UDP2 were the main products when degraded by Algb, but UDP3 were the main products when degraded by Aly35 (Figure 5B). Aly35 has a stronger affinity with the substrate than AlyC6’ and AlyC8 because of the low Km of Aly35, and has longer half-life at 30°C. In the present study, Aly35 exhibits high enzyme activity and stable biochemical characteristics, making it a highly valuable commercial tool.

In this study, a novel bifunctional endolytic alginate lyase named Aly35 with two alginate lyase domains from *Vibrio* sp. H204 was identified and characterized. The lyase Aly35 belongs to the PL7 family. The optimal temperature, solvent and pH of Aly35 were 30°C, and Tris–HCl, pH 8.0, respectively, and Aly35 showed higher thermal and pH stability than that of the reported alginate lyase. The specific activities and degradation products of Aly35 and its truncated proteins Aly35-CD1 and Aly35-CD2 were also analyzed. Furthermore, the two catalytic domains exert a synergistic degradation effect on alginate although Aly35-CD2 can cleave three substrates. In addition, Aly35-CD1 can be used to prepare G unit–rich oligosaccharides from alginate because it can degrade M-blocks but not G-blocks, Aly35 and Aly35-CD2 will be useful tools for performing structural analyses and preparing bioactive oligosaccharides. High enzyme activity, thermal and pH stability, and unique clear degradation patterns make them highly commercial valuable. The results provide extended insights into alginate lyase groups and are helpful for guiding the design of new enzymes for efficient alginate depolymerization.

## Data Availability

The original contributions presented in the study are included in the article/Supplementary material, further inquiries can be directed to the corresponding authors.

## Footnotes

^1^ http://www.cazy.org/

^2^ http://www.ncbi.nlm.nih.gov

^3^ http://swissmodel.ExPASy.org/

^4^ https://alphafoldserver.com/welcome

^5^ http://atgc.lirnm.fr/phyml

## Supplementary material

SUPPLEMENTARY FIGURE S1 The phylogenetic tree of alginate lyases Aly35. The phylogenetic tree of Aly35 constructed by phylogeny.fr online web service, through alignment of protein sequences by blast analysis and scoring of alignments between protein sequences was performed by BLOSUM62 matrix. The red colored vales are the branch support values obtained by 1000 replications through bootstrap method. The arrow indicates Aly35. All the protein used in the phylogenetic tree belong to PL7 family.

SUPPLEMENTARY FIGURE S2 Effects of metal ions on Aly35-CD1 and Aly35-CD2. The enzyme activities of Aly35-CD1 **(A)** and Aly35-CD2 **(B)** against sodium alginate was measured in the Tris-HCl buffer (pH 8.0) containing a 5 mM concentration of various metal ions at 30 °C for 2 h.

SUPPLEMENTARY FIGURE S3 Non-linear fit curves for the hydrolysis of three substrates: sodium alginate, PM, PG by Aly35 **(A)**, Aly35-CD1 **(B)**, Aly35-CD2 **(C)**. The initial rates were determined with 0–10 mg/mL of each substrate at 30 °C.

SUPPLEMENTARY FIGURE S4 Degradation patterns of Aly35 and its truncated proteins Aly35-CD1 and Aly35-CD2 toward unsaturated fractions of trisaccharide and disaccharide. Degradation of ~20 μg UDP3 fractions and degradation of ~20 μg UDP2 fractions for 12h by 1 μg three enzymes at 30 °C individually. E (−), without the enzyme. HPLC analyses were performed using a Superdex 30 Increase 10/300 GL column monitored at 232 nm.

SUPPLEMENTARY TABLE S1 The protein protein sequences were used to construct phylogenetic tree.
