# Supplementary figures and images for: A bifunctional endolytic alginate lyase with two different lyase catalytic domains from Vibrio sp. H204
Source: Front Microbiol. 2024 Dec 13;15:1509599. doi: 10.3389/fmicb.2024.1509599 (PMC11671496; doi:10.3389/fmicb.2024.1509599)

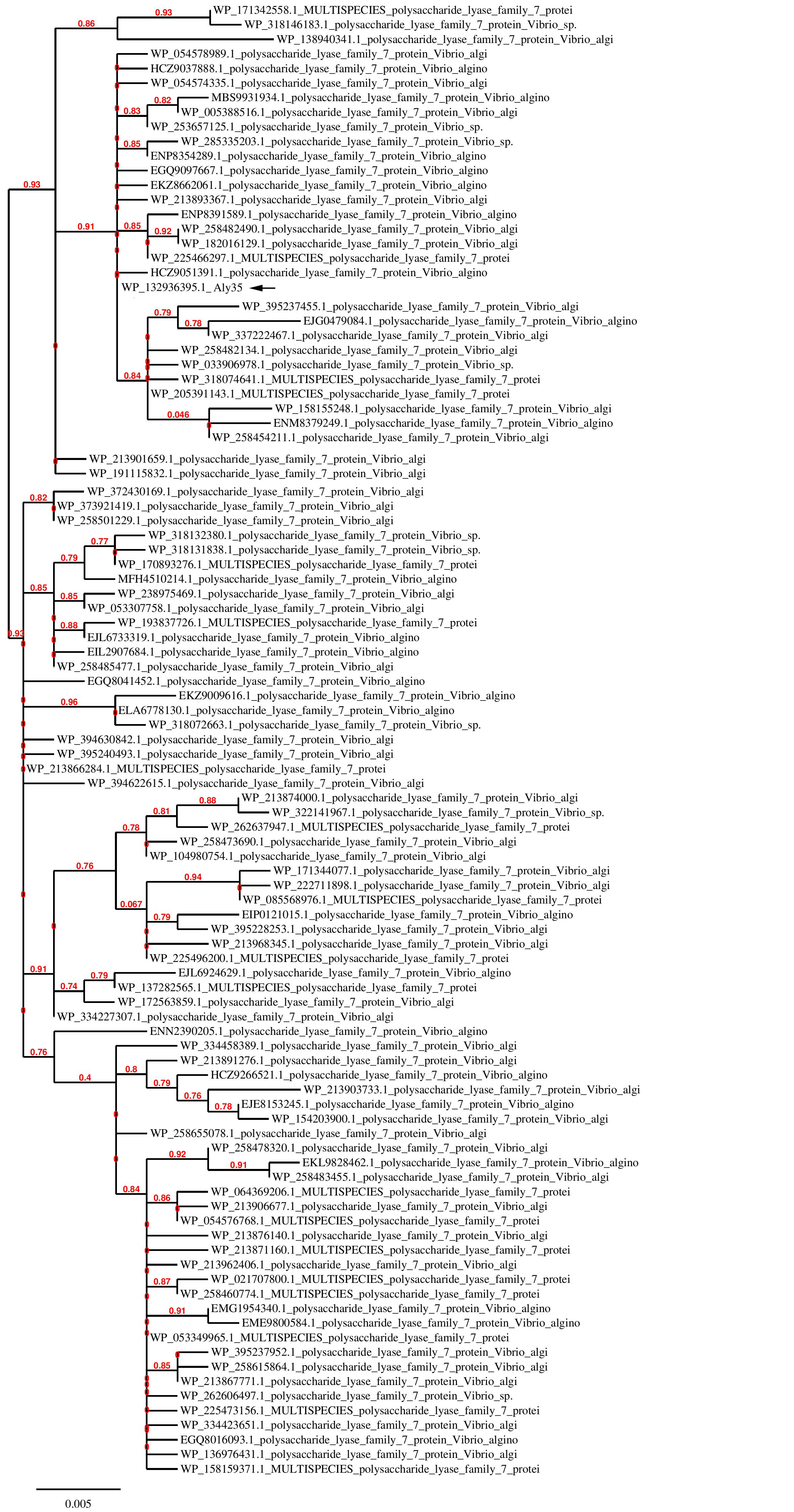

Supplement: SUPPLEMENTARY FIGURE S1 — The phylogenetic tree of alginate lyases Aly35. The phylogenetic tree of Aly35 constructed by phylogeny.fr online web service, through alignment of protein sequences by blast analysis and scoring of alignments between protein sequences was performed by BLOSUM62 matrix. The red colored vales are the branch support values obtained by 1000 replications through bootstrap method. The arrow indicates Aly35. All the protein used in the phylogenetic tree belong to PL7 family. [file Image_1.tif]

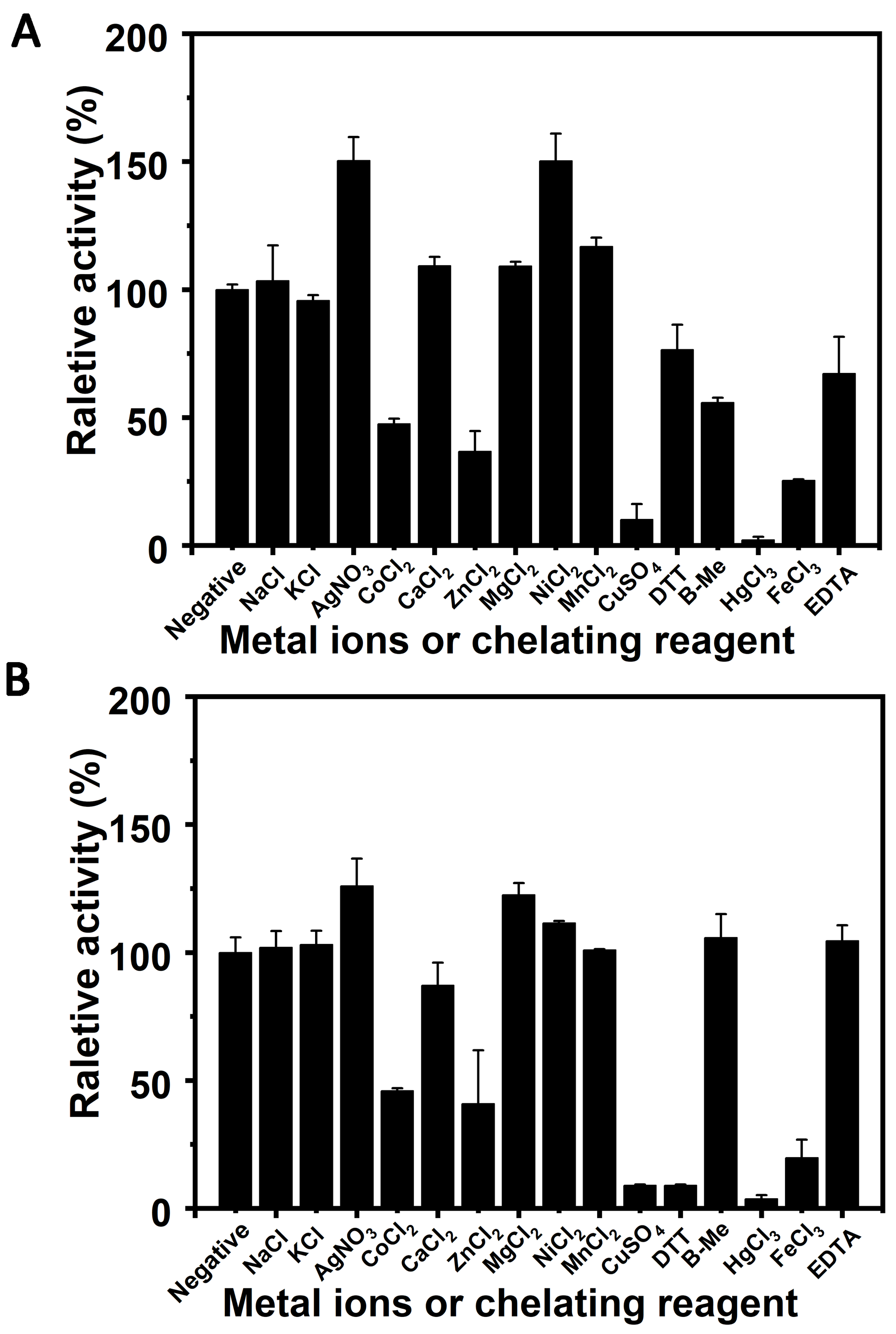

Supplement: SUPPLEMENTARY FIGURE S2 — Effects of metal ions on Aly35-CD1 and Aly35-CD2. The enzyme activities of Aly35-CD1 (A) and Aly35-CD2 (B) against sodium alginate was measured in the Tris-HCl buffer (pH 8.0) containing a 5 mM concentration of various metal ions at 30 °C for 2 h. [file Image_2.tif]

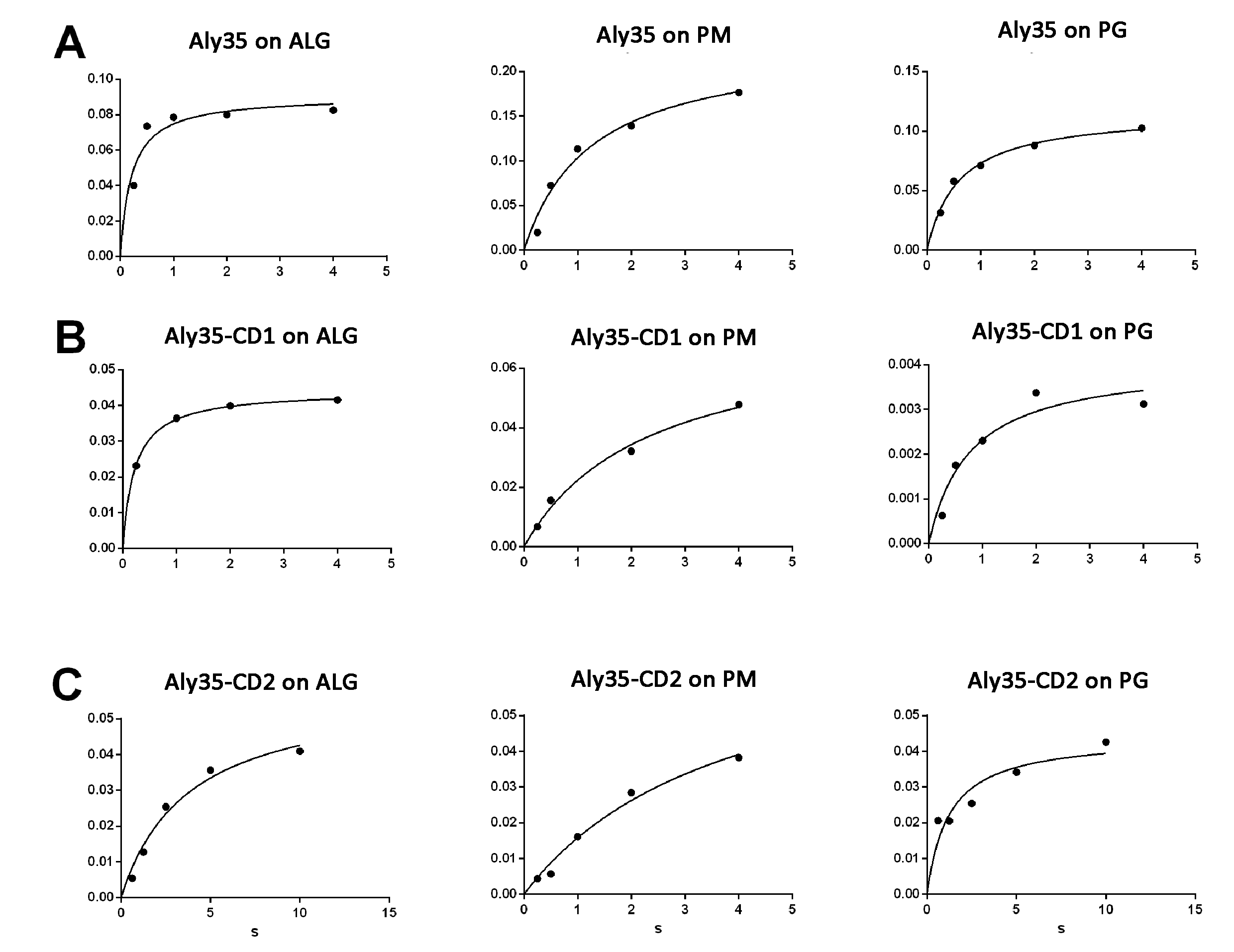

Supplement: SUPPLEMENTARY FIGURE S3 — Non-linear fit curves for the hydrolysis of three substrates: sodium alginate, PM, PG by Aly35 (A), Aly35-CD1 (B), Aly35-CD2 (C). The initial rates were determined with 0–10 mg/mL of each substrate at 30 °C. [file Image_3.tif]

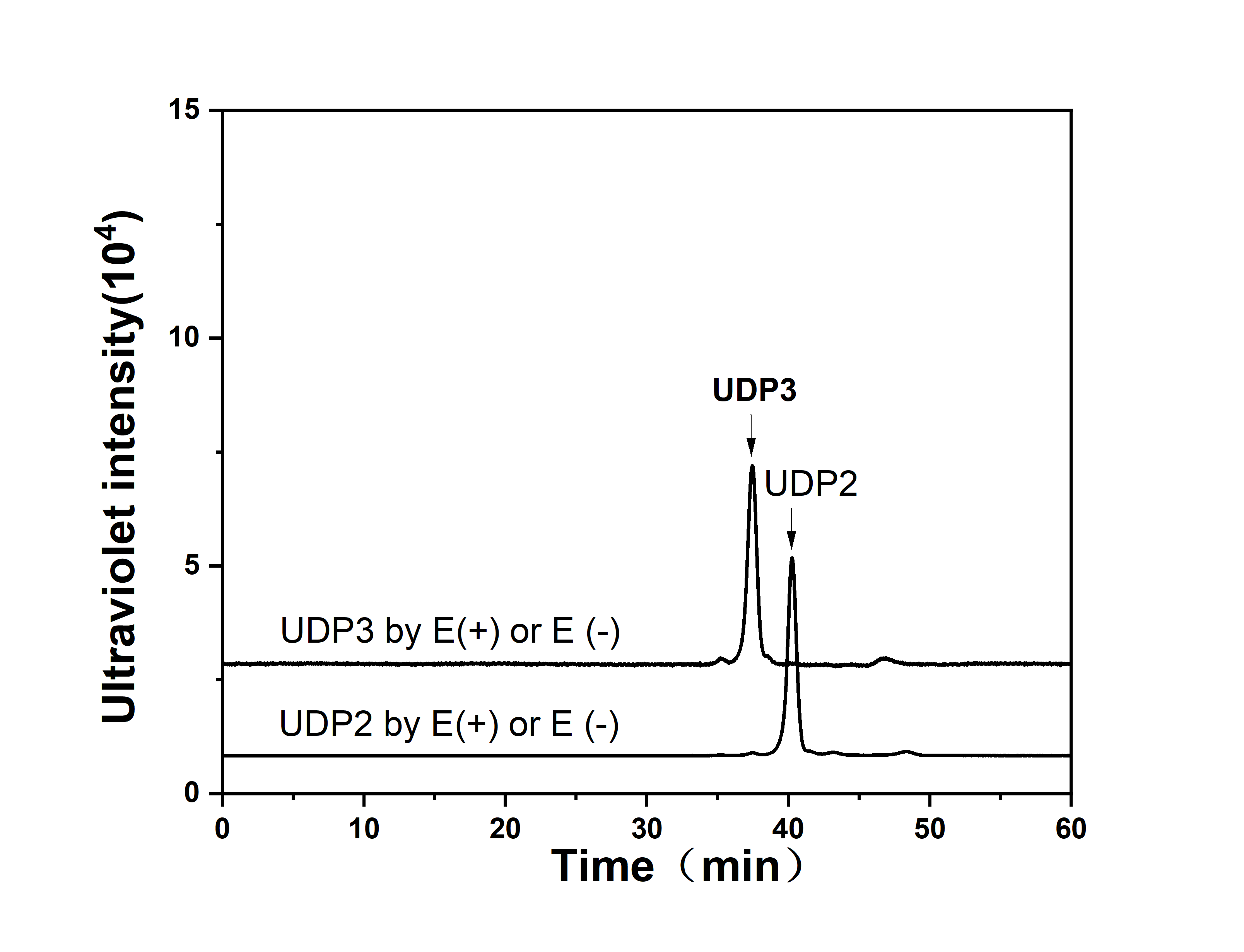

Supplement: SUPPLEMENTARY FIGURE S4 — Degradation patterns of Aly35 and its truncated proteins Aly35-CD1 and Aly35-CD2 toward unsaturated fractions of trisaccharide and disaccharide. Degradation of ~20 μg UDP3 fractions and degradation of ~20 μg UDP2 fractions for 12h by 1 μg three enzymes at 30 °C individually. E (−), without the enzyme. HPLC analyses were performed using a Superdex 30 Increase 10/300 GL column monitored at 232 nm. [file Image_4.tif]
