# Supplementary material for: A bifunctional endolytic alginate lyase with two different lyase catalytic domains from Vibrio sp. H204
Source: Front Microbiol. 2024 Dec 13;15:1509599. doi: 10.3389/fmicb.2024.1509599 (PMC11671496; doi:10.3389/fmicb.2024.1509599)
Supplement: SUPPLEMENTARY TABLE S1 — The protein protein sequences were used to construct phylogenetic tree. [file Table_1.docx]

| Number | Name | Protein sequnce |
| --- | --- | --- |
| 1 | WP_132936395.1 Aly35 | MFIKSTKLLLISFSGLGLIGCGGNTATTSQDDKPNIPHQEHVAPYSIAKFQNILRNSDLQVSDPDGKEGNKTSDVKNGAFSDYKSDHFYAEKDSNYLVFKMSNYKMRSEVRERENFNISEQGVSRTLYADVRLPEINLAMASSPANHDEVTFLQIHNKGTDTSGTGAIPHPLLRIVWEQERNSITGHYWAVVKNNAIDCSLPSSASDCYATSYDRYDLGKADLNAFTRFEVKIGENTLTIKVNDEQKVNVDVSYWQHLLSYFKAGVYNQFENGEAKVQFKQLGLTKTDHTDSIAWNIDDWKLTIPASKNDWYGFGGDSAAELEPERCNSSKDPLSNEESVYQREIDLSYFNVIDGSMHFRADMGYGTSTANSSYIRSELRELYISTNSPDCSTSDEETSW  YIEDSRTGATSHTLNATLRINEYPKIDGQLPKVVVGQIHGWKISQALVKLLWEGDNKPVRVILNDNYKLDNNKDCTDCNAFSVKLGTYAVNEDWQYTIRADKEGLFLATYDADGSNMVSHTLKWGEAYSDTANNKSYTLTERWASPDIAFYFKAGIYPQFKPDNAYRGEIFDVSFSALSTLHQ |
| 2 | HCZ9037888.1 polysaccharide lyase family 7 protein [Vibrio alginolyticus] | MFIKSTKLLLISFSGLGLIGCGGNTATTSQDDKPNIPHQEHVAPYSIAKFQNILRNSDLQVSDPDGKEGNKTSDVKNGAFSDYKSDHFYAEKDSNYLVFKMSNYKMRSEVRERENFNISEQGVSRTLYADVRLPEINLAMASSPANHDEVTFLQIHNKGTDTSGTGAIPHPLLRIVWEQERNSITGHYWAVVKNNAIDCSLPSSASDCYATSYDRYDLGKADLNAFTRFEVKIGENTLTIKVNDEQKVNVDVSYWQHLLSYFKAGVYNQFENGEAKVQFKQLGLTKTDHTDSIAWNIDDWKLTIPASKNDWYGFGGDSAAELEPERCNSSKDPLSNEESVYQREIDLSYFNVIDGSMHFRADMGYGTSTANSSYIRSELRELYISTNSPDCSTSDEETSW  YIEDSRTGATSHTLNATLRINEYPKIDGQLPKVVVGQIHGWKISQALVKLLWEGDNKPVRVILNDNYKLDNNKDCTDCNAFSVKLGTYAVNEDWQYTIRADKEGLFLATYDADGSNMVSHTLKWGEAYSDTANNKSYTLTERWASPDIAFYFKAGVYPQFKPDNAYRGEIFDVSFSALSTLHQ |
| 3 | WP_253657125.1 polysaccharide lyase family 7 protein [Vibrio sp. Y58_MX_L22] | MFIKSTKLLLISFSGLGLIGCGGNTATTSQDDKPNIPHQEHVAPYSIAKFQNILRNSDLQVSDPDGKEGNKTSDVKNGAFSDYKSDHFYAEKDSNYLVFKMSNYKMRSEVRERENFNISEQGVSRTLYADVRLPEINLAMASSPANHDEVTFLQIHNKGTDTSGTGAIPHPLLRIVWEQERNSITGHYWAVVKNNAIDCSLPSSASDCYATSYDRYDLGKADLNAFTRFEVKIGENTLTIKVNDEQKVNVDVSYWQHLLSYFKAGVYNQFENGEAKVQFKQLGLTKTDHTDSIAWNIDDWKLTIPASKNDWYGFGGDSAAELEPERCNSSKDPLSNEESVYQREIDLSYFNVIDGSMHFRADMGYGTSTANSSYIRSELRELYISTNSPDCSTSDEETSWYIEDSRTGATSHTLNATLRINEYPKIDGQLPKVVVGQIHGWKISQALVKLLWEGDNKPVRVILNDNYKLDNNKDCTDCNAFSVKLGTYAVNEDWQYTIRADKEGLFLATYDADGSNMISHTLKWGEAYSDTANNKSYTLTERWASPDIAFYFKAGIYPQFKPDNAYRGEIFDVSFSALSTLHQ |
| 4 | ENP8354289.1 polysaccharide lyase family 7 protein [Vibrio alginolyticus] | MFIKSTKLLLISFSGLGLIGCGGNTATTSQDDKPNIPHQEHVAPYSIAKFQNILRNSDLQVSDPDGKEGNKTSDVKNGAFSDYKSDHFYAEKDSNYLVFKMSNYKMRSEVRERENFNISEQGVSRTLYADVRLPEINLAMASSPANHDEVTFLQIHNKATDTSGTGAIPHPLLRIVWEQERNSITGHYWAVVKNNAIDCSLPSSASDCYATSYDRYDLGKADLNAFTRFEVKIGENTLTIKVNDEQKVNVDVSYWQHLLSYFKAGVYNQFENGEAKVQFKQLGLTKTDHTDSIAWNIDDWKLTIPASKNDWYGFGGDSAAELEPERCNSSKDPLSNEESVYQREIDLSYFNVIDGSMHFRADMGYGTSTANSSYIRSELRELYISTNSPDCSTSDEETSWYIEDSRTGATSHTLNATLRINEYPKIDGQLPKVVVGQIHGWKISQALVKLLWEGDNKPVRVILNDNYKLDNNKDCTDCNAFSVKLGTYAVNEDWQYTIRADKEGLFLATYDADGSNMVSHTLKWGEAYSDTANNKSYTLTERWASPDIAFYFKAGIYPQFKPDNAYRGEIFDVSFSALSTLHQ |
| 5 | WP_225466297.1 polysaccharide lyase family 7 protein [Vibrio] | MFIKSTKLLLISFSGLGLIGCGGNTATTSQDDKPNIPHQEHVAPYSIAKFQNILRNSDLQVSDPDGKEGNKTSDVKNGAFSDYKSDHFYAEKDSNYLVFKMSNYKMRSEVRERENFNISEQGVSRTLYADVRLPEINLAMASSPANHDEVTFLQIHNKGTDTSGTGAIPHPLLRIVWEQERNSITGHYWAVVKNNAIDCSLPSSASDCYATSYDRYDLGKADLNAFTRFEVKIGENTLTIKVNDEQKVNVDVSYWQHLLSYFKAGVYNQFENGEAKVQFKQLGLTKTDHTDSIAWNIDDWKLTIPASKNDWYGFGGDSAAELEPERCNSSKDPLSNEESVYQREIDLSYFNVIDGSMHFRADMGYGTSTANSSYIRSELRELYISTNSPDCSTSDEETSWYIEDSRTGATSHTLNATLRINEYPKIDGQLPKVVVGQIHGWKISQALVKLLWEGDNKPVRVILNDNYKLDNNKDCTDCNAFSVKLGTYAVNEDWQYTIRADKEGLFLATYDADGSNMVSHTLKWGEAYSDTANNKSYTLTERWASPDIAFYFKAGIYPQFKPDNAYRGEIFDVSFSALSTLHR |
| 6 | EKZ8662061.1 polysaccharide lyase family 7 protein [Vibrio alginolyticus] | MFIKSTKLLLISFSGLGLIGCGGNTATTSQDDKPNIPHQEHVAPYSIAKFQNILRNSDLQVSDPDGKEGDKTSDVKNGAFSDYKSDHFYAEKDSNYLVFKMSNYKMRSEVRERENFNISEQGVSRTLYADVRLPEINLAMASSPANHDEVTFLQIHNKGTDTSGTGAIPHPLLRIVWEQERNSITGHYWAVVKNNAIDCSLPSSASDCYATSYDRYDLGKADLNAFTRFEVKIGENTLTIKVNDEQKVNVDVSYWQHLLSYFKAGVYNQFENGEAKVQFKQLGLTKTDHTDSIAWNIDDWKLTIPASKNDWYGFGGDSAAELEPERCNSSKDPLSNEESVYQREIDLSYFNVIDGSMHFRADMGYGTSTANSSYIRSELRELYISTNSPDCSTSDEETSWYIEDSRTGATSHTLNATLRINEYPKIDGQLPKVVVGQIHGWKISQALVKLLWEGDNKPVRVILNDNYKLDNNKDCTDCNAFSVKLGTYAVNEDWQYTIRADKEGLFLATYDADGSNMVSHTLKWGEAYSDTANNKSYTLTERWASPDIAFYFKAGIYPQFKPDNAYRGEIFDVSFSALSTLHQ |
| 7 | WP_054578989.1 polysaccharide lyase family 7 protein [Vibrio alginolyticus] | MFIKSTKLLLISFSGLGLIGCGGNTATTSQDDKPNIPHQEHVAPYSIAKFQNILRNSDLQVSDPDGKEGNKTSDVKNGAFSDYKSDHFYAEKDSNYLVFKMSNYKMRSEVRERENFNISEQGVSRTLYADVRLPEINLAMASSPANHDEVTFLQIHNKGTDTSGTGAIPHPLLRIVWEQERNSITGHYWAVVKNNAIDCSLPSSASDCYATSYDRYDLGKADLNAFTRFEVKIGENTLTIKVNDEQKVNVDVSYWQHLLSYFKAGVYNQFENGEAKVQFKQLGLTKTDHTDSIAWNIDDWKLTIPASKNDWYGFGGDSAAELEPERCNSSKDPLSNEESVYQREIDLSYFNVIDGSMHFRADMGYGTSTANSSYIRSELRELYISTNSPDCSTSDEETSWYIEDSRTGATSHTLNATLRINEYPKIDGQFPKVVVGQIHGWKISQALVKLLWEGDNKPVRVILNDNYKLDNNKDCTDCNAFSVKLGTYAVNEDWQYTIRADKEGLFLATYDADGSNMVSHTLKWGEAYSDTANNKSYTLTERWASPDIAFYFKAGIYPQFKPDNAYRGEIFDVSFSALSTLHQ |
| 8 | WP_054574335.1 polysaccharide lyase family 7 protein [Vibrio alginolyticus] | MFIKSTKLLLISFSGLGLIGCGGNTATTSQDDKPNIPHQEHVAPYSIAKFQNILRNSDLQVSDPDGKEGNKTSDVKNGAFSDYKSDHFYAEKDSNYLVFKMSNYKMRSEVRERENFNISEQGVSRTLYADVRLPEINLAMTSSPANHDEVTFLQIHNKGTDTSGTGAIPHPLLRIVWEQERNSITGHYWAVVKNNAIDCSLPSSASDCYATSYDRYDLGKADLNAFTRFEVKIGENTLTIKVNDEQKVNVDVSYWQHLLSYFKAGVYNQFENGEAKVQFKQLGLTKTDHTDSIAWNIDDWKLTIPASKNDWYGFGGDSAAELEPERCNSSKDPLSNEESVYQREIDLSYFNVIDGSMHFRADMGYGTSTANSSYIRSELRELYISTNSPDCSTSDEETSWYIEDSRTGATSHTLNATLRINEYPKIDGQLPKVVVGQIHGWKISQALVKLLWEGDNKPVRVILNDNYKLDNNKDCTDCNAFSVKLGTYAVNEDWQYTIRADKEGLFLATYDADGSNMVSHTLKWGEAYSDTANNKSYTLTERWASPDIAFYFKAGIYPQFKPDNAYRGEIFDVSFSALSTLHQ |
| 9 | WP_213893367.1 polysaccharide lyase family 7 protein [Vibrio alginolyticus] | MFIKSTKLLLISFSGLGLIGCGGNTATTSQDDKPNIPHQEHVAPYSIAKFQNILRNSDLQVSDPDGKEGNKTSDVKNGAFSDYKSDHFYAEKDSNYLVFKMSNYKMRSEVRERENFNISEQGVSRTLYADVRLPEINLAMASSPANHDEVTFLQIHNKGTDTSGTGAIPHPLLRIVWEQERNSITGHYWAVVKNNAIDCSLPSSASDCYATSYDRYDLGKADLNAFTRFEVKIGENTLTIKVNDEQKVNVDVSYWQHLLSYFKAGVYNQFENGEAKVQFKQLGLTKTDHTDSIAWNIDDWKLTIPASKNDWYGFGGDSAAELEPERCNSSKDPLSNEESVYQREIDLSYFNVIDGSMHFRADMGYGTSTANSSYIRSELRELYISTNSPDCSTSDEETSWYIEDSRTGATSHTLNATLRINEYPKIDGQLPKVVVGQIHGWKISQALVKLLWEGDNKPVRVILNDNYKLDNNKDCTDCNAFSVKLGTYAVNEDWQYTIRADKEGLFLATYDADGSNMVSHTLKWGEAYSDTANNKSYTLTERWASPDIAFYFKAGIYPQFKPDNAYRGEIFDVSFSALSILHQ |
| 10 | EGQ9097667.1 polysaccharide lyase family 7 protein [Vibrio alginolyticus] | MFIKSTKLLLISFSGLRLIGCGGNTATTSQDDKPNIPHQEHVAPYSIAKFQNILRNSDLQVSDPDGKEGNKTSDVKNGAFSDYKSDHFYAEKDSNYLVFKMSNYKMRSEVRERENFNISEQGVSRTLYADVRLPEINLAMASSPANHDEVTFLQIHNKGTDTSGTGAIPHPLLRIVWEQERNSITGHYWAVVKNNAIDCSLPSSASDCYATSYDRYDLGKADLNAFTRFEVKIGENTLTIKVNDEQKVNVDVSYWQHLLSYFKAGVYNQFENGEAKVQFKQLGLTKTDHTDSIAWNIDDWKLTIPASKNDWYGFGGDSAAELEPERCNSSKDPLSNEESVYQREIDLSYFNVIDGSMHFRADMGYGTSTANSSYIRSELRELYISTNSPDCSTSDEETSWYIEDSRTGATSHTLNATLRINEYPKIDGQLPKVVVGQIHGWKISQALVKLLWEGDNKPVRVILNDNYKLDNNKDCTDCNAFSVKLGTYAVNEDWQYTIRADKEGLFLATYDADGSNMVSHTLKWGEAYSDTANNKSYTLTERWASPDIAFYFKAGIYPQFKPDNAYRGEIFDVSFSALSTLHQ |
| 11 | HCZ9051391.1 polysaccharide lyase family 7 protein [Vibrio alginolyticus] | MFIKSTKLLLISFSGLGLIGCGGNTATTSQDDKPNIPHQEHVAPYSIAKFQNILRNSDLQVSDPDGKEGNKTSDVKNGAFSDYKSDHFYAEKDSNYLVFKMSNYKMRSEVRERENFNISEQGVSRTLYADVRLPEINLAMASSPANHDEVTFLQIHNKGTDTSGTGAIPHPLLRIVWEQERNSITGHYWAVVKNNAIDCSLPSSASDCYATSYDRYDLGKADLNAFTRFEVKIGENTLTIKVNDEQKVNVDVSYWQHLLSYFKAGVYNQFENGEAKVQFKQLGLTKTDHTDSIAWNIDDWKLTIPASKNDWYGFGGDSAAELEPERCNSSKAPLSNEESVYQREIDLSYFNVIDGSMHFRADMGYGTSTANSSYIRSELRELYISTNSPDCSTSDEETSWYIEDSRTGATSHTLNATLRINEYPKIDGQLPKVVVGQIHGWKISQALVKLLWEGDNKPVRVILNDNYKLDNNKDCTDCNAFSVKLGTYAVNEDWQYTIRADKEGLFLATYDADGSNMVSHTLKWGEAYSDTANNKSYTLTERWASPDIAFYFKAGIYPQFKPDNAYRGEIFDVSFSALSTLHQ |
| 12 | ENP8391589.1 polysaccharide lyase family 7 protein [Vibrio alginolyticus] | MFIKSTKLLLISFSGLGLIGCGGNTATTSQDDKPNIPHQEHVAPYSIAKFQNILRNSDLQVSDPDGKEGNKTSDVKNGAFSDYKSDHFYAEKDSNYLVFKMSNYKMRSEVRERENFNISEQGVSRTLYADVRLPEINLAMASSPANHDEVTFLQIHNKGTDTSGTGAIPHPLLRIVWEQERNSITGHYWAVVKNNAIDCSLPSSASDCYATSYDRYDLGKADLNAFTRFEVKIGENTLTIKVNDEQKVNVDVSYWQHLLSYFKAGVYNQFENGEAKVQFKQLGLTKTDHTDSIAWNIDDWKLTIPASKNDWYGFGGDSAAELEPERCNSSKDPLSNEESVYQREIDLSYFNVIDGSMHFRADMGYGTSTANSSYIRSELRELYISTNSPDCSTSDEETSWYIEDSRTGATSHTLNATLRINEYPKINGQLPKVVVGQIHGWKISQALVKLLWEGDNKPVRVILNDNYKLDNNKDCTDCNAFSVKLGTYAVNEDWQYTIRADKEGLFLATYDADGSNMVSHTLKWGEAYSDTANNKSYTLTERWASPDIAFYFKAGIYPQFKPDNAYRGEIFDVSFSALSTLHR |
| 13 | WP_205391143.1 MULTISPECIES: polysaccharide lyase family 7 protein [Vibrio] | MFIKSTKLLLISFSGLGLIGCGGNTATTSQDDKPNIPHQEHVAPYSIAKFQNILRNSDLQVSDPDGKEGNKTSDVKNGAFSDYKSDHFYAEKDSNYLVFKMSNYKMRSEVRERENFNISEQGVSRTLYADVRLPEINLAMASSPANHDEVTFLQIHNKGTDTSGTGAIPHPLLRIVWEQERNSITGHYWAVVKNNAIDCSLPSSASDCYATSYDRYDLGKADLNAFTRFEVKIGENTLTIKVNDEQKVNVDVSYWQHLLSYFKAGVYNQFENGEAKVQFKQLGLTKTDHTDSIAWNIDDWKLTIPASKNDWYGFGGDSAAELEPERCNSSKDLLSNEESVYQREIDLSYFNVIDGSMHFRADMGYGTSTANSSYIRSELRELYISTNSPDCSTSDEETSWYIEDSRTGATSHTLNATLRINEYPKIDGQLPKVVVGQIHGWKISQALVKLLWEGDNKPVRVILNDNYKLDNNKDCTDCNAFSVKLGTYAVNEDWQYTIRADKEGLFLATYDADGSNMVSHTLKWGEAYSDTANNKSYTLTERWASPDIAFYFKAGIYPQFKPDNAYRGEIFDVSFSALSTLHQ |
| 14 | WP_005388516.1 polysaccharide lyase family 7 protein [Vibrio alginolyticus] | MFIKSTKLLLISFSGLGLIGCGGNTATTSQDDKPNIPHQEHVAPYSIAKFQNILRNSDLQVSDPDGKEGNKTSDVKNGAFSDYKSDHFYAEKDSNYLVFKMSNYKMRSEVRERENFNISEQGVSRTLYADVRLPEINLAMASSPANHDEVTFLQIHNKGTDTSGTGAIPHPLLRIVWEQERNSITGHYWAVVKNNAIDCSLPSSASDCYATSYDRYDLGKADLNAFTRFEVKIGENTLTIKVNDKQKVNVDVSYWQHLLSYFKAGVYNQFENGEAKVQFKQLGLTKTDHTDSIAWNIDDWKLTIPASKNDWYGFGGDSAAELEPERCNSSKDPLSNEESVYQREIDLSYFNVIDGSMHFRADMGYGTSTANSSYIRSELRELYISTNSPDCSTSDEETSWYIEDSRTGATSHTLNATLRINEYPKIDGQLPKVVVGQIHGWKISQALVKLLWEGDNKPVRVILNDNYKLDNNKDCTDCNAFSVKLGTYAVNEDWQYTIRADKEGLFLATYDADGSNMISHTLKWGEAYSDTANNKSYTLTERWASPDIAFYFKAGIYPQFKPDNAYRGEIFDVSFSALSTLHQ |
| 15 | WP_285335203.1 polysaccharide lyase family 7 protein [Vibrio sp. B513a] | MFIKSTKLLLISFSGLGLIGCGGNTATTSQDDKPNIPHQEHVAPYSIAKFQNILRNSDLQVSDPDGKEENKTSDVKNGAFSDYKSDHFYAEKDSNYLVFKMSNYKMRSEVRERENFNISEQGVSRTLYADVRLPEINLAMASSPANHDEVTFLQIHNKATDTSGTGAIPHPLLRIVWEQERNSITGHYWAVVKNNAIDCSLPSSASDCYATSYDRYDLGKADLNAFTRFEVKIGENTLTIKVNDEQKVNVDVSYWQHLLSYFKAGVYNQFENGEAKVQFKQLGLTKTDHTDSIAWNIDDWKLTIPASKNDWYGFGGDSAAELEPERCNSSKDPLSNEESVYQREIDLSYFNVIDGSMHFRADMGYGTSTANSSYIRSELRELYISTNSPDCSTSDEETSWYIEDSRTGATSHTLNATLRINEYPKIDGQLPKVVVGQIHGWKISQALVKLLWEGDNKPVRVILNDNYKLDNNKDCTDCNAFSVKLGTYAVNEDWQYTIRADKEGLFLATYDADGSNMVSHTLKWGEAYSDTANNKSYTLTERWASPDIAFYFKAGIYPQFKPDNAYRGEIFDVSFSALSTLHQ |
| 16 | WP_182016129.1 polysaccharide lyase family 7 protein [Vibrio alginolyticus] | MFIKSTKLLLISFSGLGLIGCGGNTATTSQDDKPNIPHQEHVAPYSIAKFQNILRNSDLQVSDPDGKEGNKTSDVKNGAFSDYKSDHFYAEKDSNYLVFKMSNYKMRSEVRERENFNISEQCVSRTLYADVRLPEINLAMASSPANHDEVTFLQIHNKGTDTSGTGAIPHPLLRIVWEQERNSITGHYWAVVKNNAIDCSLPSSASDCYATSYDRYDLGKADLNAFTRFEVKIGENTLTIKVNDEQKVNVDVSYWQHLLSYFKAGVYNQFENGEAKVQFKQLGLTKTDHTDSIAWNIDDWKLTIPASKNDWYGFGGDSAAELEPERCNSSKDPLSNEESVYQREIDLSYFNVIDGSMHFRADMGYGTSTANSSYIRSELRELYISTNSPDCSTSDEETSWYIEDSRTGATSHTLNATLRINEYPKIDGQLPKVVVGQIHGWKISQALVKLLWEGDNKPVRVILNDNYKLDNNKDCTDCNAFSVKLGTYAVNEDWQYTIRADKEGLFLATYDADGSNMVSHTLKWGEAYSDTANNKSYTLTERWASPDIAFYFKAGIYPQFKPDNAYRGEIFDVSFSALSTLHR |
| 17 | WP_033906978.1 polysaccharide lyase family 7 protein [Vibrio sp. OY15] | MFIKSTKLLLISFSGLGLIGCGGNTATTSQDDKPNIPHQAHVAPYSIAKFQNILRNSDLQVSDPDGKEGNKTSDVKNGAFSDYKSDHFYAEKDSNYLVFKMSNYKMRSEVRERENFNISEQGVSRTLYADVRLPEINLAMASSPANHDEVTFLQIHNKGTDTSGTGAIPHPLLRIVWEQERNSITGHYWAVVKNNAIDCSLPSSASDCYATSYDRYDLGKADLNAFTRFEVKIGENTLTIKVNDEQKVNVDVSYWQHLLSYFKAGVYNQFENGEAKVQFKQLGLTKTDHTDSIAWNIDDWKLTIPASKNDWYGFGGDSAAELEPERCNSSKDLLSNEESVYQREIDLSYFNVIDGSMHFRADMGYGTSTANSSYIRSELRELYISTNSPDCSTSDEETSWYIEDSRTGATSHTLNATLRINEYPKIDGQLPKVVVGQIHGWKISQALVKLLWEGDNKPVRVILNDNYKLDNNKDCTDCNAFSVKLGTYAVNEDWQYTIRADKEGLFLATYDADGSNMVSHTLKWGEAYSDTANNKSYTLTERWASPDIAFYFKAGIYPQFKPDNAYRGEIFDVSFSALSTLHQ |
| 18 | WP_258482134.1 polysaccharide lyase family 7 protein [Vibrio alginolyticus] | MFIKSTKLLLISFSGLGLIGCGGNTATTSQDDKPNIPHQEHVAPYSIAKFQNILRNSDLQVSDPDGKEGNKTSDVKNGAFSDYKSDHFYAEKDSNYLVFKMSNYKMRSEVRERENFNISEQGVSRTLYADVRLPEINLAMASSPANHDEVTFLQIHNKGTDTSGTGAIPHPLLRIVWEQERNSITGHYWAVVKNNAIDCSLPSSASDCYATSYDRYDLGKADLNAFTRFEVKIGENTLTIKVNDEQKVNVDVSYWQHLLSYFKAGVYNQFENGEAKVQFKQLGLTKTDHTDSIAWNIDDWKLTIPASKNDWYGFGGDSAAELEPERCNSSKDLLSNEESVYQREIDLSYFNVIDGSMHFRADMGYGTSTANSSYIRSELRELYISTNSPDCSTSDEETSWYIEDSRTGATSHTLNATLRINEYPKIDGQLPKVVVGQIHGWKISQALVKLLWEGDNKPVRVILNDNYKLDNNKDCTDCNAFSVKLGTYAVNEDWQYTIRADKEGLFLATYDADGSNMVSHTLKWGEAYSDTASNKSYTLTERWASPDIAFYFKAGIYPQFKPDNAYRGEIFDVSFSALSTLHQ |
| 19 | WP_158155248.1 polysaccharide lyase family 7 protein [Vibrio alginolyticus] | MFIKSTKLLLISFSGLGLIGCGGNTATTSQDDKPNIPHQEHVAPYSIAKFQNILRNSDLQVSDPDGKEGNKTSDVKNGAFSDYKSDHFYAEKDSNYLVFKMSNYKMRSEVRERENFNISEQGVFRTLYADVRLPEINLAMASSPANHDEVTFLQIHNKGTDTSGTGAIPHPLLRIVWEQERNSITGHYWAVVKNNAIDCSLPSSASDCYATSYVRYDLGKADLNAFTRFEVKIGENTLTIKVNDEQKVNVDVSYWQHLLSYFKAGVYNQFENGEAKVQFKQLGLTKTDHTDSIAWNIDDWKLTIPASKNDWYGFGGDSAAELEPERCNSSKDPLSNEESVYQREIDLSYFNVIDGSMHFRADMGYGTSTANSSYIRSELRELYISTNSPDCSTSDEETSWYIEDSRTGATSHTLNATLRINEYPKIDGQLPKVVVGQIHGWKISQALVKLLWEGDNKPVRVILNDNYKLDNNKDCTDCNAFSVKLGTYAVNEDWQYTIRADKEGLFLATYDADGSNMVSHTLKWGEAYSDTANNKSYTLTERWASPDIAFYFKAGIYPQFKPDNAYRGEIFDVSFSALSTLHQ |
| 20 | WP_318074641.1 polysaccharide lyase family 7 protein [unclassified Vibrio] | MFIKSTKLLLISFSGLGLIGCGGNTATTSQDDKPNIPHQEHVAPYSIAKFQNILRNSDLQVSDPDGKEGNKTSDVKNGAFSDYKSDHFYAEKDSNYLVFKMSNYKMRSEVRERENFNTSEQGVSRTLYADVRLPEINLAMASSPANHDEVTFLQIHNKGTDTSGTGAIPHPLLRIVWEQERNSITGHYWAVVKNNAIDCSLPSSASDCYATSYDRYDLGKADLNAFTRFEVKIGENTLTIKVNDEQKVNVDVSYWQHLLSYFKAGVYNQFENGEAKVQFKQLGLTKTDHTDSIAWNIDDWKLTIPASKNDWYGFGGDSAAELEPERCNSSKDLLSNEESVYQREIDLSYFNVIDGSMHFRADMGYGTSTANSSYIRSELRELYISTNSPDCSTSDEETSWYIEDSRTGATSHTLNATLRINEYPKIDGQLPKVVVGQIHGWKISQALVKLLWEGDNKPVRVILNDNYKLDNNKDCTDCNAFSVKLGTYAVNEDWQYTIRADKEGLFLATYDADGSNMVSHTLKWGEAYSDTANNKSYTLTERWASPDIAFYFKAGIYPQFKPDNAYRGEIFDVSFSALSTLHQ |
| 21 | WP_213901659.1 polysaccharide lyase family 7 protein [Vibrio alginolyticus] | MFIKSTKLLLISFSGLGLIGCGGNTSPTSQDDKPNIPHQEHVAPYSIAKFQNILRNSDLQVSDPDGKEGNKTSDVKNGAFSDYKSDHFYAEKDSNYLVFKMSNYKMRSEVRERENFNISEQGVSRTLYADVRLPEINLAMASSPANHDEVTFLQIHNKGTDTSGTGAIPHPLLRIVWEQERNSITGHYWAVVKNNAIDCSLPSSASDCYATSYDRYDLGKADLNAFTRFEVKIGENTLTIKVNDEQKVNVDVSYWQHLLSYFKAGVYNQFENGEAKVQFKQLGLTKTDHTDSIAWNIDDWKLTIPASKNDWYGFGGDSAAELEPERCNSSKDPLSNEESVYQREIDLSYFNVIDGSMHFRAGMGYGTSTANSSYIRSELRELYISTNSPDCSTSDEETSWYIEDSRTGATSHTLNATLRINEYPKIDGQLPKVVVGQIHGWKISQALVKLLWEGDNKPVRVILNDNYKLDNNKDCTDCNAFSVKLGTYAVNEDWQYTIRADKEGLFLATYDADGSNMVSHTLKWGEAYSDTANNKSYTLTERWASPDIAFYFKAGIYPQFKPDNAYRGEIFDVSFSALSTLHQ |
| 22 | WP_337222467.1 polysaccharide lyase family 7 protein [Vibrio alginolyticus] | MFIKSTKLLLISFSGLGLIGCGGNTATTSQDDKPNIPHQEHVAPYSIAKFQNILRNSDLQVSDPDGKEGNKTSDVKNGAFSDYKSDHFYAEKDSNYLVFKMSNYKMRSEVRERENFNISEQGVSRTLYADVRLPEINLAMASSPANHDEVTFLQIHNKGTDTSGTGAIPHPLLRIVWEQERNSITGHYWAVVKNNAIDCSLPSSASDCYATSYDRYDLGKADLNAFTRFEVKIGENTLTIKVNDEQKVNVDVSYWQHLLSYFKAGVYNQFENGEAKVQFKQLGLTKTDHTDSIAWNIDDWKLTIPTSKNDWYGFGGDSAAELEPERCNSSKDLLSNEESVYQREIDLSYFNVIDGSMHFRADMGYGTSTANSSYIRSELRELYISTNSPDCSTSDEETSWYIEDSRTGATSHTLNATLRINEYPKIDGQLPKVVVGQIHGWKISQALVKLLWEGDNKPVRVILNDNYKLDNNKDCTDCNAFSVKLGTYAVNEDWQYTIRADKEGLFLASYDADGSNMVSHTLKWGEAYSDTANNKSYTLTERWASPDIAFYFKAGIYPQFKPDNAYRGEIFDVSFSALSTLHQ |
| 23 | WP_191115832.1 polysaccharide lyase family 7 protein [Vibrio alginolyticus] | MFIKSTKLLLISFSGLGLIGCGGNTSPTSQDDKPNIPHQEHVAPYSIAKFQNILRNSDLQVSDPDGKEGNKTSDVKNGAFSDYKSDHFYAEKDSNYLVFKMSNYKMRSEVRERENFNISEQGVSRTLYADVRLPEINLAMASSPANHDEVTFLQIHNKGTDTSGTGAIPHPLLRIVWEQERNSITDHYWAVVKNNAIDCSLPSSASDCYATSYDRYDLGKADLNAFTRFEVKIGENTLTIKVNDEQKVNVDVSYWQHLLSYFKAGVYNQFENGEAKVQFKQLGLTKTDHTDSIAWNIDDWKLTIPASKNDWYGFGGDSAAELEPERCNSSKDPLSNEESVYQREIDLSYFNVIDGSMHFRADMGYGTSTANSSYIRSELRELYISTNSPDCSTSDEETSWYIEDSRTGATSHTLNATLRINEYPKIDGQLPKVVVGQIHGWKISQALVKLLWEGDNKPVRVILNDNYKLDNNKDCTDCNAFSVKLGTYAVNEDWQYTIRADKEGLFLATYDADGSNMVSHTLKWGEAYSDTANNKSYTLTERWASPDIAFYFKAGIYPQFKPDNAYRGEIFDVSFSALSTLHQ |
| 24 | WP_258454211.1 polysaccharide lyase family 7 protein [Vibrio alginolyticus] | MFIKSTKLLLISFSGLGLIGCGGNTATTSQDDKPNIPHQEHVAPYSIAKFQNILRNSDLQVSDPDGKEGNKTSDVKNGAFSDYKSDHFYAEKDSNYLVFKMSNYKMRSEVRERENFNISEQGVFRTLYADVRLPEINLAMASSPANHDEVTFLQIHNKGTDTSGTGAIPHPLLRIVWEQERNSITGHYWAVVKNNAIDCSLPSSASDCYATSYNRYDLGKADLNAFTRFEVKIGENTLTIKVNDEQKVNVDVSYWQHLLSYFKAGVYNQFENGEAKVQFKQLGLTKTDHTDSIAWNIDDWKLTIPASKNDWYGFGGDSAAELEPERCNSSKDLLSNEESVYQREIDLSYFNVIDGSMHFRADMGYGTSTANSSYIRSELRELYISTNSPDCSTSDEETSWYIEDSRTGATSHTLNATLRINEYPKIDGQLPKVVVGQIHGWKISQALVKLLWEGDNKPVRVILNDNYKLDNNKDCTDCNAFSVKLGTYAVNEDWQYTIRADKEGLFLATYDADGSNMVSHTLKWGEAYSDTANNKSYTLTERWASPDIAFYFKAGIYPQFKPDNAYRGEIFDVSFSALSTLHQ |
| 25 | WP_213866284.1 polysaccharide lyase family 7 protein [Vibrio] | MFIKSTKLLLISFSGLGLIGCGGNTSPTSQDDKPNIPHQEHVAPYSIAKFQNILSNSDLQVSDPNGKEGNKTSDVKNGAFSDYKSDHFYAEKDSNYLVFKMSNYKMRSEVRERENFNISEQGVSRTLYADVRLPEINLAMASSPANHDEVTFLQIHNKGTDTSGTGAIPHPLLRIVWEQERNSITGHYWAVVKNNAIDCSLPSSASDCYATSYDRYDLGKADLNAFTRFEVKIGENTLTIKVNDEQKVNVDVSYWQHLLSYFKAGVYNQFENGEAKVQFKQLGLTKTDHTDSIAWNIDDWKLTIPASKNDWYGFGGDSAAELEPERCNSSKDPLSNEESVYQREIDLSYFNVIDGSMHFRADMGYGTSTANSSYIRSELRELYISTNSPDCSTSDEETSWYIEDSRTGATSHTLNATLRINEYPKIDGQLPKVVVGQIHGWKISQALVKLLWEGDNKPVRVILNDNYKLDNNKDCTDCNAFSVKLGTYAVNEDWQYTIRADKEGLFLATYDADGSNMVSHTLKWGEAYSDTANNKSYTLTERWASPDIAFYFKAGIYPQFKPDNAYRGEIFDVSFSALSTLHQ |
| 26 | WP_258485477.1 polysaccharide lyase family 7 protein [Vibrio alginolyticus] | MFIKSTKLLLISFSGLGLIGCGGNTSPTSQDDKPNIPHQEHVAPYSIAKFQNILSNSDLQVSDPNGKEGNKTSDVKNGAFSDYKSDHFYAEKDSNYLVFKMSNYKMRSEVRERENFNISEQGVSRTLYADVRLPEINLAMASSPANHDEVTFLQIHNKGTDTSGTGAIPHPLLRIVWEQERNSITGHYWAVVKNNAIDCSLPSSASDCYATSYDRYDLGKADLNAFTRFEVKIGENTLTIKVNDEQKVNVDVSYWQHLLSYFKAGVYNQFENGEAKVQFKQLGLTKTDHTDSIAWNIDDWKLTIPTSKNDWYGFGGDSAAELEPERCNSSKDPLSNEESVYQREIDLSYFNVIDGSMHFRADMGYGTSTANSSYIRSELRELYISTNSPDCSTSDEETSWYIEDSRTGATSHTLNATLRINEYPKIDGQLPKVVVGQIHGWKISQALVKLLWEGDNKPVRVILNDNYKLDNNKDCTDCNAFSVKLGTYAVNEDWQYTIRADKEGLFLATYDADGSNMVSHTLKWGEAYSDTANNKSYTLTERWASPDIAFYFKAGIYPQFKPDNAYRGEIFDVSFSALSTLHQ |
| 27 | WP_395240493.1 polysaccharide lyase family 7 protein [Vibrio alginolyticus] | MFIKSTKLLLISFSGLGLIGCGGNTSPTSQDDKPNIPHQEHVAPYSIAKFQNILSNSDLQVSDPNGKEGNKTSDVKNGAFSDYKSDHFYAEKDSNYLVFKMSNYKMRSEVRERENFNISEQGVSRTLYADVRLPEINLAMASSPANHDEVTFLQIHNKGTDTSGTGAIPHPLLRIVWEQERNSITGHYWAVVKNNAIDCSLPSSASDCYATSYDRYDLGKADLNAFTRFEVKIGENTLTIKVNDEQKVNVDVSYWQHLLSYFKAGVYNQFENGEAKVQFKQLGLTKTDHTDSIAWNIDDWKLTIPASKNDWYGFGGDSAAELEPERCNSSKDPLSNEESVYQREIDLSYFNVIDGSMHFRADMGYGTSTANSSYIRSELRELYISTNSPDCSTSDEETSWYIEDSRTGATSHTLNATLRINEYPKIDGQLPKVVVGQIHGWKISQALVKLLWEGDNKPVRVILNDNYKLDNNKDCTDCNAFSVKLGTYAVNEDWQYTIRADKEGLFLATYDADGCNMVSHTLKWGEAYSDTANNKSYTLTERWASPDIAFYFKAGIYPQFKPDNAYRGEIFDVSFSALSTLHQ |
| 28 | EKL9828462.1 polysaccharide lyase family 7 protein [Vibrio alginolyticus] | MFIKSTKLLLISFSGLGLIGCGGNTATTSQDDKPNIPHQEHVAPYSIAKFQNILSNSDLQVSDPNGEEGNKNSDVKNGAFSDYKSDHFYAENDSNYLVFKMSNYKMRSEVRERENFNISEQGVSRTLYADVRLPEINLAMASSPANHDEVTFLQIHNKGTDTSGTGAIPHPLLRIVWEQERNSITGHYWAVVKNNAIDCSLPSSASDCYATSYDRYDLGKADLNAFTRFEVKIGENTLTIKVNDEQKVNVDVSYWQHLLSYFKAGVYNQFENGEAKVQFKQLGLTKTDHTDSIAWNIDDWKLTIPASKNDWYGFGGDSAAELEPERCNSSKDPLSNEESVYQREIDLSYFNVIDGSMHFRADMGYGTSTANSSYIRSELRELYISTNSPDCSTSDEETSWYIEDSRTGATSHTLNATLRINEYPKIDGQLPKVVVGQIHGWKISQALVKLLWEGDNKPVRVILNDNYKLDNNKDCTDCNAFSVKLGTYAVNEDWQYTIRADKEGLFLATYDADGSNMVSHTLKWGEAYSDTANNKSYTLTERWASPDIAFYFKAGIYPQFKPDNAYRGEIFDVSFSALSTLHQ |
| 29 | WP_395237455.1 polysaccharide lyase family 7 protein [Vibrio alginolyticus] | MFIKSTKLLLISFSGLGLIGCGGNTATTSQDDKPNIPHQEHVAPYSIAKFQNILRNSDLQVSDPDGKEGNKTSDVKNGAFSDYKSDHFYAEKDSNYLVFKMSNYKMRSEVRERENFNISEQGVSRTLYADVRLPEINLAMASSPANHDEVTFLQIHNKGTDTSGTGAIPHPLLRIVWEQERNSITGHYWAVVKNNAIDCSLPSSASDCYATSYDRYDLGKADLNAFTRFEVKIGENTLTIKVNDEQKVNVDVSYWQHLLSYFKAGVYNQFENGEAKVQFNQLGLTKTDHTDSIAWNIDDWKLTIPASKNDWYGFGGDSAAELEPERCNSSKDLLSNEESVYQREIDLSSFNVIDGSMHFRADMGYGTSTANSSYIRSELRELYISTNSPDCSTSDEETSWYIEDSRTGATSHTLNATLRINEYPKIDGQLPKVVVGQIHGWKISQALVKLLWEGDNKPVRVILNDNYKLDNNKDCTDCNAFSVKLGTYAVNEDWQYTIRADKEGLFLASYDADGSNMVSHTLKWGEAYSDTANNKSYTLTERWASPDIAFYFKAGIYPQFKPDNAYRGEIFDVSFSALSTLHQ |
| 30 | WP_258482490.1 polysaccharide lyase family 7 protein [Vibrio alginolyticus] | MKSTKLLLISFSGLGLIGCGGNTATTSQDDKPNIPHQEHVAPYSIAKFQNILRNSDLQVSDPDGKEGNKTSDVKNGAFSDYKSDHFYAEKDSNYLVFKMSNYKMRSEVRERENFNISEQCVSRTLYADVRLPEINLAMASSPANHDEVTFLQIHNKGTDTSGTGAIPHPLLRIVWEQERNSITGHYWAVVKNNAIDCSLPSSASDCYATSYDRYDLGKADLNAFTRFEVKIGENTLTIKVNDEQKVNVDVSYWQHLLSYFKAGVYNQFENGEAKVQFKQLGLTKTDHTDSIAWNIDDWKLTIPASKNDWYGFGGDSAAELEPERCNSSKDPLSNEESVYQREIDLSYFNVIDGSMHFRADMGYGTSTANSSYIRSELRELYISTNSPDCSTSDEETSWYIEDSRTGATSHTLNATLRINEYPKIDGQLPKVVVGQIHGWKISQALVKLLWEGDNKPVRVILNDNYKLDNNKDCTDCNAFSVKLGTYAVNEDWQYTIRADKEGLFLATYDADGSNMVSHTLKWGEAYSDTANNKSYTLTERWASPDIAFYFKAGIYPQFKPDNAYRGEIFDVSFSALSTLHR |
| 31 | WP_053307758.1 polysaccharide lyase family 7 protein [Vibrio alginolyticus] | MFIKSTKLLLISFSGLGLIGCGGNTSPTSQDDKPNIPHQEHVAPYSIAKFQNILSNSDLQVSDPNGKEGNKTSDVKNGAFSDYKSDHFYAEKDSNYLVFKMSNYKMRSEVRERENFNISEQGVSRTLYADVRLPEINLAMASSPANHDEVTFLQIHNKGTDTSGTGAIPHPLLRIVWEQERNSITGHYWAVVKNNAIDCSLPSSASDCYATSYDRYDLGKADLNAFTRFEVKIGENTLTIKVNDEQKVNVDVSYWQHLLSYFKAGVYNQFENGEAKVQFKQLGLTKTDHTDSIAWNIDDWKLTIPTSKNDWYGFGGDSAAELEPERCNSSKDPLSNEESVYQREIDLSYFNVIDGSMHFRADMGYGTSTANSSYIRSELRELYISTNSPDCSTSDEETSWYIEDSRTGATSHTLNATLRINEYPKIDGQLPKVVVGQIHGWKISQALVKLLWEGDNKPVRVILNDNYKLDNNKDCTDCNAFSVKLGTYAVNEDWQYTIRADKEGLFLATYDADGSNMVSHTLKWGEAYSDTANNKSYTLTERWASPDIAFYFKAGMYPQFKPDNAYRGEIFDVSFSALSTLHQ |
| 32 | EIL2907684.1 polysaccharide lyase family 7 protein [Vibrio alginolyticus] | MFIKSTKLLLISFSGLGLIGCGGNTSPTSQDDKPNIPHQEHVAPYSIAKFQNILSNSDLQVSDPNGKEGNKTSDVKNGAFSDYKSDHFYAEKDSNYLVFKMSNYKMRSEVRERENFNISEQGVSRTLYADVRLPEINLAMASSPANHDEVTFLQIHNKGTDTSGTGAIPHPLLRIVWEQERNSITGHYWAVVKNNAIDCSLPSSASDCYATSYDRYDLGKADLNAFTRFEVKIGENTLTIKVNDEQKVNVDVSYWQHLLSYFKAGVYNQFENGEAKVQFKQLGLAKTDHTDSIAWNIDDWKLTIPTSKNDWYGFGGDSAAELEPERCNSSKDPLSNEESVYQREIDLSYFNVIDGSMHFRADMGYGTSTANSSYIRSELRELYISTNSPDCSTSDEETSWYIEDSRTGATSHTLNATLRINEYPKIDGQLPKVVVGQIHGWKISQALVKLLWEGDNKPVRVILNDNYKLDNNKDCTDCNAFSVKLGTYAVNEDWQYTIRADKEGLFLATYDADGSNMVSHTLKWGEAYSDTANNKSYTLTERWASPDIAFYFKAGIYPQFKPDNAYRGEIFDVSFSALSTLHQ |
| 33 | WP_258501229.1 polysaccharide lyase family 7 protein [Vibrio alginolyticus] | MFIKSTKLLLISFSGLGLIGCGGNTSPTSQDDKPNIPHQEHVAPYSIAKFQNILSNSDLQVSDPNGKEGNKTSDVKNGAFSDYKSDHFYAEKDSNYLVFKMSNYKMRSEVRERENFNISEQGVSRTLYADVRLPEINLAMASSPANHDEVTFLQIHNKGTDTSGTGAIPHPLLRIVWEQERNSITDHYWAVVKNNAIDCSLPSSASDCYATSYDRYDLGKADLNAFTRFEVKIGENTLTIKVNDEQKVNVDVSYWQHLLSYFKAGVYNQFENGEAKVQFKQLGLTKTDHTDSIAWNIDDWKLTIPASKNDWYGFGGDSAAELEPERCNSSKDPLSNEESVYQREIDLSYFNVIDGSMHFRADMGYGTSTANSSYIRSELRELYISTNSPDCSTSDEETSWYIEDSRTGATSHTLNATLRINEYPKIDGQLPKVVVGQIHGWKISQALVKLLWEGDNKPVRVILNDNYKLDNNKDCTDCNAFSVKLGTYAVNEDWQYTIRADKEGLFLATYDADGSNMVSHTLKWGEAYSDTANNKSYTLTERWASPDIAFYFKAGIYPQFKPDNAYRGEIFDVSFSALSTLHQ |
| 34 | WP_394630842.1 polysaccharide lyase family 7 protein [Vibrio alginolyticus] | MFIKSTKLLLISFSGLGLIGCGGNTSPTSQDDKPNIPHQEHVAPYSIAKFQNILSNSDLQVSDPNGKEGNKTSDVKNGAFSDYKSDHFYAEKDSNYLVFKMSNYKMRSEVRERENFNISEQGVSRTLYADVLLPEINLAMASSPANHDEVTFLQIHNKGTDTSGTGAIPHPLLRIVWEQERNSITGHYWAVVKNNAIDCSLPSSASDCYATSYDRYDLGKADLNAFTRFEVKIGENTLTIKVNDEQKVNVDVSYWQHLLSYFKAGVYNQFENGEAKVQFKQLGLTKTDHTDSIAWNIDDWKLTIPASKNDWYGFGGDSAAELEPERCNSSKDPLSNEESVYQREIDLSYFNVIDGSMHFRADMGYGTSTANSSYIRSELRELYISTNSPDCSTSDEETSWYIEDSRTGATSHTLNATLRINEYPKIDGQLPKVVVGQIHGWKISQALVKLLWEGDNKPVRVILNDNYKLDNNKDCTDCNAFSVKLGTYAVNEDWQYTIRADKEGLFLATYDADGSNMVSHTLKWGEAYSDTANNKSYTLTERWASPDIAFYFKAGIYPQFKPDNAYRGEIFDVSFSALSTLHQ |
| 35 | EJL6733319.1 polysaccharide lyase family 7 protein [Vibrio alginolyticus] | MFIKSTKLLLISFSGLGLIGCGGNTSPTSQDDKPNIPHQEHVAPYSIAKFQNILSNSDLQVSDPNGKEGNKTSDVKNGAFSDYKSDHFYAEKDSNYLVFKMSNYKMRSEVRERENFNISEQGVSRTLYADVRLPEINLAMASSPANHDEVTFLQIHNKGTDTSGTGAIPHPLLRIVWEQERNSITGHYWAVVKNNAIDCSLPSSASDCYATSYDRYDLGKADLNAFTRFEVKIGENTLTIKVNDEQKVNVDVSYWQHLLSYFKAGVYNQFENGEAKVQFKQLGLTKTDHTDSIAWNIDDWKLTIPTSKNDWYGFGGDSATELEPERCNSSKDPLSNEESVYQREIDLSYFNVIDGSMHFRADMGYGTSTANSSYIRSELRELYISTNSPDCSTSDEETSWYIEDSRTGATSHTLNATLRINEYPKIDGQLPKVVVGQIHGWKISQALVKLLWEGDNKPVRVILNDNYKLDNNKDCTDCNAFSVKLGTYAVNEDWQYTIRADKEGLFLATYDADGSNMVSHTLKWGEAYSDTANNKSYTLTERWASPDIAFYFKAGIYPQFKPDNAYRGEIFDVSFSALSTLHQ |
| 36 | WP_394622615.1 polysaccharide lyase family 7 protein [Vibrio alginolyticus] | MFIKSTKLLLISFSGLGLIGCGGNTSPTSQDDKPNIPHQEHVAPYSIAKFQNILSNSDLQVSDPNGKEGNKTSDVKNGAFSDYKSDHFYAEKNSNYLVFKMSNYKMRSEVRERENFNISEQGVSRTLYADVRLPEINLAMASSPANHDEVTFLQIHNKGTDTSGIGAIPHPLLRIVWEQERNSITGHYWAVVKNNAIDCSLPSSASDCYATSYDRYDLGKADLNAFTRFEVKIGENTLTIKVNDEQKVNVDVSYWQHLLSYFKAGVYNQFENGEAKVQFKQLGLTKTDHTDSIAWNIDDWKLTIPASKNDWYGFGGDSAAELEPERCNSSKDPLSNEESVYQREIDLSYFNVIDGSMHFRADMGYGTSTANSSYIRSELRELYISTNSPDCSTSDEETSWYIEDSRTGATSHTLNATLRINEYPKIDGQLPKVVVGQIHGWKISQALVKLLWEGDNKPVRVILNDNYKLDNNKDCTDCNAFSVKLGTYAVNEDWQYTIRADKEGLFLATYDADGSNMVSHTLKWGEAYSDTANNKSYTLTERWASPDIAFYFKAGIYPQFKPDNAYRGEIFDVSFSALSTLHQ |
| 37 | EGQ8041452.1 polysaccharide lyase family 7 protein [Vibrio alginolyticus] | MFIKSTKLLLISFSGLGLIGCGGNTSPTSQDDKPNIPHQEHVAPYSIAKFQNILSNSDLQVSDPNGKEGNKTSDVKNGAFSDYKSDHFYAEKDSNYLVFKMSNYKMRSEVRERENFNISEQGVSRTLYADVRLPEINLAMASSPANHDEVTFLQIHNKGTDTSATGAIPHPLLRIVWEQERNSITGHYWAVVKNNAIDCSLPSSASDCYATSYDRYDLGKADLKAFTRFEVKIGENTLTIKVNDEQKVNVDVSYWQHLLSYFKAGVYNQFENGEAKVQFKQLGLTKTDHTDSIAWNIDDWKLTIPASKNDWYGFGGDSAAELEPERCNSSKDPLSNEESVYQREIDLSYFNVIDGSMHFRADMGYGTSTANSSYIRSELRELYISTNSPDCSTSDEETSWYIEDSRTGATSHTLNATLRINEYPKIDGQLPKVVVGQIHGWKISQALVKLLWEGDNKPVRVILNDNYKLDNNKDCTDCNAFSVKLGTYAVNEDWQYTIRADKEGLFLATYDADGSNMVSHTLKWGEAYSDTANNKSYTLTERWASPDIAFYFKAGIYPQFKPDNAYRGEIFDVSFSALSTLHQ |
| 38 | WP_238975469.1 polysaccharide lyase family 7 protein [Vibrio alginolyticus] | MFIKSTKLLLISFSGLGLIGCGGNTSPTSQDDKPNIPHQEHVAPYSIAKFQNILSNSDLQVSDPNGKEGNKTSDVKNGAFSDYKSDHFYAEKDSNYLVFKMSNYKMRSEVRKRENFNISEQGVSRTLYADVRLPEINLAMASSPANHDEVTFLQIHNKGTDTSGTGAIPHPLLRIVWEQERNSITGHYWAVVKNNAIDCSLPSSASDCYATSYDRYDLGKADLNAFTRFEVKIGENTLTIKVNDEQKVNVDVSYWQHLLSYFKAGVYNQFENGEAKVQFKQLGLTKTDHTDSIAWNIDDWKLTIPTSKNDWYGFGGDSAAELEPERCNSSKDPLSNEESVYQREIDLSYFNVIDGSMHFRADMGYGTSTANSSYIRSELRELYISTNSPDCSTSDEETSWYIEDSRTGATSHTLNATLRINEYPKIDGQLPKVVVGQIHGWKISQALVKLLWEGDNKPVRVILNDNYKLDNNKDCTDCNAFSVKLGTYAVNEDWQYTIRADKEGLFLATYDADGSNMVSHTLKWGEAYSDTANNKSYTLTERWASPDIAFYFKAGMYPQFKPDNAYRGEIFDVSFSALSTLHQ |
| 39 | WP_258478320.1 polysaccharide lyase family 7 protein [Vibrio alginolyticus] | MFIKSTKLLLISFSGLGLIGCGGNTATTSQDDKPNIPHQEHVAPYSIAKFQNILSNSDLQVSDPNGEEGNKNSDVKNGAFSDYKSDHFYAEKDSNYLVFKMSNYKMRSEVRERENFNISEQGVFRTLYADVRLPEINLAMASSPANHDEVTFLQIHNKGTDTSGIGAIPHPLLRIVWEQERNSITGHYWAVVKNNAIDCSLPSSASDCYATSYDRYDLGKADLNAFTRFEVKIGENTLTIKVNDEQKVNVDVSYWQHLLSYFKAGVYNQFENGEAKVQFKQLGLTKTDHTDSIAWNIDDWKLTIPASKNDWYGFGGDSAAELEPERCNSSKDPLSNEESVYQREIDLSYFNVIDGSMHFRADMGYGTSTANSSYIRSELRELYISTNSPDCSTSDEETSWYIEDSRTGATSHTLNATLRINEYPKIDGQLPKVVVGQIHGWKISQALVKLLWEGDNKPVRVILNDNYKLDNNKDCTDCNAFSVKLGTYAVNEDWQYTIRADKEGLFLATYDADGSNMVSHTLKWGEAYSDTANNKSYTLTERWASPDIAFYFKAGIYPQFKPDNAYRGEIFDVSFSALSTLHQ |
| 40 | ENN2390205.1 polysaccharide lyase family 7 protein [Vibrio alginolyticus] | MFIKSTKLLLISFSGLGLIGCGGNTAPTSQDDKPNIPHQEHVAPYSIAKFQNILSNSDLQVSDPNGKEGNKTSDVKNGAFSDYKSDHFYAEKDSNYLVFKMSNYKMRSEVRERENFNISEQGVFRTLYADVRLPEINLAMASSPANHDEVTFLQIHNKGTDTSGTGAIPHPLLRIVWEQERNSITGHYWAVVKNNSIDCSLPSSASDCYATSYDRYDLGKADLNAFTRFEVKIGENTLTIKVNDEQKVNVDVSYWQHLLSYFKAGVYNQFENGEAKVQFKQLGLTKTDHTDSIAWNIDDWKLTIPASKNDWYGFGGDSAAELEPERCNSSKDPLSNEESVYQREIDLSYFNVIDGSMHFRADMGYGTSTANSSYIRSELRELYISTNSPDCSTSDEETSWYIEDSRTGATSHTLNATLRINEYPKIDGQLPKVVVGQIHGWKISQALVKLLWEGDNKPVRVILNDNYKLDNNKDCTDCNAFSVKLGTYAVNEDWQYTIRADKEGLFLATYDADGSNMVSHTLRWGEAYSDTANNKSYTLTERWASPDIAFYFKAGIYPQFKPDNAYRGEIFDVSFSALSTLHQ |
| 41 | WP_258483455.1 polysaccharide lyase family 7 protein [Vibrio alginolyticus] | MFIKSTKLLLISFSGLGLIGCGGNTATTSQDDKPNIPHQEHVAPYSIAKFQNILSNSDLQVSDPNGEEGNKNSDVKNGAFSDYKSDHFYAENDSNYLVFKMSNYKMRSEVRERENFNISEQGVSRTLYADVRLPEINLAMASSPANHDEVTFLQIHNKGTDTSGIGAIPHPLLRIVWEQERNSITGHYWAVVKNNAIDCSLPSSASDCYATSYDRYDLGKADLNAFTRFEVKIGENTLTIKVNDEQKVNVDVSYWQHLLSYFKAGVYNQFENGEAKVQFKQLGLTKTDHTDSIAWNIDDWKLTIPASKNDWYGFGGDSAAELEPERCNSSKDPLSNEESVYQREIDLSYFNVIDGSMHFRADMGYGTSTANSSYIRSELRELYISTNSPDCSTSDEETSWYIEDSRTGATSHTLNATLRINEYPKIDGQLPKVVVGQIHGWKISQALVKLLWEGDNKPVRVILNDNYKLDNNKDCTDCNAFSVKLGTYAVNEDWQYTIRADKEGLFLATYDADGSNMVSHTLKWGEAYSDTANNKSYTLTERWASPDIAFYFKAGIYPQFKPDNAYRGEIFDVSFSALSTLHQ |
| 42 | ENM8379249.1 polysaccharide lyase family 7 protein [Vibrio alginolyticus] | MFIKSTKLLLISFSGLGLIGCGGNTATTSQDDKPNIPHQEHVAPYSIAKFQNILRNSDLQVSDPDGKEGNKTSDVKNGAFSDYKSDHFYAEKDSNYLVFKMSNYKMRSEVRERENFNISEQGVFRTLYADVRLPEINLAMASSPANHDEVTFLQIHNKGTDTSGIGAIPHPLLRIVWEQERNSITGHYWAVVKNNAIDCSLPSSASDCYATSYNRYDLGKADLNAFTRFEVKIGENTLTIKVNDEQKVNVDVSYWQHLLSYFKAGVYNQFENGEAKVQFKQLGLTKTDHTDSIAWNIDDWKLTIPASKNDWYGFGGDSAAELEPERCNSSKDLLSNEESVYQREIDLSYFNVIDGSMHFRADMGYGTSTANSSYIRSELRELYISTNSPDCSTSDEETSWYIEDSRTGATSHTLNATLRINEYPKIDGQFPKVVVGQIHGWKISQALVKLLWEGDNKPVRVILNDNYKLDNNKDCTDCNAFSVKLGTYAVNEDWQYTIRADKEGLFLATYDADGSNMVSHTLKWGEAYSDTANNKSYTLTERWASPDIAFYFKAGIYPQFKPDNAYRGEIFDVSFSALSTLHQ |
| 43 | WP_334227307.1 polysaccharide lyase family 7 protein [Vibrio alginolyticus] | MFIKSTKLLLISFSGLGLIGCGGNTSPTSQDDKPNIPHQEHVAPYSIAKFQNILSNSDLQVSDPNGKEGNKTSDVKNGAFSDYKSDHFYAEKDSNYLVFKMSNYKMRSEVRERENFNISEQGVSRTLYADVRLPEINLAMASSPANHDEVTFLQIHNKGTDTSGTGAIPHPLLRIVWEQERNSITGHYWAVVKNNAIDCSLPSSASDCYATSYDRYDLGKADLNAFTRFEVKIGENTLTIKVNDEQKVNVDVSYWQHLLSYFKAGVYNQFENGEAKVQFKQLGLTKTDHTDSIAWNIDDWKLTIPASKNDWYGFGGDSAAELEPERCNSSKDLLSNEESVYQREIDLSYFNVIDGSMHFRADMGYGTSTANSSYIRSELRELYISTNSPDCSTSDEETSWYIEDSRTGATSHTLNATLRINEYPKIDGQLPKVVVGQIHGWKISQALVKLLWEGDNKPVRVILNDNYKLDNNKDCTDCNAFSVKLGTYAVNEDWQYTIRADKEGLFLATYDADGSNMVSHTLKWGEAYSDTANNKSYTLTERWASPDIAFYFKAGIYPQFKPDNAYRGEIFDVSFSALSTLHQ |
| 44 | EJG0479084.1 polysaccharide lyase family 7 protein [Vibrio alginolyticus] | MFIKSTKLLLISFSGLGLIGCGGNTATTSQDDKPNIPHQEHVAPYSIAKFQNILRNSDLQVSDPDGKEGNKTSDVKNGAFSDYKSDHFYAEKDSNYLVFKMSNYKMRSEVRERENFNISEQGVSRTLYADVRLPEINLAMASSPANHDEVTFLKIHNKGTDTSGTGAIPHPLLRIVWEQERNSITGHYWAVVKNNAIDCSLPSSASDCYATSYDRYDLGKAALNAFTRFEVKIGENTLTIKVNDEQKVNVDVSYWQHLLSYFKAGVYNQFENGEAKVQFKQLGLTKTDHTDSIAWNIDDWKLTIPTSKNDWYGFGGDSAAELEPERCNSSKDLLSNEESAYQREIDLSYFNVIDGSMHFRADMGYGTSTANSSYIRSELRELYISTNSPDCSTSDEETSWYIEDSRTGATSHTLNATLRINEYPKIDGQLPKVVVGQIHGWKISQALVKLLWEGDNKPVRVILNDNYKLDNNKDCTDCNAFSVKLGTYAVNEDWQYTIRADKEGLFLASYDADGSNMVSHTLKWGEAYSDTANNKSYTLTERWASPDIAFYFKAGIYPQFKPDNAYRGEIFDVSFSALSTLHQ |
| 45 | WP_170893276.1 polysaccharide lyase family 7 protein [unclassified Vibrio] | MFIKSTKLLLISFSGLGLIGCGGNTSPTSQDDKPNIPHQEHVAPYSIAKFQNILSNSDLQVSDPNGKEGNKTSDVKNGAFSDYKSDHFYAEKDSNYLVFKMSNYKMRSEVRERENFNISEQGVSRTLYADVRLPEINLAIASSPANHDEVTFLQIHNKGTDTSGTGAIPHPLLRIVWEQERNSITGHYWAVVKNNAIDCSLPSSASDCYATSYDRYDLGKADLNAFTRFEVKIGENTLTIKVNDEQKVNVDVSYWQHLLSYFKAGVYNQFENGEAKVQFKQLGLTKTDHTDSIAWNIDDWKLTIPTSKNDWYGFGGDSAAELEPERCNSSKDPLSNEESVYQREIDLSYFNVIDGSMHFRADMGYGTSTANSSYIRSELRELYISTNSPDCSTSDEETSWYIEDSRTGATSHTLNATLRINEYPKIDGQLPKVVVGQIHGWKISQALVKLLWEGDNKPVRVILNDNYKLDNNKDCTDCNAFSVKLGTYAVNEDWQYTIRADKEGLFLASYDADGSNMVSHTLKWGEAYSDTANNKSYTLTERWASPDIAFYFKAGIYPQFKPDNAYRGEIFDVSFSALSTLHQ |
| 46 | MFH4510214.1 polysaccharide lyase family 7 protein [Vibrio alginolyticus] | MFIKSTKLLLISFSGLGLIGCGGNTSPTSQDDKPNIPHQEHVAPYSIAKFQNILSNSDLQVSDPNGKEGNKTSDVKNGAFSDYKSDHFYAEKDSNYLVFKMSNYKMRSEVRERENFNISEQGVSRTLYADVRLPEINLAIASSPANHDEVTFLQIHNKGTDTSGTGAIPHPLLRIVWEQERNSITGHYWAVVKNNAIDCSLPSSASDCYATSYDRYDLGKADLNAFTRFEVKIGENTLTIKVNDEQKVNVDVSYWQHLLSYFKAGVYNQFENGEAKVQFKQLGLTKTDHTDSIAWNIDDWKLTIPTSKNDWYGFGGDSAAELEPERCNSSKDPLSNEESVYQREIDLSYFNVIDGSMHFRADMGYGTSTANSSYIRSELRELYISTNSPDCSTSDEETSWYIEDSRTGATSHTLNATLRINEYPKIDGQLPKVVVGQIHGWKISQALVKLLWEGDNKPVRVILNDNYKLDNNKDCTDCNAFSVKLGTYAVNEDWQYTIRADKEGLFLATYDADGSNMVSHTLKWGEAYSDTSNNKSYTLTERWASPDIAFYFKAGIYPQFKPDNAYRGEIFDVSFSALSTLHQ |
| 47 | WP_395228253.1 polysaccharide lyase family 7 protein [Vibrio alginolyticus] | MFIKSTKLLLISFSGLGLIGCGGNTATTSQDDKPNIPHQEHVAPYSIAKFQNILSNSDLQVSDPNGKEGNKTSDVKNGAFSDYKSDHFYAENDSNYLVFKMSNYKMRSEVRERENFNISEQGVFRTLYADVRLPEINLAMASSPANHDEVTFLQIHNKGTDTSGTGAIPHPLLRIVWEQERNSITGHYWAVVKNNAIDCSLPSSASDCYATSYDRYDLGKADLNAFTRFEVKIGENTLTIKVNDEQKVNVDVSYWQHLLSYFKAGVYNQFENGEAKVQFKQLGLTKTDHTDSIAWNIDDWKLTIPTSKNDWYGFGGDSAAELEPERCNSSKDLLSNEESVYQREIDLSYFNVIDGSMHFRADMGYGTSTANSSYIRSELRELYISTNSPDCSTSDEETSWYIEDSRTGATSHTLNATLRINEYPKIDGQLPKVVVGQIHGWKISQALVKLLWEGDNKPVRVILNDNYKLDNNKDCTDCNAFSVKLGTYAVNEDWQYTIRADKEGLFLATYDADGSNMVSHTLKWGEAYSDTANNKSYTLTERWASPDIAFYFKAGIYPQFKPDNAYRGEIFDVSFSALSTLHQ |
| 48 | WP_193837726.1 polysaccharide lyase family 7 protein [unclassified Vibrio] | MFIKSTKLLLISFSGLGLIGCGGNTSPTSQDDKPNIPHQEHVAPYSIAKFQNILSNSDLQVSDPNGKEGNKTSDVKNGAFSDYKSDHFYAEKDSNYLVFKMSNYKMRSEVRERENFNISEQGVSRTLYADVRLPEINLAMASSPANHDEVTFLQIHNKGTDTSGTGAIPHPLLRIVWEQERNSITGHYWAVVKNNAIDCSLPSSASDCYATSYDRYDLGKADLNAFTRFEVKIGENTLTIKVNDEQKVNVDVSYWQHLLSYFKAGVYNQFENGEAKVQFKQLGLTETDHTDSIAWNIDDWKLTIPTSKNDWYGFGGDSATELEPERCNSSKDPLSNEESVYQREIDLSYFNVIDGSMHFRADMGYGTSTANSSYIRSELRELYISTNSPDCSTSDEETSWYIEDSRTGATSHTLNATLRINEYPKIDGQLPKVVVGQIHGWKISQALVKLLWEGDNKPVRVILNDNYKLDNNKDCTDCNAFSVKLGTYAVNEDWQYTIRADKEGLFLATYDADGSNMVSHTLKWGEAYSDTANNKSYTLTERWASPDIAFYFKAGIYPQFKPDNAYRGEIFDVSFSALSTLHQ |
| 49 | ELA6778130.1 polysaccharide lyase family 7 protein [Vibrio alginolyticus] | MFIKSTKLLLISFSGLGLIGCGGNTSPTSQDDKPNIPHQEHVAPYSIAKFQNILSNSDLQVSDPNGKEGNKTSDVKNGAFSDYKSDHFYAEKDSNYLVFKMSNYKMRSEVRERENFNISEQGVSRTLYADVRLPEINLAMASSPANHDEVTFLQIHNKGTDTSGTGAIPHPLLRIVWEQERNSITGHYWAVVKNNAIDCSLPSSASDCYATSYDRYDLGKADLNAFMRFEVKIGENTLTIKVNDEQKVNVDVSYWQHLLSYFKAGVYNQFENGEAKVQFKQLGLTKTDHTDSIAWNIDDWKLTIPASKNDWYGFGGDSAAELEPERCNSSKDPLSNEESVYQREIDLSYFNVIDGSMHFRADMGYGTSTANSSYIRSELRELYISTNSPDCSTSDEETSWYIEDSRTGATSHTLNATLRINEHPKIDGQFPKVVVGQIHGWKISQALVKLLWEGDNKPVRVILNDNYKLDNNKDCTDCNAFSVKLGTYAVNEDWQYTIRADKEGLFLATYDADGSNMVSHTLKWGEAYSDTANNKSYTLTERWASPDIAFYFKAGIYPQFKPDNAYRGEIFDVSFSALSTLHQ |
| 50 | WP_334458389.1 polysaccharide lyase family 7 protein [Vibrio alginolyticus] | MFIKSTKLLLISFSGLGLIGCGGNTSPTSQDDKPNIPHQEHVAPYSIAKFQNILSNSDLQVSDPNGEEGNKNSDVKNGAFSDYKSDHFYTEKDSNYLVFKMSNYKMRSEVRERENFNISEQGVSRTLYADVRLPEINLAMASSPANHDEVTFLQIHNKGTDTSGTGAIPHPLLRIVWEQERNSITGHYWAVVKNNAIDCSLPSSASDCYATSYDRYDLGKADLNAFTRFEVKIGENTLTIKVNDEQKVNVDVSYWQHLLSYFKAGVYNQFENGEAKVQFKQLGLTKTDHTDSIAWNIDDWKLTIPASKNDWYGFGGDSAAELEPERCNSSKDPLSNEESVYQREIDLSYFNVIDGSMHFRADMGYGTSTANSSYIRSELRELYISTNSPDCSTSDEETSWYIEDSRTGATSHTLNATLRINEYPKIDGQLPKVVVGQIHGWKISQALVKLLWEGDNKPVRVILNDNYKLDNNKDCTDCNAFSVKLGTYAVNEDWQYTIRADKEGLFLATYDADGSNMVSHTLKWGEAYSDTANNKSYTLTERWASPDIAFYFKAGIYPQFKPDNAYRGEIFDVSFSALSTLHQ |
| 51 | WP_318131838.1 polysaccharide lyase family 7 protein [Vibrio sp. Vb2960] | MFIKSTKLLLISFSGLGLIGCGGNTSPTSQDDKPNIPHQEHVAPYSIAKFQNILSNSDLQVSDPNGKEGNKTSDVKNGAFSDYKSDHFYAEKDSNYLVFKMSNYKMRSEVRERENFNISEQGVSRTLYADVRLPEINLAIASSPANHDEVTFLQIHNKGTDTSGTGAIPHPLLRIVWEQERNSITGHYWAVVKNNAIDCSLPSSASDCYATSYDRYDLGKADLNAFTRFEVKIGENTLTIKVNDEQKVNVDVSYWQHLLSYFKAGVYNQFENGEAKVQFKQLGLTKTDHTDSIAWNIDDWKLTIPTSKNDWYGFGGDSAAELEPERCNSSKDPLSNEESVYQREIDLSYFNVIDGSMHFRADMGYGTSTANSSYIRSELRELYISTNSPDCSTSDEETSWYIEDSRTGATSHTLNATLRINEYPKIDGQLPKVVVGQIHGWKISQALVKLLWEGDNKPVRVILNDNYKLDNNKDCTDCNAFSVKLGTYAVNEDWQYTIRADKKGLFLASYDADGSNMVSHTLKWGEAYSDTANNKSYTLTERWASPDIAFYFKAGIYPQFKPDNAYRGEIFDVSFSALSTLHQ |
| 52 | WP_137282565.1 polysaccharide lyase family 7 protein [Vibrio] | MFIKSTKLLLISFSGLGLIGCGGNTSPTSQDDKPNIPHQEHVAPYSIAKFQNILSNSDLQVSDPNGKEGNKTSDVKNGAFSDYKSDHFYAEKDSNYLVFKMSNYKMRSEVRERENFNISEQGVSRTLYADVRLPEINLAMASSPANHDEVTFLQIHNKGTDTSGTGAIPHPLLRIVWEQERNSITGHYWAVVKNNAIDCSLPSSASDCYATSYDRYDLGKADLNAFTRFEVKIGENTLTIKVNDEQKVNVDVSYWQHLLSYFKAGVYNQFENGEAKVQFNQLGLTKTDHTDSIAWNIDDWKLTIPASKNDWYGFGGDSAAELEPERCNSSKDLLSNEESVYQREIDLSYFNVIDGSMHFRADMGYGTSTANSSYIRSELRELYISTNSPDCSTSDEETSWYIEDSRTGATSHTLNATLRINEYPKIDGQLPKVVVGQIHGWKISQALVKLLWEGDNKPVRVILNDNYKLDNNKDCTDCNAFSVKLGTYAVNEDWQYTIRADKEGLFLASYDADGSNMVSHTLKWGEAYSDTANNKSYTLTERWASPDIAFYFKAGIYPQFKPDNAYRGEIFDVSFSALSTLHQ |
| 53 | WP_318072663.1 polysaccharide lyase family 7 protein [Vibrio sp. Vb1337] | MFIKSTKLLLISFSGLGLIGCGGNTSPTSQDDKPNIPHQEHVAPYSIAKFQNILSNSDLQVSDPNGKEGNKTSDVKNGAFSDYKSDHFYAEKDSNYLVFKMSNYKMRSEVRERENFNISEQGVSRTLYADVRLPEINLAMASSPANHDEVTFLQIHNKGTDTSGTGAIPHPLLRIVWEQERNSITGHYWAVVKNNAIDCSLPSSASDCYATSYDRYDLGKADLNAFMRFEVKIGENTLTIKVNDEQKVDVDVSYWQHLLSYFKAGVYNQFENGEAKVQFKQLGLTKTDHTDSIAWNIDDWKLTIPASKNDWYGFGGDSAAELEPERCNSSKDPLSNEESVYQREIDLSYFNVIDGSMHFRADMGYGTSTANSSYIRSELRELYISTNSPDCSTSDEETSWYIEDSRTGATSHTLNATLRINEHPKIDGQFPKVVVGQIHGWKISQALVKLLWEGDNKPVRVILNDNYKLDNNKDCTDCNAFSVKLGTYAVNEDWQYTIRADKEGLFLATYDADGSNMVSHTLKWGEAYSDTANNKSYTLTERWASPDIAFYFKAGIYPQFKPDNAYRGEIFDVSFSALSTLHQ |
| 54 | WP_225496200.1 polysaccharide lyase family 7 protein [Vibrio] | MFIKSTKLLLISFSGLGLIGCGGNTAPTSQDDKPNIPHQEHVAPYSIAKFQNILSNSDLQVSDPNGKEGNKTSDVKNGAFSDYKSDHFYAEKDSNYLVFKMSNYKMRSEVRERENFNISEQGVFRTLYADVRLPEINLAMASSPANHDEVTFLQIHNKGTDTSGTGAIPHPLLRIVWEQERNSITGHYWAVVKNNAIDCSLPSSASDCYATSYDRYDLGKADLNAFTRFEVKIGENTLTIKVNDEQKVNVDVSYWQHLLSYFKAGVYNQFENGEAKVQFKQLGLTKTDHTDSIAWNIDDWKLTIPTSKNDWYGFGGDSAAELEPERCNSSKDLLSNEESVYQREIDLSYFNVIDGSMHFRADMGYGTSTANSSYIRSELRELYISTNSPDCSTSDEETSWYIEDSRTGATSHTLNATLRINEYPKIDGQLPKVVVGQIHGWKISQALVKLLWEGDNKPVRVILNDNYKLDNNKDCTDCNAFSVKLGTYAVNEDWQYTIRADKEGLFLATYDADGSNMVSHTLKWGEAYSDTANNKSYTLTERWASPDIAFYFKAGIYPQFKPDNAYRGEIFDVSFSALSTLHQ |
| 55 | WP_172563859.1 polysaccharide lyase family 7 protein [Vibrio alginolyticus] | MFIKSTKLLLISFSGLGLIGCGGNTSPTSQDDKPNIPHQEHVAPYSIAKFQNILSNSDLQVSDPNGKEGNKTSDVKNGAFSDYKSDHFYAEKDSNYLVFKMSNYKMRSEVRERENFNISEQGVSRTLYADVRLPEINLAMASSPANHDEVTFLQIHNKGTDTSATGAIPHPLLRIVWEQERNSITGHYWAVVKNNAIDCSLPSSASDCYATSYDRYDLGKADLNAFTRFEVKIGENTLTIKVNDEQKVNVDVSYWQHLLSYFKAGVYNQFENGEAKVQFKQLGLTKTDHTDSIAWNIDDWKLTIPASKNDWYGFGGDSAAELEPERCNSSKDLLSNEESVYQREIDLSYFNVIDGSMHFRADMGYGTSTANSSYIRSELRELYISTNSPDCSTSDEETSWYIEDSRTGATSHTLNATLRINEYPKIDGQLPKVVVGQIHGWKISQALVKLLWEGDNKPVRVILNDNYKLDNNKDCTDCNAFSVKLGTYAVNEDWQYTIRADKEGLFLASYDADGSNMVSHTLKWGEAYSDTANNKSYTLTERWASPDIAFYFKAGIYPQFKPDNAYRGEIFDVSFSALSTLHQ |
| 56 | WP_318132380.1 polysaccharide lyase family 7 protein [Vibrio sp. 378] | MFIKSTKLLLISFSGLGLIGCGGNTSPTSQDDKPNIPHQEHVAPYSIAKFQNILSNSDLQVSDPNGKEGNKTSDVKNGALSDYKSDHFYAEKDSNYLVFKMSNYKMRSEVRERENFNISEQGVSRTLYADVRLPEINLAIASSPANHDEVTFLQIHNKGTDTSGTGAIPHPLLRIVWEQERNSITGHYWAVVKNNAIDCSLPSSASDCYATSYDRYDLGKADLNAFTRFEVKIGENTLTIKVNDEQKVNVDVSYWQHLLSYFKAGVYNQFENGEAKVQFKQLGLTKTDHTDSIAWNIDDWKLTIPTSKNDWYGFGGDSAAELEPERCNSSKDPLSNEESVYQREIDLSYFNVIDGSMHFRADMGYGTSTANSSYIRSELRELYISTNSPDCSTSDEETSWYIEDSRTGATSHTLNATLRINEYPKIDGQLPKVVVGQIHGWKISQALVKLLWEGDNKPVRVILNDNYKLDNNKDCTDCNAFSVKLGTYAVNEDWQYTIRADKEGLFLASYDADGSNMVSHTLKWGEAYSDTANNKSYTLTERWASPDIAFYFKAGIYPQFKPDNAYRGEIFDVSFSALSTLHQ |
| 57 | WP_171342558.1 polysaccharide lyase family 7 protein [Vibrio] | MFIKSTKLLLISFSGLGLIGCGGNTSPTRQDDKPNIPHQEHVAPYSIAKFQNILRNSDLQVSDPDGKEGNKTSDVKNGAFSDYKSDHFYAEKDSNYLVFKMSNYKMRSEVRERENFNISEQGVFRTLYADVRLPEINLAMASSPANHDEVTFLQIHNKGTDTSGTGAIPHPLLRIVWEQERNSITGHYWAVVKNNAIDCSLPSSASDCYATSYDRYDLGKADLNAFTRFEVKIGENTLTIKVNDEQKVNVDVSYWQHLLSYFKAGVYNQFENGEAKVQFKQLGLTKTDHTDSIAWNIDDWKLTIPASKNDWYGFGGDSAAELEPERCNSSKDLLSNEESVYQREIDLSYFNVIDGSMHFRADMGYGTSTANSSYIRSELRELYISTNSPDCSTSDEETSWYIEDSRTGATSHTLNATLRINEYPKIDGQLPKVVVGQIHGWKISQALVKLLWEGENKPVRVILNDNYKLDNNKDCTDCNAFSVKLGTYAVNEDWQYTIRADKEGLFLATYDADGSNMVSHTLKWGEAYSDTANNKSYTLTERWASPDIAFYFKAGIYPQFKPDNAYRGEIFDVSFSVLSTLHQ |
| 58 | EKZ9009616.1 polysaccharide lyase family 7 protein [Vibrio alginolyticus] | MFIKSTKLLLISFSGLGLIGCGGNTSPTSQDDKPNIPHQEHVAPYSIAKFQNILSNSDLQVSDPNGKEGNKTSDVKNGAFSDYKSDHFYAEKDSNYLVFKMSNYKMRSEVRERENFNISEQGVSRTLYADVRLPEINFAMASSPANHDEVTFLQIHNKGTDTSGTGAIPHPLLRIVWEQERNSITGHYWAVVKNNAIDCSLPSSASDCYATSYDRYDLGKADLNAFMRFEVKIGENTLTIKVNDEQKVNVDVSYWQHLLSYFKAGVYNQFENGEAKVQFKQLGLTKTDHTDSIAWNIDDWKLTIPASKNDWYGFGGDSAAELEPERCNSSKDPLSNEESVYQREIDLSYFNVIDGSMHFRADMGYGTSTANSSYIRSELRELYISTNSPDCSTSDEETSWYIEDSRTGATSHTLNATLRINEHPKIDGQFPKVVVGQIHGWKISQALVKLLWEGDNKPVRVILNDNYKLDNNKDCTDCNAFSVKLGTYAVNEDWQYTIRADKEGLFLATYDADGSNMISHTLKWGEAYSDTANNKSYTLTERWASPDIAFYFKAGIYPQFKPDNAYRGEIFDVSFSALSTLHQ |
| 59 | WP_372430169.1 polysaccharide lyase family 7 protein [Vibrio alginolyticus] | MFIKSTKLLLISFSGLGLIGCGGNTSPTSQDDKPNIPHQEHVAPYSIAKFQNILSNSDLQVSDPNGKEGNKTSDVKNGAFSDYKSDHFYAEKDSNYLVFKMSNYKMRSEVRERENFNISEQGVSRTLYADVRLPEINLAMASSPANHDEVTFLQIHNKGTDTSGTGAIPHPLLRIVWEQERNSITDHYWAVVKNNAIDCSLPSSASDCYATSYDRYDLGKADLNAFTRFEVKIGENTLTIKVNDEQKVNVDVSYWQHLLSYFKAGVYNQFENGEAKVQFKQLGLTKTDHTDSIAWNIDDWKLTIPASKNDWYGFGGDSAAELEPERCNSSKDPLSNEESVYQREIDLSYFNVIDGSMHFRADMGYGTSTANSSYIRSELRELYISTNSPDCSTSDEETSWYIEDSRTGATSHTLNATLRINEYPKIDGQLPKVVVGQIHGWKISQALVKLLGEGDNKPVRVILNDNYKLDNNKDCTDCNAFSVKLGTYAVNEDWQYTIRADKEGLFLATYDADGSNMVSHTLKWGEAYSDTANNKSYTLTERWASPDIAFYFKAGIYPQFKPDNAYRGEIFDVSFSALSTLHQ |
| 60 | HCZ9266521.1 polysaccharide lyase family 7 protein [Vibrio alginolyticus] | MFIKSTKLLLISFSGLGLIGCGGNTSPTSQDDKPNIPHQEHVAPYSIAKFQNILSNSDLQVSDPNGKEGNKNSDVKNGAFSDYKSDHFYAENDSNYLVFKMSNYKMRSEVRERENFNISEQGVFRTLYADVRLPEINLAMASSPANHDEVTFLQIHNKGTDTSGTGAIPHPLLRIVWEQERNSITGHYWAVVKNNAIDCSLPSSASDCYATSYDRYDLGKADLNAFTRFEVKIGENTLTIKVNDEQKVNVDVSYWQHLLSYFKAGVYNQFENGEAKVQFKQLGLTKTDHTDSIAWNIDDWKLTIPASKNDWYGFGGDSAAELEPERCNSSKDPLSNEESVYQREIDLSYFNVIDGSMHFRADMGYGTSTANSSYIRSELRELYISTNSPDCSTSDEETSWYIEDSRTGATSHTLNATLRINEYPKIDGQLPKVVVGQIHGWKISQALVKLLWEGDNKPVRVILNDNYKLDNNKDCTDCNAFSVKLGTYAVNEDWQYTIRADKEGLFLASYDADGSNMVSHTLKWGEAYSDTANNKSYTLTERWASPDIAFYFKAGIYPQFKPDNAYRGEIFDVSFSALSTLHQ |
| 61 | WP_373921419.1 polysaccharide lyase family 7 protein [Vibrio alginolyticus] | MKSTKLLLISFSGLGLIGCGGNTSPTSQDDKPNIPHQEHVAPYSIAKFQNILSNSDLQVSDPNGKEGNKTSDVKNGAFSDYKSDHFYAEKDSNYLVFKMSNYKMRSEVRERENFNISEQGVSRTLYADVRLPEINLAMASSPANHDEVTFLQIHNKGTDTSGTGAIPHPLLRIVWEQERNSITDHYWAVVKNNAIDCSLPSSASDCYATSYDRYDLGKADLNAFTRFEVKIGENTLTIKVNDEQKVNVDVSYWQHLLSYFKAGVYNQFENGEAKVQFKQLGLTKTDHTDSIAWNIDDWKLTIPASKNDWYGFGGDSAAELEPERCNSSKDPLSNEESVYQREIDLSYFNVIDGSMHFRADMGYGTSTANSSYIRSELRELYISTNSPDCSTSDEETSWYIEDSRTGATSHTLNATLRINEYPKIDGQLPKVVVGQIHGWKISQALVKLLWEGDNKPVRVILNDNYKLDNNKDCTDCNAFSVKLGTYAVNEDWQYTIRADKEGLFLATYDADGSNMVSHTLKWGEAYSDTANNKSYTLTERWASPDIAFYFKAGIYPQFKPDNAYRGEIFDVSFSALSTLHQ |
| 62 | MBS9931934.1 polysaccharide lyase family 7 protein [Vibrio alginolyticus] | MLLISFSGLGLIGCGGNTATTSQDDKPNIPHQEHVAPYSIAKFQNILRNSDLQVSDPDGKEGNKTSDVKNGAFSDYKSDHFYAEKDSNYLVFKMSNYKMRSEVRERENFNISEQGVSRTLYADVRLPEINLAMASSPANHDEVTFLQIHNKGTDTSGTGAIPHPLLRIVWEQERNSITGHYWAVVKNNAIDCSLPSSASDCYATSYDRYDLGKADLNAFTRFEVKIGENTLTIKVNDKQKVNVDVSYWQHLLSYFKAGVYNQFENGEAKVQFKQLGLTKTDHTDSIAWNIDDWKLTIPASKNDWYGFGGDSAAELEPERCNSSKDPLSNEESVYQREIDLSYFNVIDGSMHFRADMGYGTSTANSSYIRSELRELYISTNSPDCSTSDEETSWYIEDSRTGATSHTLNATLRINEYPKIDGQLPKVVVGQIHGWKISQALVKLLWEGDNKPVRVILNDNYKLDNNKDCTDCNAFSVKLGTYAVNEDWQYTIRADKEGLFLATYDADGSNMISHTLKWGEAYSDTANNKSYTLTERWASPDIAFYFKAGIYPQFKPDNAYRGEIFDVSFSALSTLHQ |
| 63 | WP_104980754.1 polysaccharide lyase family 7 protein [Vibrio alginolyticus] | MFIKSTKLLLISFSGLGLIGCGGNTSPTSQDDKPNIPHQEHVAPYSIAKFQNILSNSDLQVSDPNGKEGNKTSDVKNGAFSDYKSDHFYAEKDSNYLVFKMSNYKMRSEVRERENFNISEQGVFRTLYADVRLPEINLAMASSPANHDEVTFLQIHNKGTDTSGTGAIPHPLLRIVWEQERNSITGHYWAVVKNNAIDCSLPSSASDCYATSYDRYDLGKADLNAFTRFEVKIGENTLTIKVNDEQKVNVDVSYWQHLLSYFKAGVYNQFENGEAKVQFKQLGLTKTDHTDSIAWNIDDWKLTIPTSKNDWYGFGGDSAAELEPERCNSSKDLLSNEESVYQREIDLSYFNVIDGSMHFRADMGYGTSTANSSYIRSELRELYISTNSPDCSTSDEETSWYIEDSRTGATSHTLNATLRINEYPKIDGQLPKVVVGQIHGWKISQALVKLLWEGDNKPVRVILNDNYKLDNNKDCTDCNAFSVKLGTYAVNEDWQYTIRADKEGLFLASYDADGSNMVSHTLKWGEAYSDTANNKSYTLTERWASPDIAFYFKAGIYPQFKPDNAYRGEIFDVSFSALSTLHQ |
| 64 | WP_053349965.1 polysaccharide lyase family 7 protein [Vibrio] | MFIKSTKLLLISFSGLGLIGCGGNTSPTSQDDKPNIPHQEHVAPYSIAKFQNILSNSDLQVSDPNGEEGNKNSDVKNGAFSDYKSDHFYAEKDSNYLVFKMSNYKMRSEVRERENFNISEQGVFRTLYADVRLPEINLAMASSPANHDEVTFLQIHNKGTDTSGIGAIPHPLLRIVWEQERNSITGHYWAVVKNNAIDCSLPSSASDCYATSYDRYDLGKADLNAFTRFEVKIGENTLTIKVNDEQKVNVDVSYWQHLLSYFKAGVYNQFENGEAKVQFKQLGLTKTDHTDSIAWNIDDWKLTIPASKNDWYGFGGDSAAELEPERCNSSKDPLSNEESVYQREIDLSYFNVIDGSMHFRADMGYGTSTANSSYIRSELRELYISTNSPDCSTSDEETSWYIEDSRTGATSHTLNATLRINEYPKIDGQLPKVVVGQIHGWKISQALVKLLWEGDNKPVRVILNDNYKLDNNKDCTDCNAFSVKLGTYAVNEDWQYTIRADKEGLFLATYDADGSNMVSHTLKWGEAYSDTANNKSYTLTERWASPDIAFYFKAGIYPQFKPDNAYRGEIFDVSFSALSTLHQ |
| 65 | EIP0121015.1 polysaccharide lyase family 7 protein [Vibrio alginolyticus] | MFIKSTKLLLISFSGLGLIGCGGNTATTSQDDKPNIPHQEHVAPYSIAKFQNILSNSDLQVSDPNGKEGNKTSDVKNGAFSDYKSDHFYAEKDSNYLVFKMSNYKMRSEVRERENFNISEQGVFRTLYADVRLPEINLAMASSPANHDEVTFLQIHNKGTDTSGTGAIPHSLLRIVWEQERNSITGHYWAVVKNNAIDCSLPSSASDCYATSYDRYDLGKADLNAFTRFEVKIGENTLTIKVNDEQKVNVDVSYWQHLLSYFKAGVYNQFENGEAKVQFKQLGLTKTDHTDSIAWNIDDWKLTIPTSKNDWYGFGGDSAAELEPERCNSSKDLLSNEESVYQREIDLSYFNVIDGSMHFRADMGYGTSTANSSYIRSELRELYISTNSPDCSTSDEETSWYIEDSRTGATSHTLNATLRINEYPKIDGLLPKVVVGQIHGWKISQALVKLLWEGDNKPVRVILNDNYKLDNNKDCTDCNAFSVKLGTYAVNEDWQYTIRADKEGLFLATYDADGSNMVSHTLKWGEAYSDTANNKSYTLTERWASPDIAFYFKAGIYPQFKPDNAYRGEIFDVSFSALSTLHQ |
| 66 | WP_213891276.1 polysaccharide lyase family 7 protein [Vibrio alginolyticus] | MFIKSTKLLLISFSGLGLIGCGGNTSPTSQDDKPNIPHQEHVAPYSIAKFQNILSNSDLQVSDPNGEEGNKNSDVKNGAFSDYKSDHFYAENDSNYLVFKMSNYKMRSEVRERENFNISEQGVFRTLYADVRLPEINLAMASSPANHDEVTFLQIHNKGTDTSGTGAIPHPLLRIVWEQERNSITGHYWAVVKNNAIDCSLPSSASDCYATSYDRYDLGKADLNAFTRFEVKIGENTLTIKVNDEQKVNVDVSYWQHLLSYFKAGVYNQFENGEAKVQFKQLGLTKTDHTDSIAWNIDDWKLTIPTSKNDWYGFGGDSAAELEPERCNSSKDPLSNEESVYQREIDLSYFNVIDGSMHFRADMGYGTSTANSSYIRSELRELYISTNSPDCSTSDEETSWYIEDSRTGATSHTLNATLRINEYPKIDGQLPKVVVGQIHGWKISQALVKLLWEGDNKPVRVILNDNYKLDNNKDCTDCNAFSVKLGTYAVNEDWQYTIRADKEGLFLATYDADGSNMVSHTLKWGEAYSDTANNKSYTLTERWASPDIAFYFKAGIYPQFKPDNAYRGEIFDVSFSALSTLHQ |
| 67 | WP_258655078.1 polysaccharide lyase family 7 protein [Vibrio alginolyticus] | MFIKSTKLLLISFSGLGLIGCGGNTSPTSQDDKPNIPHQEHVAPYSIAKFQNILSNSDLQVSDPNGEEGNKNSDVKNGAFSDYKSDHFYAEKDSNYLVFKMSNYKMRSEVRERENFNISEQGVFRTLYADVRLPEINLAMASSPANHDEVTFLQIHNKGTDTSGTGAIPHPLLRIVWEQERNSITGHYWAVVKNNAIDCSLPSSASDCYATSYVRYDLGKADLNAFTRFEVKIGENTLTIKVNDEQKVNVDVSYWQHLLSYFKAGVYNQFENGEAKVQFKQLGLTKTDHTDSIAWNIDDWKLTIPASKNDWYGFGGDSAAELEPERCNSSKDPLSNEESVYQREIDLSYFNVIDGSMHFRADMGYGTSTANSSYIRSELRELYISTNSPDCSTSDEETSWYIEDSRTGATSHTLNATLRINEYPKIDGQLPKVVVGQIHGWKISQALVKLLWEGDNKPVRVILNDNYKLDNNKDCTDCNAFSVKLGTYAVNEDWQYTIRADKEGLFLATYDADGSNMVSHTLKWGEAYSDTANNKSYTLTERWASPDIAFYFKAGIYPQFKPDNAYRGEIFDVSFSALSTLHQ |
| 68 | WP_085568976.1 polysaccharide lyase family 7 protein [Vibrio] | MFIKSTKLLLISFSGLGLIGCGGNTAPTSQDDKSNIPHQEHVAPYSIAKFQNILSNSDLQVSDPNGKEGNKTSDVKNGAFSDYKSDHFYAEKDSNYLVFKMSNYKMRSEVRERENFNISEQGVFRTLYADVRLPEINLAMASSPANHDEVTFLQIHNKGTDTSGTGAIPHPLLRIVWEQERNSITGHYWAVVKNNAIDCSLPSSASDCYATSYDRYDLGKADLKAFTRFEVKIGENTLTIKVNDEQKVNVDVSYWQHLLSYFKAGVYNQFENGEAKVQFKQLGLTKTDHTDSIAWNIDDWKLTIPASKNDWYGFGGDSAAELEPERCNSSKDLLSNEESVYQREIDLSYFNVIDGSMHFRADMGYGTSTANSSYIRSELRELYISTNSPDCSTSDEETSWYIEDSRTGATSHTLNATLRINEYPKIDGQLPKVVVGQIHGWKISQALVKLLWEGDNKPVRVILNDNYKLDNNKDCTDCNAFSVKLGTYAVNEDWQYTIRADKEGLFLATYDADGSNMVSHTLKWGEAYSDTANNKSYTLTERWASPDIAFYFKAGIYPQFKPDNAYRGEIFDVSFSALSTLHQ |
| 69 | WP_054576768.1 polysaccharide lyase family 7 protein [Vibrio] | MFIKSTKLLLISFSGLGLIGCGGNTSPTSQDDKPNIPHQEHVAPYSVAKFQNILSNSDLQVSDPNGEEGNKNSDVKNGAFSDYKSDHFYAEKDSNYLVFKMSNYKMRSEVRERENFNISEQGVFRTLYADVRLPEINLAMASSPANHDEVTFLQIHNKGTDTSGIGAIPHPLLRIVWEQERNSITGHYWAVVKNNAIDCSLPSSASDCYATSYDRYDLGKADLNAFTRFEVKIGENTLTIKVNDEQKVNVDVSYWQHLLSYFKAGVYNQFENGEAKVQFKQLGLTKTDHTDSIAWNIDDWKLTIPASKNDWYGFGGDSAAELEPERCNSSKDPLSNEESVYQREIDLSYFNVIDGSMHFRADMGYGTSTANSSYIRSELRELYISTNSPDCSTSDEETSWYIEDSRTGATSHTLNATLRINEYPKIDGQLPKVVVGQIHGWKISQALVKLLWEGDNKPVRVILNDNYKLDNNKDCTDCNAFSVKLGTYAVNEDWQYTIRADKEGLFLATYDADGSNMVSHTLKWGEAYSDTANNKSYTLTERWASPDIAFYFKAGIYPQFKPDNAYRGEIFDVSFSALSTLHQ |
| 70 | WP_213962406.1 polysaccharide lyase family 7 protein [Vibrio alginolyticus] | MFIKSTKLLLISFSGLGLIGCGGNTSPTSQDDKPNIPHQEHVAPYSIAKFQNILSNSDLQVSNPNGEEGNKNSDVKNGAFSDYKSDHFYAEKDSNYLVFKMSNYKMRSEVRERENFNISEQGVFRTLYADVRLPEINLAMASSPANHDEVTFLQIHNKGTDTSGIGAIPHPLLRIVWEQERNSITGHYWAVVKNNAIDCSLPSSASDCYATSYDRYDLGKADLNAFTRFEVKIGENTLTIKVNDEQKVNVDVSYWQHLLSYFKAGVYNQFENGEAKVQFKQLGLTKTDHTDSIAWNIDDWKLTIPASKNDWYGFGGDSAAELEPERCNSSKDPLSNEESVYQREIDLSYFNVIDGSMHFRADMGYGTSTANSSYIRSELRELYISTNSPDCSTSDEETSWYIEDSRTGATSHTLNATLRINEYPKIDGQLPKVVVGQIHGWKISQALVKLLWEGDNKPVRVILNDNYKLDNNKDCTDCNAFSVKLGTYAVNEDWQYTIRADKEGLFLATYDADGSNMVSHTLKWGEAYSDTANNKSYTLTERWASPDIAFYFKAGIYPQFKPDNAYRGEIFDVSFSALSTLHQ |
| 71 | WP_064369206.1 polysaccharide lyase family 7 protein [Vibrio] | MFIKSTKLLLISFSGLGLIGCGGNTSPTSQDDKPNIPHQEHVAPYSVAKFQNILSNSDLQVSDPNGEEGNKNSDVKNGAFSDYKSDHFYAEKDSNYLVFKMSNYKMRSEVRERENFNISEQGVFRTLYADVRLPEINLAMASSPANHDEVTFLQIHNKGTDTSGIGAIPHPLLRIVWEQERNSITGHYWAVVKNNAIDCSLPSSASDCYATSYDRYDLGKADLNAFTRFEVKIGENTLTIKVNDEQKVNVDVSYWQHLLSYFKAGVYNQFENGEAKVQFKQLGLTKTDHTDSIAWNIDDWKLTIPASKNEWYGFGGDSAAELEPERCNSSKDPLSNEESVYQREIDLSYFNVIDGSMHFRADMGYGTSTANSSYIRSELRELYISTNSPDCSTSDEETSWYIEDSRTGATSHTLNATLRINEYPKIDGQLPKVVVGQIHGWKISQALVKLLWEGDNKPVRVILNDNYKLDNNKDCTDCNAFSVKLGTYAVNEDWQYTIRADKEGLFLATYDADGSNMVSHTLKWGEAYSDTANNKSYTLTERWASPDIAFYFKAGIYPQFKPDNAYRGEIFDVSFSALSTLHQ |
| 72 | WP_262606497.1 polysaccharide lyase family 7 protein [Vibrio sp. 1641] | MFIKSTKLLLISFSGLGLIGCGGNTSPTSQDDKPNIPHQEHVAPYSIAKFQNILSNSDLQVSDPNGEEGNKNSDVKNGAFSDYKSDHFYAEKDSNYLVFKMSNYKMRSEARERENFNISEQGVFRTLYADVRLPEINLAMASSPANHDEVTFLQIHNKGTDTSGIGAIPHPLLRIVWEQERNSITGHYWAVVKNNAIDCSLPSSASDCYATSYDRYDLGKADLNAFTRFEVKIGENTLTIKVNDEQKVNVDVSYWQHLLSYFKAGVYNQFENGEAKVQFKQLGLTKTDHTDSIAWNIDDWKLTIPASKNDWYGFGGDSAAELEPERCNSSKDPLSNEESVYQREIDLSYFNVIDGSMHFRADMGYGTSTANSSYIRSELRELYISTNSPDCSTSDEETSWYIEDSRTGATSHTLNATLRINEYPKIDGQLPKVVVGQIHGWKISQALVKLLWEGDNKPVRVILNDNYKLDNNKDCTDCNAFSVKLGTYAVNEDWQYTIRADKEGLFLATYDADGSNMVSHTLKWGEAYSDTANNKSYTLTERWASPDIAFYFKAGIYPQFKPDNAYRGEIFDVSFSALSTLHQ |
| 73 | WP_318146183.1 polysaccharide lyase family 7 protein [Vibrio sp. 1731] | MFIKSTKLLLISFSGLGLIGCGGNTSPTRQDDKPNIPHQEHVAPYSIAKFQNILRNSDLQVSDPDGKEGNKTSDVKNGAFSDYKSDHFYAEKDSNYLVFKMSNYKMRSEVRERENFNISEQGVFRTLYADVRLPEINLAMASSPANHDEVTFLQIHNKGTDTSGTGAIPHPLLRIVWEQERNSITGHYWAVVKNNAIDCSLPSSASDCYATSYDRYDLGKADLNAFTRFEVKIGENTLTIKVNDEQKVNVDVSYWQHLLSYFKAGVYNQFENGEAKVQFKQLGLTKTDHTDSIAWNIDDWKLTIPASKNDWYGFGGDSAAELEPERCNSSKDLLSNEESVYQREIDLSYFNVIDGSMHFRADMGYGTSTANSSYIRSELRELYISTNSPDCSTSDEETSWYIEDSRTGATSHTLNATLRINEYPKIDGQLPKVVVGQIHGWKISQALVKLLWEGENKPVRVILNDNYKLDNNKDCTDCNAFSVKLGTYAVNEDWQYTIRADKEGLFLATYDAYGSNMVSHTLKWGEAYSDTANNKSYTLTERWASPDIAFYFKAGIYPQFKPDNAYRGEIFDVSFSVLSTLHQ |
| 74 | WP_213874000.1 polysaccharide lyase family 7 protein [Vibrio alginolyticus] | MFIKSTKLLLISFSGLGLIGCGGNTSPTSQDDKPNIPHQEHVAPYSIAKFQNILSNSDLQVSDPNGKEGNKTSDVKNGAFSDYKSDHFYAEKDSNYLVFKMSNYKMRSEVRERENFNISEQGVSRTLYADVRLPEINLAMASSPANHDEVTFLQIHNKGTDTSATGAIPHPLLRIVWEQERNSITGHYWAVVKNNAIDCSLPSSASDCYATSYDRYDLGKADLKAFTRFEVKIGENTLTIKVNDEQKVNVDVSYWQHLLSYFKAGVYNQFENGEAKVQFKQLGLTKTDHTDSIAWNIDDWKLTIPTSKNDWYGFGGDSAAELEPERCNSSKDLLSNEESVYQREIDLSYFNVIDGSMHFRADMGYGTSTANSSYIRSELRELYISTNSPDCSTSDEETSWYIEDSRTGATSHTLNATLRINEYPKIDGQLPKVVVGQIHGWKISQALVKLLWEGDNKPVRVILNDNYKLDNNKDCTDCNAFSVKLGTYAVNEDWQYTIRADKEGLFLASYDADGSNMVSHTLKWGEAYSDTANNKSYTLTERWASPDIAFYFKAGIYPQFKPDNAYRGEIFDVSFSALSTLHQ |
| 75 | EJL6924629.1 polysaccharide lyase family 7 protein [Vibrio alginolyticus] | MFIKSTKLLLISFSGLGLIGCGGNTSPTSQDDKPNIPHQEHVAPYSIAKFQNILSNSDLQVSDPNGEEGNKNSDVKNGAFSDYKSDHFYAEKDSNYLVFKMSNYKMRSEVRERENFNISEQGVSRTLYADVRLPEINLAMASSPANHDEVTFLQIHNKGTDTSGTGAIPHPLLRIVWEQERNSITGHYWAVVKNNAIDCSLPSSASDCYATSYDRYDLGKADLNAFTRFEVKIGENTLTIKVNDEQKVNVDVSYWQHLLSYFKAGVYNQFENGEAKVQFNQLGLTKTDHTDSIAWNIDDWKLTIPASKNDWYGFGGDSAAELEPERCNSSKDLLSNEESVYQREIDLSYFNVIDGSMHFRADMGYGTSTANSSYIRSELRELYISTNSPDCSTSDEETSWYIEDSRTGATSHTLNATLRINEYPKIDGQLPKVVVGQIHGWKISQALVKLLWEGDNKPVRVILNDNYKLDNNKDCTDCNAFSVKLGTYAVNEDWQYTIRADKEGLFLASYDADGSNMVSHTLKWGEAYSDTANNKSYTLTERWASPDIAFYFKAGIYPQFKPDNAYRGEIFDVSFSALSTLHQ |
| 76 | WP_225473156.1 polysaccharide lyase family 7 protein [Vibrio] | MFIKSTKLLLISFSGLGLIGCGGNTSPTSQDDKPNIPHQEHVAPYSIAKFQNILSNSDLQVSDPNGEEGNKNSDVKNGAFSDYKSDHFYAEKDSNYLVFKMSNYKMRSEVRERENFNISEQGVFRTLYADVRLPEINLAMASSPANHDEVTFLQIHNKGTDTSGIGAIPHPLLRIVWEQERNSITGHYWAVVKNNAIDCSLPSSASDCYATSYDRYDLGKADLNAFTRFEVKIGENTLTIKVNDEQKVNVDVSYWQHLLSYFKAGVYNQFENGEAKVQFKQLGLTKTDHTDSIAWNIDDWKLTIPASKNDWYGFGGDSAAELEPERCNSSKDPLSNEESVYQRDIDLSYFNVIDGSMHFRADMGYGTSTANSSYIRSELRELYISTNSPDCSTSDEETSWYIEDSRTGATSHTLNATLRINEYPKIDGQLPKVVVGQIHGWKISQALVKLLWEGDNKPVRVILNDNYKLDNNKDCTDCNAFSVKLGTYAVNEDWQYTIRADKEGLFLATYDADGSNMVSHTLKWGEAYSDTANNKSYTLTERWASPDIAFYFKAGIYPQFKPDNAYRGEIFDVSFSALSTLHQ |
| 77 | WP_258460774.1 polysaccharide lyase family 7 protein [Vibrio] | MFIKSTKLLLISFSGLGLIGCGGNTSPTSQDDKPNIPHQEHVAPYSIAKFQNILSNSDLQVSDPNGEEGNKNSDVKNGAFSDYKSDHFYAEKDSNYLVFKMSNYKMRSEVRERENFNISEQGVFRTLYADVRLPEINLAMASSPANHDEVTFLQIHNKGTDTSGIGAIPHPLLRIVWEQERNSITGHYWAVVKNNAIDCSLPSSASDCYATSYDRYDLGKADLNAFTRFEVKIGENTLTIKVNDEQKVNVDVSYWQHLLSYFKAGVYNQFENGEAKVQFKQLGLTKTDHTDSIAWNIDDWKLTIPASKNDWYGFGGDSAAELEPERCNSSKDPLSNEESVYQREIDLSYFNVIDGSMHFRADMGYGTSTANSSYIRSELRELYISTNSPDCSTSDEETSWYIEDSRTGATSHTLNATLRINEYPKIDGQLPKVVVGQIHGWKISQALVKLLWEGDNKPVRVILNDNYKLDNNKDCTDCNAFSVKLGTYAVNEDWQYTIRADKEGLFLATYDADGSNMASHTLKWGEAYSDTANNKSYTLTERWASPDIAFYFKAGIYPQFKPDNAYRGEIFDVSFSALSTLHQ |
| 78 | WP_334423651.1 polysaccharide lyase family 7 protein [Vibrio alginolyticus] | MFIKSTKLLLIIFSGLGLIGCGGNTSPTSQDDKPNIPHQEHVAPYSIAKFQNILSNSDLQVSDPNGEEGNKNSDVKNGAFSDYKSDHFYAEKDSNYLVFKMSNYKMRSEVRERENFNISEQGVFRTLYADVRLPEINLAMASSPANHDEVTFLQIHNKGTDTSGIGAIPHPLLRIVWEQERNSITGHYWAVVKNNAIDCSLPSSASDCYATSYDRYDLGKADLNAFTRFEVKIGENTLTIKVNDEQKVNVDVSYWQHLLSYFKAGVYNQFENGEAKVQFKQLGLTKTDHTDSIAWNIDDWKLTIPASKNDWYGFGGDSAAELEPERCNSSKDPLSNEESVYQREIDLSYFNVIDGSMHFRADMGYGTSTANSSYIRSELRELYISTNSPDCSTSDEETSWYIEDSRTGATSHTLNATLRINEYPKIDGQLPKVVVGQIHGWKISQALVKLLWEGDNKPVRVILNDNYKLDNNKDCTDCNAFSVKLGTYAVNEDWQYTIRADKEGLFLATYDADGSNMVSHTLKWGEAYSDTANNKSYTLTERWASPDIAFYFKAGIYPQFKPDNAYRGEIFDVSFSALSTLHQ |
| 79 | WP_213876140.1 polysaccharide lyase family 7 protein [Vibrio alginolyticus] | MFIKSTKLLLISFSGLGLIGCGGNTSPTSQDDKPNIPHQEHVAPYSIAKFQNILSNSDLQVSDPNGEEGNKNSDVKNGAFSDYKSDHFYAEKDSNYLVFKMSNYKMRSEVRERENFNISEQGVFRTLYADVRLPEINLAMTSSPANHDEVTFLQIHNKGTDTSGIGAIPHPLLRIVWEQERNSITGHYWAVVKNNAIDCSLPSSASDCYATSYDRYDLGKADLNAFTRFEVKIGENTLTIKVNDEQKVNVDVSYWQHLLSYFKAGVYNQFENGEAKVQFKQLGLTKTDHTDSIAWNIDDWKLTIPASKNDWYGFGGDSAAELEPERCNSSKDPLSNEESVYQREIDLSYFNVIDGSMHFRADMGYGTSTANSSYIRSELRELYISTNSPDCSTSDEETSWYIEDSRTGATSHTLNATLRINEYPKIDGQLPKVVVGQIHGWKISQALVKLLWEGDNKPVRVILNDNYKLDNNKDCTDCNAFSVKLGTYAVNEDWQYTIRADKEGLFLATYDADGSNMVSHTLKWGEAYSDTANNKSYTLTERWASPDIAFYFKAGIYPQFKPDNAYRGEMFDVSFSALSTLHQ |
| 80 | WP_258473690.1 polysaccharide lyase family 7 protein [Vibrio alginolyticus] | MFIKSTKLLLISFSGLGLIGCGGNTSPTSQDDKPNIPHQEHVAPYSIAKFQNILSNSDLQVSDPNGKEGNKTSDVKNGAFSDYKSDHFYAENDSNYLVFKMSNYKMRSEVRERENFNISEQGVFRTLYADVRLPEINLAMASSPANHDEVTFLQIHNKGTDTSGTGAIPHPLLRIVWEQERNSITGHYWAVVKNNAIDCSLPSSASDCYATSYDRYDLGKADLNAFTRFEVKIGENTLTIKVNDEQKVNVDVSYWQHLLSYFKAGVYNQFENGEAKVQFKQLGLTKTDHTDSIAWNIDDWKLTIPTSKNDWYGFGGDSAAELEPERCNSSKDLLSNEESVYQREIDLSYFNVIDGSMHFRADMGYGTSTANSSYIRSELRELYISTNSPDCSTSDEETSWYIEDSRTGATSHTLNATLRINEYPKIDGQLPKVVVGQIHGWKISQALVKLLWEGDNKPVRVILNDNYKLDNNKDCTDCNAFSVKLGTYAVNEDWQYTIRADKEGLFLASYDADGSNMVSHTLKWGEAYSDTANNKSYTLTERWASPDIAFYFKAGIYPQFKPDNAYRGEIFDVSFSALSTLHQ |
| 81 | EGQ8016093.1 polysaccharide lyase family 7 protein [Vibrio alginolyticus] | MFIKSTKLLLISFSGLGLIGCGGNTSPTSQDDKPNIPHQEHVAPYSIAKFQNILSNSDLQVSDPNGEEGNKNSDVKNGAFSDYKSDHFYAEKDSNYLVFKMSNYKMRSEVRERENFNISEQGVFRTLYADVRLPEINLAMASSPANHDEVTFLQIHNKGTDTSGIGAIPHPLLRIVWEQERNSITGHYWAVVKNNAIDCSLPSSASDCYATSYDRYDLGKADLNAFTRFEVKIGENTLTIKVNDEQKVNVDVSYWQHLLSYFKAGVYNQFENGEAKVQFKQLGLTKTDHTDSIAWNIDDWKLTIPASKNDWYGFGGDSAAELEPERCNSSKDPLSNEESVYQREIDLSYFNVIDGSMHFRADMGYGTSTANSSYIRSELRELYISTNSPDCSTSDEETSWYIEDSRTGATSHTLNARLRINEYPKIDGQLPKVVVGQIHGWKISQALVKLLWEGDNKPVRVILNDNYKLDNNKDCTDCNAFSVKLGTYAVNEDWQYTIRADKEGLFLATYDADGSNMVSHTLKWGEAYSDTANNKSYTLTERWASPDIAFYFKAGIYPQFKPDNAYRGEIFDVSFSALSTLHQ |
| 82 | WP_136976431.1 polysaccharide lyase family 7 protein [Vibrio alginolyticus] | MFIKSTKLLLISFSGLGLIGCGGNTSPTSQDDKPNIPHQEHVAPYSIAKFQNILSNSDLQVSDPNGEEGNKNSDVKNGAFSDYKSDHFYAEKDSNYLVFKMSNYKMRSEVRERENFNISEQGVFRTLYADVRLPEINLAMASSPANHDEVTFLQIHNKGTDTSGIGAIPHPLLRIVWEQERNSITGHYWAVVKNNAIDCSLPSSASDCYATSYDRYDLGKADLNAFTRFEVKIGENTLTIKVNDEQKVNVDVSYWQHLLSYFKAGVYNQFENGEAKVQFKQLGLTKTDHTDSIAWNIDDWKLTIPASKNDWYGFGGDSAAELEPERCNSSKDPLSNEESVYQREIDLSYFNVIDGSMHFRADMGYGTSTANSSYIRSELRELYISTNSPDCSTSDEETSWYIEDSRTGATSHTLNATLRINEYPKIDGQLPKVVVGQIHGWKISQALVKLLWEGDNKPVRVILNDNYKLDNNKDCTDCNAFSVKLGTYAVNEDWQYTIRADKEGLFLATYDAGGSNMVSHTLKWGEAYSDTANNKSYTLTERWASPDIAFYFKAGIYPQFKPDNAYRGEIFDVSFSALSTLHQ |
| 83 | EMG1954340.1 polysaccharide lyase family 7 protein [Vibrio alginolyticus] | MFIKSTKLLLISFSGLGLIGCGGNTSPTSQDDKPNIPHQEHVAPYSIAKFQNILSNSDLQVSDPNGEEGNKNSDVKNGAFSDYKSDHFYAEKDSNYLVFKMSNYKMRSEVRERENFNISEQGVFRTLYADVRLPEINLAMAGSPANHDEVTFLQIHNKGTDTSGIGAIPHPLLRIVWEQERNSITGHYWAVVKNNAIDCSLPSSASDCYATSYDRYDLGKADLNAFTRFEVKIGENTLTIKVNDEQKVNVDVSYWQHLLSYFKAGVYNQFENGEAKVQFKQLGLTKTDHTDSIAWNIDDWKLTIPASKNDWYGFGGDSAAELEPERCNSSKDPLSNEESVYQREIDLSYFNVIDGSMHFRADMGYGTSTANSSYIRSELRELYISTNSPDCSTSDEETSWYIEDSRTGATSHTLNATLRINEYPKIDGQLPKVVVGQIHGWKISQALVKLLWEGDNKPVRVILNDNYKLDNNKDCTDCNAFSVKLGTYAVNEDWQYTIRTDKEGLFLATYDADGSNMVSHTLKWGEAYSDTANNKSYTLTERWASPDIAFYFKAGIYPQFKPDNAYRGEIFDVSFSALSTLHQ |
| 84 | WP_213867771.1 polysaccharide lyase family 7 protein [Vibrio alginolyticus] | MFIKSTKLLLISFSGLGLIGCGGNTSPTSQDDKPNIPHQEHVAPYSIAKFQNILSNSDLQVSDPNGEEGNKNSDVKNGAFSDYKSDHFYAEKDSNYLVFKMSNYKMRSEVRERENFNISEQGFFRTLYADVRLPEINLAMASSPANHDEVTFLQIHNKGTDTSGIGAIPHPLLRIVWEQERNSITGHYWAVVKNNAIDCSLPSSASDCYATSYDRYDLGKADLNAFTRFEVKIGENTLTIKVNDEQKVNVDVSYWQHLLSYFKAGVYNQFENGEAKVQFKQLGLTKTDHTDSIAWNIDDWKLTIPASKNDWYGFGGDSAAELEPERCNSSKDPLSNEESVYQREIDLSYFNVIDGSMHFRADMGYGTSTANSSYIRSELRELYISTNSPDCSTSDEETSWYIEDSRTGATSHTLNATLRINEYPKIDGQLPKVVVGQIHGWKISQALVKLLWEGDNKPVRVILNDNYKLDNNKDCTDCNAFSVKLGTYAVNEDWQYTIRADKEGLFLATYDADGSNMVSHTLKWGEAYSDTANNKSYTLTERWASPDIAFYFKAGIYPQFKPDNAYRGEIFDVSFSALSTLHQ |
| 85 | WP_213968345.1 polysaccharide lyase family 7 protein [Vibrio alginolyticus] | MFIKSTKLLLISFSGLGLIGCGGNTAPTSQDDKPNIPHQEHVAPYSIAKFQNILSNSDLQVSDPNGKEGNKTSDVKNGAFSDYKSDHFYAEKDSNYLVFKMSNYKMRSEVRERENFNISEQGVFRTLYADVRLPEINLAMASSPANHDEVTFLQIHNKGTDTSGTGAIPHPLLRIVWEQERNSITGHYWAVVKNNAIDCSLPSSASDCYATSYDRYDLGKADLNAFTRFEVKIGENTLTIKVNDEQKVNVDVSYWQHLLSYFKAGVYNQFENGEAKVQFKQLGLTKTDHTDSIAWNIDDWKLTIPTSKNDWYGFGGDSAAELEPERCNSSKDLLSNEESVYQREIDLSYFNVIDGSMHFRADMGYGTSTANSSYIRSELRELYISTNSPDCSTSDEETSWYIEDSRTGATSHTLNATLRINEYPKIDGQLPKVVVGQIHGWKISQALVKLLWEGDNKPVRVILNDNYKLDNNKDCTDCNAFSVKLGTYAVNEDWQYTIRADKEGLFLATYDADGSNMVSRTLKWGEAYSDTANNKPYTLTERWASPDIAFYFKAGIYPQFKPDNAYRGEIFDVSFSALSTLHQ |
| 86 | WP_171344077.1 polysaccharide lyase family 7 protein [Vibrio alginolyticus] | MFIKSTKLLLISFSGLGLIGCGGNTAPTSQDDKSNIPHQEHVAPYSIAKFQNILSNSDLQVSDPNGKEGNKTSDVKNGAFSDYKSDHFYAEKDSNYLVFKMSNYKMRSEVRERENFNISEQGVFRTLYADVRLPEINLAMASSPANHDEVTFLQIHNKGTDTSGTGAIPHPLLRIVWEQERNSITGHYWAVVKNNAIDCSLPSSASDCYATSYDRYDLGKADLKAFTRFEVKIGENTLTIKVNDEQKVNVDVSYWQHLLSYFKAGVYNQFENGEAKVQFKQLGLTKTDHTDSIAWNIDDWKLTIPASKNDWYGFGGDSAAELEPERCNSSKDLLSNEESVYQREIDLSYFNVIDGSMHFRADMGYGTSTANSSYIRSELRELYISTNSPDCSTSDEETSWYIEDSRTGATSHTLNATLRINEYPKIDGQLPKVVVGQIHGWKISQALVKLLWEGDNKPVRVILNDNYKLDNNKDCTDCNAFSVKLGTYAVNEDWQYTIRADKEGLFLATYDADGSNMVSHTLKWGEAHSDTANNKSYTLTERWASPDIAFYFKAGIYPQFKPDNAYRGEIFDVSFSALSTLHQ |
| 87 | WP_222711898.1 polysaccharide lyase family 7 protein [Vibrio alginolyticus] | MFIKSTKLLLISFSGLGLIGCGGNTAPTSQDDKSNIPHQEHVAPYSIAKFQNILSNSDLQVSDPNGKEGNKTSDVKSGAFSDYKSDHFYAEKDSNYLVFKMSNYKMRSEVRERENFNISEQGVFRTLYADVRLPEINLAMASSPANHDEVTFLQIHNKGTDTSGTGAIPHPLLRIVWEQERNSITGHYWAVVKNNAIDCSLPSSASDCYATSYDRYDLGKADLKAFTRFEVKIGENTLTIKVNDEQKVNVDVSYWQHLLSYFKAGVYNQFENGEAKVQFKQLGLTKTDHTDSIAWNIDDWKLTIPASKNDWYGFGGDSAAELEPERCNSSKDLLSNEESVYQREIDLSYFNVIDGSMHFRADMGYGTSTANSSYIRSELRELYISTNSPDCSTSDEETSWYIEDSRTGATSHTLNATLRINEYPKIDGQLPKVVVGQIHGWKISQALVKLLWEGDNKPVRVILNDNYKLDNNKDCTDCNAFSVKLGTYAVNEDWQYTIRADKEGLFLATYDADGSNMVSHTLKWGEAYSDTANNKSYTLTERWASPDIAFYFKAGIYPQFKPDNAYRGEIFDVSFSALSTLHQ |
| 88 | WP_021707800.1 polysaccharide lyase family 7 protein [Vibrio] | MFIKSTKLLLISFSGLGLIGCGGNTSPTSQDDKPNIPHQEHVAPYSIAKFQNILSNSDLQVSDPNGEEGNKNSDVKNGAFSDYKSDHFYAEKDSNYLVFKMSNYKMRSEVRERENFNISEQGVFRTLYADVRLPEINLAMASSPANHDEVTFLQIHNKGTDTSGIGAIPHPLLRIVWEQERNSITGHYWAVVKNNAIDCSLPSSASDCYATSYDRYDLGKADLNAFTRFEVKIGENTLTIKENDEQKVNVDVSYWQHLLSYFKAGVYNQFENGEAKVQFKQLGLTKTDHTDSIAWNIDDWKLTIPASKNDWYGFGGDSAAELEPERCNSSKDPLSNEESVYQREIDLSYFNVIDGSMHFRADMGYGTSTANSSYIRSELRELYISTNSPDCSTSDEETSWYIEDSRTGATSHTLNATLRINEYPKIDGQLPKVVVGQIHGWKISQALVKLLWEGDNKPVRVILNDNYKLDNNKDCTDCNAFSVKLGTYAVNEDWQYTIRADKEGLFLATYDADGSNMASHTLKWGEAYSDTANNKSYTLTERWASPDIAFYFKAGIYPQFKPDNAYRGEIFDVSFSALSTLHQ |
| 89 | EME9800584.1 polysaccharide lyase family 7 protein [Vibrio alginolyticus] | MFIKSTKLLLISFSGLGLIGCGGNTSPTSQDDKPNIPHQEHVAPYSIAKFQNILSNSDLQVSDPNGEEGNKNSDVKNGAFSDYKSDHFYAEKDSNYLVFKMSNYKMRSEVRERENFNISEQGVFRTLYADVRLPEINLAMAGSPANHDEVTFLQIHNKGTDTSGIGAIPHPLLRIVWEQERNSITGHYWAVVKNNAIDCSLPSSASDCYATSYDRYDLGKADLNAFTRFEVKIGENTLTIKVNDEQKVNIDVSYWQHLLSYFKAGVYNQFENGEAKVQFKQLGLTKTDHTDSIAWNIDDWKLTIPASKNDWYGFGGDSAAELEPERCNSSKDPLSNEESVYQREIDLSYFNVIDGSMHFRADMGYGTSTANSSYIRSELRELYISTNSPDCSTSDEETSWYIEDSRTGATSHTLNATLRINEYPKIDGQLPKVVVGQIHGWKISQALVKLLWEGDNKPVRVILNDNYKLDNNKDCTDCNAFSVKLGTYAVNEDWQYTIRTDKEGLFLATYDADGSNMVSHTLKWGEAYSDTANNKSYTLTERWASPDIAFYFKAGIYPQFKPDNAYRGEIFDVSFSALSTLHQ |
| 90 | WP_138940341.1 polysaccharide lyase family 7 protein [Vibrio alginolyticus] | MFIKSTKLLLISFSGLGLIGCGGNTSPTRQDDKPNIPHQEHVAPYSIAKFQNILRNSDLQVSDPDGKEGNKTSDVKNGAFSDYKSDHFYAEKDSNYLVFKMSNYKMRSEVRERENFNISEQGVSRTLYADVRLPEINLAMASSPANHDEVTFLQIHNKGTDTSGIGAIPHPLLRIVWEQERNSITGHYWAVVKNNAIDCSIPSSASDCYATSYDRYDLGKADLNAFTRFEVKIGENTLTIKVNDEQKVNVDVSYWQHLLSYFKAGVYNQFENGEAKVQFKQLGLTKTDHTDSIAWNIDDWKLTIPASKNDWYGFGGDSAAELEPERCNSSKDLLSNEESVLQSEIDLSYFNVIDGSMHFRADMGYGTSTANSSYIRSELRELYISTNSPDCSTSDEETSWYIEDSRTGATSHTLNATLRINEYPKIDGQLPKVVVGQIHGWKISQALVKLLWEGDNKPVRVILNDNYKLDNNKDCTDCNASSVKLGTYAVNEDWQYTIRADKEGLFLATYDADGSNMVSHTLKWGEAYSDTANNKSYTLTERWASPDIAFYFKAGIYPQFKPDNAYRGEIFDVSFSALSTLHQ |
| 91 | WP_262637947.1 polysaccharide lyase family 7 protein [Vibrio] | MFIKSTKLLLISFSGLGLIGCGGNTSPTSQDDKPNIPHQEHVAPYSIAKFQNILSNSDLQVSDPNGKEGNKTSDVKNGAFSDYKSDHFYAEKDSNYLVFKMSNYKMRSEVRERENFNISEQGVFRTLYADVRLPEINLTMASSPANHDEVTFLQIHNKGTDTSGTGAIPHPLLRIVWEQERNSITGHYWAVVKNNAIDCSLPSSASDCYATSYDRYDLGKADLKAFTRFEVKIGENTLTIKVNDEQKVNVDVSYWQHLLSYFKAGVYNQFENGEAKVQFKQLGLTKTDHTDSIAWNIDDWKLTIPTSKNDWYGFGGDSAAELEPERCNSSKDLLSNEESVYQREIDLSYFNVIDGSMHFRADMGYGTSTANSSYIRSELRELYISTNSPDCSTSDEETSWYIEDSRTGATSHTLNATLRINEYPKIDGQLPKVVVGQIHGWKISQALVKLLWEGDNKPVRVILNDNYKLDNNKDCTDCNAFSVKLGTYAVNEDWQYTIRADKEGLFLASYDADGSNMVSHTLKWGEAYSDTANNKSYTLTERWASPDIAFYFKAGIYPQFKPDNAYRGEIFDVSFSALSTLHQ |
| 92 | WP_213906677.1 polysaccharide lyase family 7 protein [Vibrio alginolyticus] | MFIKSTKLLLISFSGLGLIGCGGNTSPTSQDDKPNIPHQEHVAPYSVAKFQNILSNSDLQVSDPNGEEGNKNSDVKNGAFSDYKSDHFYAEKDSNYLVFKMSNYKMRSEVRERENFNISEQGVFRTLYADVRLPEINLAMASSPANHDEVTFLQIHNKGTDTSGIGAIPHPLLRIVWEQERNSITGHYWAVVKNNAIDCSLPSSASDCYATSYDRYDLGKADLNAFTRFEVKIGENTLTIKVNDEQKVNVDVSYWQHLLSYFKAGVYNQFENGEAKVQFKQLGLTKTDHTDSIAWNIDDWKLTIPASKNDWYGFGGDSAAELEPERCNSSKDPLSNEESVYQREIDLSYFNVIDGSMHFRADMGYGTSTANSSYIRSELRELYISTNSPDCSTSDEETSWYIEDSRTGATSHTLNATLRINEYPKIDGQLPKVVVGQIHGWKISQALVKLLWEGDNKPVRVILNDNYKLDNNKDCTDCNAFSVKLGTYAVNEDWQYTIRADKERLFLATYDADGSNMVSHTLKWGEAYSDTANNKSYTLTERWASPDIAFYFKAGIYPQFKPDNAYRGEIFDVSFSALSTLHQ |
| 93 | WP_395237952.1 polysaccharide lyase family 7 protein [Vibrio alginolyticus] | MFIKSTKLLLISFSGLGLIGCGGNTSPISQDDKPNIPHQEHVAPYSIAKFQNILSNSDLQVSDPNGEEGNKNSDVKNGAFSDYKSDHFYAEKDSNYLVFKMSNYKMRSEVRERENFNISEQGFFRTLYADVRLPEINLAMASSPANHDEVTFLQIHNKGTDTSGIGAIPHPLLRIVWEQERNSITGHYWAVVKNNAIDCSLPSSASDCYATSYDRYDLGKADLNAFTRFEVKIGENTLTIKVNDEQKVNVDVSYWQHLLSYFKAGVYNQFENGEAKVQFKQLGLTKTDHTDSIAWNIDDWKLTIPASKNDWYGFGGDSAAELEPERCNSSKDPLSNEESVYQREIDLSYFNVIDGSMHFRADMGYGTSTANSSYIRSELRELYISTNSPDCSTSDEETSWYIEDSRTGATSHTLNATLRINEYPKIDGQLPKVVVGQIHGWKISQALVKLLWEGDNKPVRVILNDNYKLDNNKDCTDCNAFSVKLGTYAVNEDWQYTIRADKEGLFLATYDADGSNMVSHTLKWGEAYSDTANNKSYTLTERWASPDIAFYFKAGIYPQFKPDNAYRGEIFDVSFSALSTLHQ |
| 94 | EJE8153245.1 polysaccharide lyase family 7 protein [Vibrio alginolyticus] | MFIKSTKLLLISFSGLGLIGCGGNTSPTSQDDKPNIPHQEHVAPYSIAKFQNILSNSDLQVSDPNGKEGNKNSDVKNGAFSDYKSDHFYAENDSNYLVFKMSNYKMRSEVRERENFNISEQGVFRTLYADVRLPEINLAMASSPANHDEVTFLQIHNKGTDTSGTGAIPHPLLRIVWEQERNSITGHYWAVVKNNAIDCSLPSSASDCYATSYDRYDLGKADLNAFTRFEVKIGENTLTIKVNDEQKVNVDVSYWQHLLSYFKAGVYNQFENGEAKVQFNQLGLTKTDHTDSIAWNIDDWKLTIPASKNDWYGFGGDSAAELEPERCNSSKDLLSNEESVYQREIDLSYFNVIDGSMHFRADMGYGTSTANSSYIRSELRELYISTNSPDCSTSDEETSWYIEDSRTGATSHTLNATLRINEYPKIDGQLPKVVVGQIHGWKISQALVKLLWEGDNKPVRVILNDNYKLDNNKDCTDCNAFSVKLGTYAVNEDWQYTIRADKEGLFLATYDADGSNMVSHTLKWGEAYSDTANNKSYTLTERWASPDIAFYFKAGIYPQFKPDNAYRGEIFDVSFSALSTLHQ |
| 95 | WP_258615864.1 polysaccharide lyase family 7 protein [Vibrio alginolyticus] | MFIKSTKLLLTSFSGLGLIGCGGNTSPTSQDDKPNIPHQEHVAPYSIAKFQNILSNSDLQVSDPNGEEGNKNSDVKNGAFSDYKSDHFYAEKDSNYLVFKMSNYKMRSEVRERENFNISEQGFFRTLYADVRLPEINLAMASSPANHDEVTFLQIHNKGTDTSGIGAIPHPLLRIVWEQERNSITGHYWAVVKNNAIDCSLPSSASDCYATSYDRYDLGKADLNAFTRFEVKIGENTLTIKVNDEQKVNVDVSYWQHLLSYFKAGVYNQFENGEAKVQFKQLGLTKTDHTDSIAWNIDDWKLTIPASKNDWYGFGGDSAAELEPERCNSSKDPLSNEESVYQREIDLSYFNVIDGSMHFRADMGYGTSTANSSYIRSELRELYISTNSPDCSTSDEETSWYIEDSRTGATSHTLNATLRINEYPKIDGQLPKVVVGQIHGWKISQALVKLLWEGDNKPVRVILNDNYKLDNNKDCTDCNAFSVKLGTYAVNEDWQYTIRADKEGLFLATYDADGSNMVSHTLKWGEAYSDTANNKSYTLTERWASPDIAFYFKAGIYPQFKPDNAYRGEIFDVSFSALSTLHQ |
| 96 | WP_158159371.1 polysaccharide lyase family 7 protein [Vibrio] | MFIKSTKLLLISFSGLGLIGCGGNTSPTSQDDKPNIPHQEHVAPYSIAKFQNILSNSDLQVSDPNGEEGNKNSDVKNGAFSDYKSDHFYAEKDSNYLVFKMSNYKMRSEVRERENFNISEQGVFRTLYADVRLPEINLAMASSPANHDEVTFLQIHNKGTDTSGIGAIPHPLLRIVWEQERNSITGHYWAVVKNNAIDCSLPSSASDCYATSYDRYDLGKADLNAFTRFEVKIGENTLTIKVNDEQKVNVDVSYWQHLLSYFKAGVYNQFENGEAKVQFKQLGLTKTDHTDSIAWNIDDWKLTIPASKNDWYGFGGDSAAELEPERCNSSKDPLSNEESVYQREIDLSYFNVIDGSMHFRADMGYGTSTANSSYIRSELRELDISTNSPDCSTSDEETSWYIEDSRTGATSHTLNATLRINEYPKIDGQLPKVVVGQIHGWKISQALVKLLWEGDNKPVRVILNDNYKLDNNKDCTDCNAFSVKLGTYAVNEDWQYTIRADKEGLFLATYDADGSNMVSHTLKWGEAYSDTANNKSYTLTERWASPDIAFYFKAGIYPQFKPDNAYRGEIFDVSFSALSTLHQ |
| 97 | WP_213903733.1 polysaccharide lyase family 7 protein [Vibrio alginolyticus] | MFIKSTKLLLISFSGLGLIGCGGNTSPTSQDDKPNIPHQEHVAPYSIAKFQNILSNSDLQVSDPNGKEGNKTSDVKNGAFSDYKSDHFYAENDSNYLVFKMSNYKMRSEVRERENFNISEQGVFRTLYADVRLPEINLAMASSPANHDEVTFLQIHNKGTDTSGIGAIPHPLLRIVWEQERNSITGHYWAVVKNNAIDCSLPSSASDCYATSYNRYDLGKADLNAFTRFEVKIGENTLTIKVNDEQKVNVDVSYWQHLLSYFKAGVYNQFENGEAKVQFKQLGLTKTDHTDSIAWNIDDWKLTIPASKNDWYGFGGDSAAELEPERCNSSKDLLSNEESVYQREIDLSYFNVIDGSMHFRADMGYGTSTANSSYIRSELRELYISTNSPDCSTSDEETSWYIEDSRTGATSHTLNATLRINEYPKIDGQLPKVVVGQIHGWKISQALVKLLWEGDNKPVRVILNDNYKLDNNKDCTDCNAFSVKLGTYAVNEDWQYTIRADKEGLFLATYDADGSNMVSHTLKWGEAYSDTANNKSYTLTERWASPDIAFYFKAGIYPQFKPDNAYRGEIFDVSFSALSTLHQ |
| 98 | WP_213871160.1 polysaccharide lyase family 7 protein [Vibrio] | MFIKSTKLLLISFSGLGLIGCGGNTSPTSQDDKPNIPHQEHVAPYSIAKFQNILSNSDLQVSDPNREEGNKNSDVKNGAFSDYKSDHFYAEKDSNYLVFKMSNYKMRSEVRERENFNISEQGVFRTLYADVRLPEINLAMASSPANHDEVTFLQIHNKGTDTSGIGAIPHPLLRIVWEQERNSITGHYWAVVKNNAIDCSLPSSASDCYATSYDRYDLGKADLNAFTRFEVKIGENTLTIKVNDEQKVNVDVSYWQHLLSYFKAGVYNQFENGEAKVQFKQLGLTKTDHTDSIAWNIDDWKLTIPASKNDWYGFGGDSAAELEPERCNSSKDPLSNEESVYQREIDLSYFNVIDGSMHFRADMGYGTSTANSSYIRSELRELYISTNSPDCSTSDEETSWYIEDSRTGATSHTLNATLRINEYPKIDGQLPKVVVGQIHGWKISQALVKLLWKGDNKPVRVILNDNYKLDNNKDCTDCNAFSVKLGTYAVNEDWQYTIRADKEGLFLATYDADGSNMVSHTLKWGEAYSDTANNKSYTLTERWASPDIAFYFKAGIYPQFKPDNAYRGEIFDVSFSALSTLHQ |
| 99 | WP_322141967.1 polysaccharide lyase family 7 protein [Vibrio sp. PBL-C16] | MFIKSTKLLLISFSGLGLIGCGGNTSPTSQDDKPNIPHQEHVAPYSIAKFQNILSNSDLQVSDPNGKEGNKTSDVKNGAFSDYKSDHFYAEKDSNYLVFKMSNYKMRSEVRERENFNISEQGVSRTLYADVRLPEINLAMASSPANHDEVTFLQIHNKGTDTSATGAIPHPLLRIVWEQERNSITGHYWAVVKNNAIDCSLPSSASDCYATSYDRYDLGKADLKAFTRFEVKIGENTLTIKVNDEQKVNVDVSYWQHLLSYFKAGVYNQFENGEAKVQFKQLGLTKTDHTDSIAWNIDDWKLTIPTSKNDWYGFGGDSAAELEPERCNSSKDLLSNEESVYQREIDLSYFNVIDGSMHFRADMGYGTSTANSSYIRSELRELYISTNSPDCSTSDEETSWYIEDSRTGATSHTLNATLRINEYPKIDGQLPKVVVGQIHGWKISQALVKLLWEGDNKPVRVILNDNYKLDNNKDCTDCNAFSVKLGTYAVNEDWQYTIRADKEGLFLASYDADGSNMVSHTLKWGEAYSDTANNKSYTLTERWASPDIAFYFKAGIYPQFKPDNAYRGEIFDVSFSALITLHQ |
| 100 | WP_154203900.1 polysaccharide lyase family 7 protein [Vibrio alginolyticus] | MFIKSTKLLLISFSGLGLIGCGGNTSPTSQDDKPNIPHQEHVAPYSIAKFQNILSNSDLQVSDPNGKEGNKNSDVKNGAFSDYKSDHFYAENDSNYLVFKMSNYKMRSEVRERENFNISEQGVFRTLYADVRLPEINLAMASSPANHDEVTFLQIHNKGTDTSGTGAIPHPLLRIVWEQERNSITGHYWAVVKNNAIDCSLPSSASDCYATSYDRYDLGKADLNAFTRFEVKIGENTLTIKVNDEQKVNVDVSYWQHLLSYFKAGVYNQFENGEAKVQFNQLGLTKTDHTDSIAWNIDDWKLTIPASKNDWYGFGGDSAAELEPERCNSSKDLLSNEESVYQREIDLSYFNVIDGSMHFRADMGYGTSTANSSYIRSELRELYISTNSPDCSTSDEETSWYIEDSRTGATSHTLNATLRINEYPKIDGQLPKVVVGQIHGWKISQALVKLLWEGDNKPVRVILNDNYKLDNNKDCTDCNAFSVKLGTYAVNEDWQYTIRADKEGLFLASYDADGSNMVSHTLKWGEAYSDTANNKSYTLTERWASPDIAFYFKAGIYPQFKPDNAYRGEIFDVSFSALSTLHQ |
