# Supplementary material for: A bifunctional endolytic alginate lyase with two different lyase catalytic domains from Vibrio sp. H204
Source: Front Microbiol. 2024 Dec 13;15:1509599. doi: 10.3389/fmicb.2024.1509599 (PMC11671496; doi:10.3389/fmicb.2024.1509599)
Supplement: Supplementary file 6 [file Table_2.doc]

|  | **Aly35** | | |  | **Aly35-CD1** | | |  | **Aly35-CD2** | | |
| --- | --- | --- | --- | --- | --- | --- | --- | --- | --- | --- | --- |
| **0.02 mole** | **Sodium alginate** | **PM** | **PG** |  | **Sodium alginate** | **PM** | **PG** |  | **Sodium alginate** | **PM** | **PG** |
| activity (units) | 5511.27 | 3055.26 | 3303.81 |  | 1497.82 | 1773.45 | <100 |  | 4416.74 | 952.97 | 4046.46 |

**Table S2 Activities of Aly35, Aly35-CD1 and Aly35-CD2 with equals molar equivalents toward sodium alginate, PM and PG.**
